# Comprehensive characterization of mainstream marijuana and tobacco smoke

**DOI:** 10.1038/s41598-020-63120-6

**Published:** 2020-04-28

**Authors:** Brian M. Graves, Tyler J. Johnson, Robert T. Nishida, Ryan P. Dias, Benjamin Savareear, James J. Harynuk, Mohsen Kazemimanesh, Jason S. Olfert, Adam M. Boies

**Affiliations:** 1 Department of Engineering, University of Cambridge, Trumpington Street, Cambridge, CB2 1PZ United Kingdom; 2 Department of Mechanical Engineering, University of Alberta, Edmonton, Alberta T6G 1H9 Canada; 3 Department of Chemistry, University of Alberta, Edmonton, Alberta T6G 2G2 Canada

**Keywords:** Nanoparticles, Mass spectrometry

## Abstract

Recent increases in marijuana use and legalization without adequate knowledge of the risks necessitate the characterization of the billions of nanoparticles contained in each puff of smoke. Tobacco smoke offers a benchmark given that it has been extensively studied. Tobacco and marijuana smoke particles are quantitatively similar in volatility, shape, density and number concentration, albeit with differences in size, total mass and chemical composition. Particles from marijuana smoke are on average 29% larger in mobility diameter than particles from tobacco smoke and contain 3.4× more total mass. New measurements of semi-volatile fractions determine over 97% of the mass and volume of the particles from either smoke source are comprised of semi-volatile compounds. For tobacco and marijuana smoke, respectively, 4350 and 2575 different compounds are detected, of which, 670 and 536 (231 in common) are tentatively identified, and of these, 173 and 110 different compounds (69 in common) are known to cause negative health effects through carcinogenic, mutagenic, teratogenic, or other toxic mechanisms. This study demonstrates striking similarities between marijuana and tobacco smoke in terms of their physical and chemical properties.

## Introduction

Cannabis is among the most commonly used controlled substances worldwide^1,2^. An estimated 192.2 million people used cannabis in 2016, corresponding to 3.9% of the world’s population aged 15–64^2^. In North America, an estimated 12.9% of people in this age group used the drug in 2016 and the rate of use is increasing^2^.

There is a growing trend to liberalize policies governing cannabis possession and use^3^ with over 20 countries and most U.S. states legalizing cannabis use for medicinal purposes within the last decade^4^. At the time of writing, eleven U.S. states, starting in 2012, have legalized marijuana for recreational use by adults^5^. In 2018, Canada became the first Group of 7 (G7) country^6^ and the second in the world, after Uruguay^7^ in 2013, to formally legalize cannabis for recreational use^8^.

In Canada, 94% of recreational cannabis users in 2017 reported consuming the drug by smoking marijuana^9^, a preparation of dried cannabis flowers and leaves^1^. Smoking marijuana is commonly perceived as less harmful than smoking tobacco^10,11^. However, marijuana smoke contains harmful substances including known carcinogens likely emitted from the pyrolysis of the plant material during smoking^12^.

The health effects of tobacco smoke have been extensively studied and after decades of research it has been classified as a Group 1 carcinogen^13^. While smoking marijuana has been associated with increased rates of adverse respiratory symptoms and chronic obstructive pulmonary disease^10,14^, it has not been conclusively linked to lung cancer^15^.

The chemical composition of tobacco smoke has been thoroughly investigated in previous work^16^. However, there are few reports of the chemical composition of marijuana smoke. The chemicals emitted from smoking tobacco cigarettes or marijuana cigarettes (known as joints) are qualitatively similar with some quantitative differences^17–19^. Chemicals such as nitrogen oxides, hydrogen cyanide, and aromatic amines were found in marijuana smoke at concentrations three to five times higher than tobacco smoke^17^. The total particulate matter (TPM) and ‘tar’ commonly associated with tobacco smoke, is also found in similar or higher concentrations in marijuana smoke^17,20^.

Aerosols (solid and/or liquid particles suspended in a gas) are present in concentrations higher than 10^9^ particles cm^−3^ in fresh smoke from tobacco cigarettes^21–23^. The deposition of chemical constituents from an aerosol in human lungs (e.g. by impaction or diffusion)^24^ depends on the aerosol particle characteristics such as aerodynamic and mobility diameters, density, and volatility^25–28^. Several studies characterize aerosols from smoking tobacco cigarettes^27,29–32^; however, there are very few studies which characterize aerosols from smoking marijuana joints. In 1975, Hoffman *et al*.^19^ found higher total dry particulate matter in sidestream tobacco smoke compared with sidestream marijuana smoke. In the 1980s, the group of Hiller *et al*.^33,34^ measured the aerodynamic size distribution and mobility size distribution of smoke from marijuana cigarettes using a laser-based method operating in the 0.3–6 μm size range and an electrical aerosol analyzer, respectively, finding quantitative similarities between smoke from tobacco cigarettes and marijuana joints. The chemical properties of mainstream and sidestream smoke from nonfiltered tobacco or marijuana cigarettes under two smoking conditions were extensively compared by Moir *et al*.^17^. However, aerosol research in this area appears to be limited. To the authors’ knowledge, with the exception of recent TPM measurements^17,35^, the aerosol properties of marijuana smoke have not been characterized or compared with those of tobacco smoke since 1989^34^ despite drastic improvements in analytical instrumentation and the increase in marijuana consumption. Therefore, significant research is required to generate the same level of understanding of marijuana smoke that has been developed of tobacco smoke over decades.

In this work, aerosol particles produced from smoking tobacco cigarettes or marijuana joints are characterized in terms of particle number concentration, aerodynamic and mobility size distributions, mass, effective density, morphology, volatility, and chemical composition. These characteristics are then quantitatively compared with each other as well as against previous tobacco research to provide context for the marijuana smoke results, an area where knowledge is currently limited.

## Results

This study compares the mainstream smoke produced from a filtered tobacco cigarette with that from a nonfiltered marijuana joint. These methods represent the most common consumption of tobacco cigarettes and marijuana joints (greater than 97% as discussed in Methods, and Supplementary Section S1). The aerosol smoke samples were collected in a bag with dilution air to allow common particle characterization techniques to be utilized, while the chemical composition and total particulate matter (TPM) measurements were completed on smoke collected on quartz filters immediately downstream of the cigarette or joint. These two sampling methods, henceforth referred to as aged and fresh smoke, respectively, were identical between the measurements of the tobacco and marijuana smoke. Further experimental details are provided in the Methods section, with additional information on the cigarettes and sampling techniques presented in Supplementary Sections S1 and S2, respectively.

Smoke particles contain chemical compounds with a large range of volatilities. To investigate this aspect, this study used a catalytic stripper strategically with different experimental setups to characterize the nonvolatile portion of the smoke (e.g. particle sizes and concentrations), and to provide insights into the chemical composition of the aerosol (e.g. inferences based on the effective densities before and after stripping, and the semi-volatile fractions). Semi-volatile compounds have a meaningful presence in both gas and particulate phases, and have lower vapor pressures than volatile compounds^36^. By the operational definition in this work, “semi-volatile” refers to compounds which fully evaporate at 350 °C within a few seconds or less^37^. Stripped particles were passed through a catalytic stripper at 350 °C to remove the semi-volatile components, leaving only the nonvolatile components of the aerosol. Nonstripped particles were not conditioned by a catalytic stripper.

### Aerosol size distributions and concentrations

**Figure 1:**
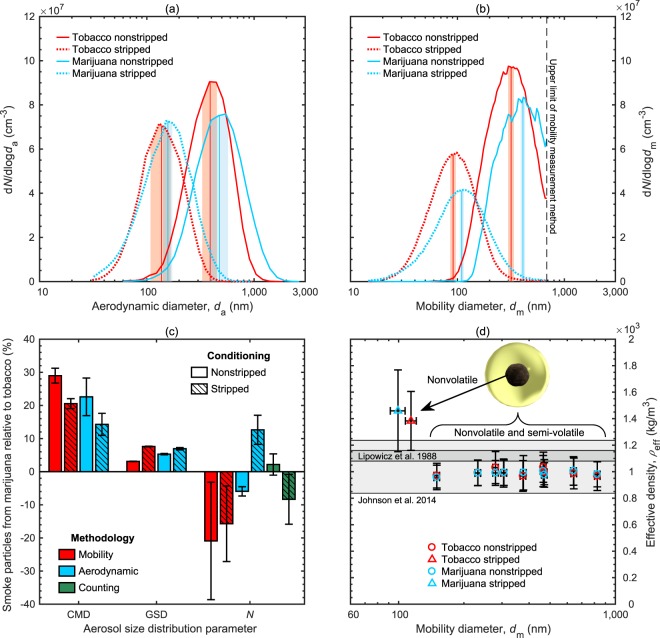
(**a**) Averaged aerodynamic size distributions where nonstripped, aged aerosols from smoking marijuana joints or tobacco cigarettes had average count median diameters (CMDs) of 477 nm (±95 nm) or 389 nm (±61 nm), respectively, while catalytic stripping the aged aerosols produced average CMDs of 157 nm (±15 nm) or 137 nm (±29 nm), respectively. Geometric standard deviations of the averaged log-normal fits ranged between 1.57 and 1.69. (**b**) Averaged mobility size distributions where nonstripped, aged aerosols from smoking marijuana joints or tobacco cigarettes had average CMDs of 410 nm (±20 nm) or 318 nm (±19 nm), respectively, while catalytic stripping the aged aerosols produced average CMDs of 110 nm (±5 nm) or 91 nm (±5 nm), respectively. Geometric standard deviations of the averaged log-normal fits ranged between 1.63 and 1.75. (**c**) Percent differences between size distribution parameters for either nonstripped or stripped aged smoke particles from a marijuana joint relative to a tobacco cigarette. (**d**) Effective densities of nonstripped and stripped aged smoke particles. For (**a**,**b**), the center line and shaded regions represent each CMD and its corresponding total uncertainty, while for (**c**,**d**) the error bars represent the total uncertainties, with the exception of GSD error bars in (**c**) which represent its precision uncertainty.

The aerodynamic and mobility size distributions of aged particles produced by smoking tobacco cigarettes or marijuana joints are qualitatively similar, as shown in Fig. 1a and 1b, with some small quantitative differences as summarized in Fig. 1c. All size distribution measurements confirm a lognormal frequency characteristic of aerosols having undergone coagulation by Brownian motion^38^. The lognormal size distributions are quantified by three parameters: count median diameter (CMD), geometric standard deviation (GSD) and total number concentration (*N*). Further detail is given in the Methods and Supplementary Sections S3.1 and S3.2.

For nonstripped samples, the aged particles of marijuana smoke were larger overall than those of tobacco smoke with the CMDs being on average 23% (±6%) and 29% (±2%) larger for the aerodynamic (477 vs 389 nm) and mobility (410 vs 318 nm; based on an average of all five mobility distribution scans collected consecutively) size distributions, respectively. These diameters and trends agree with previous aerodynamic diameter measurements of marijuana and tobacco smoke particles of 350–430 nm and 380 nm, respectively^33^. However, for aged particles from tobacco smoke the 318 nm measured mobility CMD is larger than the 234 nm mobility CMD previously measured^30^. This variation between studies may be due to the different puff routines used to generate the tobacco smoke (HCI vs ISO), aging of smoke particles over the puff routine (4 vs 8 mins) and dilution ratios in the smoke bags (75 vs 33). Adam *et al*.^29^ found the mobility CMD of fresh tobacco smoke varied between 170 and 290 nm with the CMD decreasing with increasing puff number, higher puff inhalation rates, or lower cigarette ventilation. Similarly, Ingebrethsen *et al*.^39^ found that the mobility CMD of fresh tobacco smoke decreased with increasing puff number or puff inhalation rates using two different measurement techniques: electrostatic classification (CMD range: 184 to 217 nm) and spectral transmission (CMD range: 228 to 337 nm).

Similar to the nonstripped aerosol samples, the aged particles in stripped marijuana smoke were larger overall than those in stripped tobacco smoke with the CMDs being on average 14% (±3%) and 21% (±1%) larger for the aerodynamic (157 vs 137 nm) and mobility (110 vs 91 nm; based on an average of all five mobility distribution scans collected consecutively) size distributions, respectively. In either case (i.e. nonstripped or stripped), the particle aerodynamic or mobility size distributions of aged marijuana smoke were consistently slightly broader than those produced by aged tobacco smoke. This difference is reflected in the geometric standard deviations (GSDs), ranging from 1.57 to 1.79, that were on average 5.4% higher for the marijuana smoke size distributions than those of tobacco smoke. The GSDs of both smoke aerosols are higher than the steady-state GSD of 1.46 for aerosols that have achieved self-preserving size distributions^40^, indicating the distributions are still evolving over the duration of the smoke inhalation.

The total particle number concentrations (*N*) of the aged aerosol from either smoke source were quantified using three different methodologies as shown in Fig. 1c. These measurements varied due to the high concentration of particles in the smoke samples and their transient nature (see Supplementary Section S3.2 for further details). Despite this variation and the measured sizes of the tobacco and marijuana smoke particles being different, the particle number concentrations from the two smoke sources are approximately the same with four of the six measurements agreeing within the measurement uncertainty as shown in Fig. 1c, and all six measurements agreeing within ±20% for both the nonstripped and stripped smoke samples. The dilution-corrected particle number concentrations measured by the condensation particle counter (CPC) for both the aged tobacco and marijuana smoke are shown in Supplementary Fig. S3. These CPC-measured concentrations from either smoke source decreased over the 15 min sampling period from approximately 7.5 × 10^7^ to 3.1 × 10^7^ particles cm^−3^, and from approximately 7.2 × 10^7^ to 2.8 × 10^7^ particles cm^−3^, for the nonstripped and stripped particles, respectively. This decrease in particle concentration over time is also reflected in the consecutive mobility size distribution measurements (see Supplementary Fig. S4) and, as discussed further in Supplementary Section S3, is likely a combination of particle coagulation, evaporation, and losses within the smoke bag over time^30^.

### Effective density and mass

The effective density of an aerosol particle relates its mobility diameter and mass, properties which govern the particle’s trajectory (through diffusion and impaction), and quantity of non-gaseous chemicals delivered during inhalation. This density parameter also provides insights into a particle’s shape, whereby a homogeneous, spherical particle has a constant effective density equivalent to its material density and a fractal-like particle, such as soot, has a size-dependent effective density lower than its material density. The measured effective densities of aged particles from tobacco and marijuana smoke are shown in Fig. 1d. The effective densities of the nonstripped particles from either smoke source are between 957 kg m^−3^ and 1033 kg m^−3^, and agree within their measurement uncertainty at a 95% confidence interval as depicted by their error bars. Densities in this range are common for organics^41^. These results also agree within uncertainty with effective densites of aged particles from tobacco smoke determined independently by Johnson *et al*.^30^ (1037 ± 200 kg m^−3^) and Lipowicz *et al*.^42^ (1120 ± 40 kg m^−3^). The effective densities measured here are independent of particle diameter, with averages of 993 kg m^−3^ (±94 kg m^−3^) and 987 kg m^−3^ (±93 kg m^−3^) for tobacco and marijuana smoke particles, respectively. This constant density indicates that the nonstripped, aged particles from either smoke source have a spherical morphology, which is likely achieved by the outer surface of each particle being liquid. This inference agrees with the chemical composition and particle size distribution results that indicate the presence of relatively volatile hydrocarbons, which likely exist as liquids.

Similarly, the measured effective densities of 470 nm particles, a size near the median of the mobility size distribution, from aged tobacco and marijuana smoke conditioned using a catalytic stripper to remove any semi-volatile material were 1383 kg m^−3^ (±222 kg m^−3^) and 1459 kg m^−3^ (±309 kg m^−3^), respectively. The mobility diameters of these stripped tobacco and marijuana smoke particles were 114 nm (±7 nm) and 99 nm (±8 nm), respectively. Densities in this range are substantially higher than the average density of the nonstripped smoke particles (≈990 ± 94 kg m^−3^), and are common for many higher-molecular weight organics^41^.

The marijuana smoke particles were on average 29% larger in mobility diameter and 5.2% larger in geometric standard deviation, and similar in both number concentration and effective density. Using those parameters and their associated uncertainties, the mass concentrations of the particles were roughly 2.5 (±0.7) times higher in the aged marijuana smoke than the aged tobacco smoke. This estimate uses the Hatch-Choate equations to calculate the particle mobility diameter that represents the average mass of the measured mobility size distributions fitted with a log-normal function. These mass concentrations agree with total particulate matter (TPM) measurements of fresh smoke collected on a filter directly downstream of the cigarette or joint (without dilution or aging due to sampling), which show smoking a marijuana joint produces roughly 3.4 (±0.6) times more TPM than a tobacco cigarette following the same HCI routine with six puffs.

### Semi-volatile fractions

For both the polydispersed particle size distributions and particles at one representative size (mobility diameter of 470 nm), the average semi-volatile mass and volume fractions are in excess of 97% as summarized in Table 1. These results indicate that the volatility of aged particles from either smoke source are similar and that the particles are almost entirely comprised of semi-volatile material. Based on the upstream CPC measurements, the total semi-volatile fraction in terms of particle number was found to be <20% from either smoke source, indicating that most particles are heterogeneous containing both semi-volatile and nonvolatile components. Particles with high mass volatility can also be produced from combustion engines^43^ although this is typically accompanied by higher number-based semi-volatile fractions than are observed here. Furthermore, purely semi-volatile particles may manifest as another distinct peak in a particle size or mass distribution^44^, however, all size and mass distributions measured were uni-modal.

**Table 1:** Semi-volatile fractions of polydispersed and monodispersed (470 nm mobility diameter) smoke particles.

Smoke from	Polydispersed Particles Semi-volatile Fractions (%)			Monodispersed Particles Semi-volatile Fractions (%)	
	Number	Volume	Mass	Volume	Mass
Tobacco	7	98	97	99	98
	(±6)	(±27)	(±33)	(±10)	(±21)
Marijuana	17	98	97	99	99
	(±8)	(±23)	(±32)	(±11)	(±32)

### Chemical analyses

**Figure 2:**
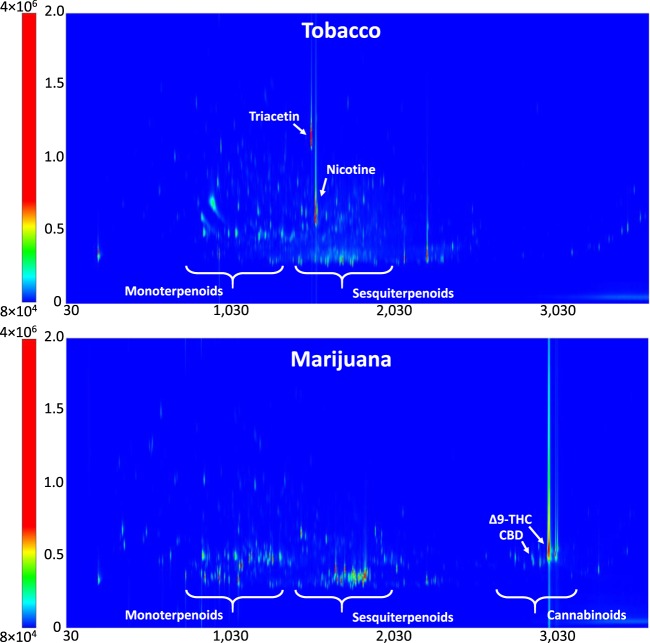
SPME-GC×GC-TOFMS chromatograms of the particulate phase fraction of mainstream smoke from tobacco and marijuana. The x-axis denotes first-dimension retention time (seconds), while the y-axis denotes second-dimension retention time (seconds). Peak intensity is indicated based on the colour bar.

Headspace solid-phase microextraction (SPME) was used to sample components of aerosols collected directly from mainstream tobacco and marijuana smoke on quartz filters for chemical analyses. Extracted compounds were then characterized by comprehensive two-dimensional gas chromatography with time-of-flight mass spectrometric detection (GC×GC-TOFMS). Figure 2 depicts typical chromatograms for both tobacco and marijuana smoke with some regions/compounds of interest indicated. The total number of compounds detected for these samples were 4350 and 2575, respectively, which are approaching the over 6000 compounds that have been compiled for tobacco smoke using numerous methods^16^. Based on linear temperature-programmed retention indices of alkanes ranging from C5–C30 in the first dimension and mass spectral library searches against the NIST and Wiley mass spectral libraries, 668 or 534 compounds were tentatively identified in aerosols from tobacco cigarette or marijuana joint smoke, respectively. The identified compounds were further grouped into chemical classes (Supplementary Table S1) to highlight major chemical differences between tobacco and marijuana smoke. The lists of compounds identified in tobacco or marijuana smoke particles along with the known health effects of each compound are also provided in Supplementary Tables S3 and S4.

**Figure 3:**
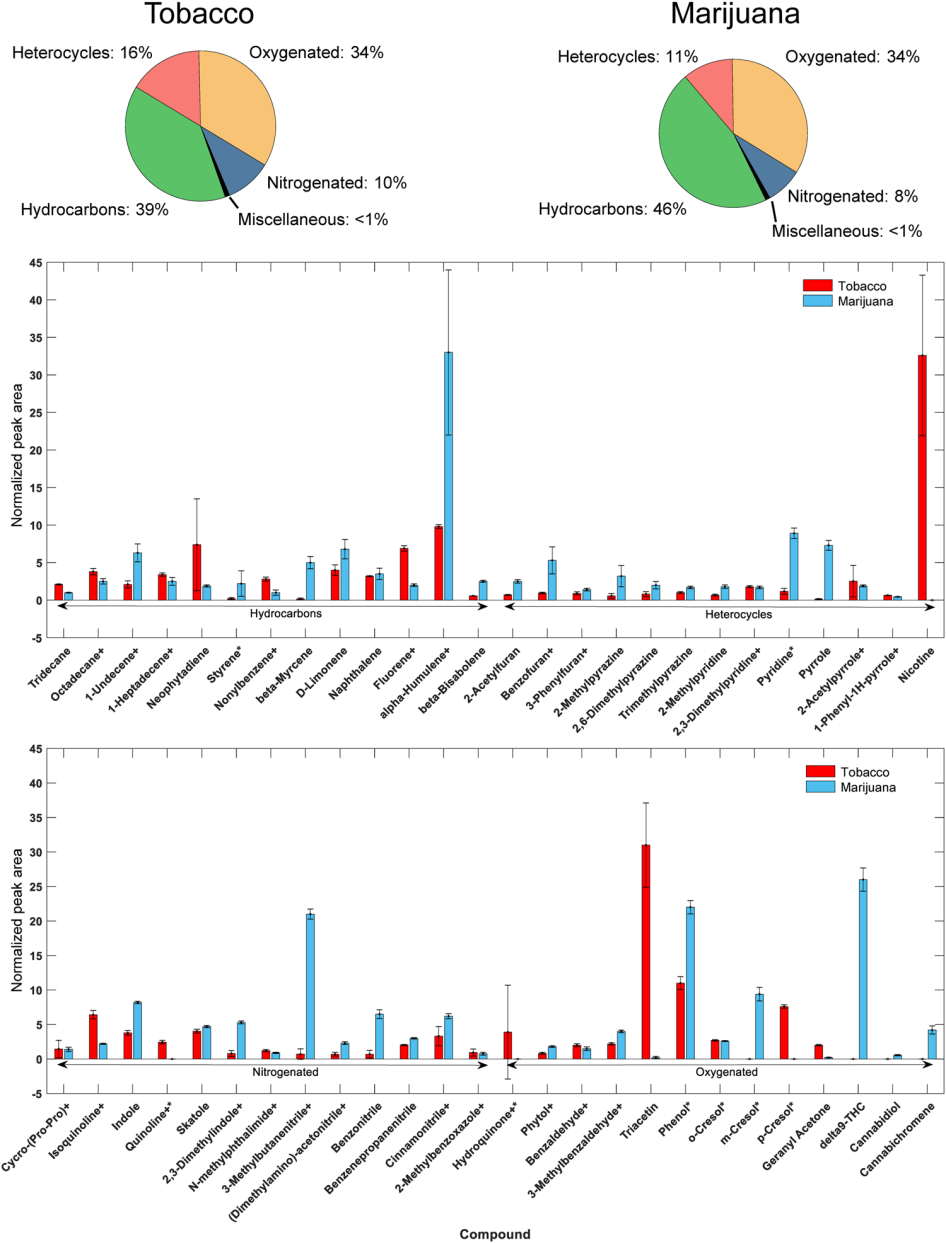
Relative peak distribution of compounds based on the number of peaks detected in the particulate phase fraction of mainstream tobacco or marijuana smoke, along with the average relative peak area (*n* = 3) of selected analytes. These peak areas were normalized to the peak area of dodecane. The error bars represent one standard deviation, while the plus sign (+) denotes compounds with a response multiplied by 10× and the asterisk (*) highlights compounds which are members of the Hoffmann List of biologically and toxicologically active compounds found in tobacco cigarette smoke^46^.

The relative number of peaks among chemical groups for samples of tobacco and marijuana smoke are shown in Fig. 3 (top). Though the two types of smoke look similar according to Fig. 3 (top), the distribution of peaks within the classes exhibit some marked differences, as shown in Fig. 3 (bottom) and as detailed in Supplementary Table S1. Most notably, the hydrocarbon content of tobacco has greater contributions from aromatic and polycyclic aromatic compounds, whereas marijuana contains more terpenes and sesquiterpenes. Additionally, tobacco contains a greater variety of pyridines than marijuana, even though marijuana smoke itself contains about seven times more pyridine than tobacco cigarette smoke. A greater number of oxygenated species are observed in tobacco smoke, which may be due to differences in oxygenated species endogenous to the product, or could be due to compounds being produced in greater amounts during the tobacco cigarette combustion process itself.

229 compounds, tentatively identified using GC×GC-TOFMS, were found to be common to both types of smoke samples. Health effects of the individual compounds that were tentatively identified are summarized in Table 2, and detailed in Supplementary Tables S3 and S4. This list of compounds represents only those identified by the chemical analyses in this work, and should not be considered an exhaustive list of carcinogens, mutagens, teratogens or otherwise toxic compounds found in mainstream tobacco or marijuana smoke^45^. These health effects were classified using the International Agency for Research on Cancer (IARC) carcinogen list and the Registry of Toxic Effects of Chemical Substances (RTECS) provided by the Canadian Centre for Occupational Health and Safety (CCOHS).

**Table 2:** Summary of known health effects for compounds found in smoke particles from tobacco cigarettes and marijuana joints. Numbers indicate the number of tentatively identified compounds which exhibit a given health effect.

Health Effect	Tobacco	Marijuana	Common
Group 1 carcinogen*	1	1	1
Group 2A carcinogen	2	2	2
Group 2B carcinogen*	11	9	8
Group 3 carcinogen	15	14	11
Mutagen	70	50	28
Teratogen	0	1	0
Toxic by other mechanisms	74	33	19
No risk data available**	497	426	162
Total detected by GC×GC-TOFMS	668	534	229
Total detected by both methods*	670	536	231

*Many Group 1 and 2 carcinogens known in tobacco smoke are either too volatile or not volatile enough to be observed using the approach used herein. Therefore, this list of compounds and their health effects should not be considered to be an exhaustive list of carcinogens, mutagens, teratogens or otherwise toxic compounds found in mainstream tobacco or marijuana smoke. In parallel to the GC×GC-TOFMS results, formaldehyde (Group 1) and acetaldehyde (Group 2B) were detected by a third-party laboratory and included within the table. See text and Supplementary Table S2 for further discussion.

**No risk data available or risks mitigated by proper protective equipment.

The data in Table 2 show some notable differences between the potential health effects of tobacco vs. marijuana smoke in terms of the variety of compounds present. Of the 110 compounds posing some health risk in marijuana, 69 (63%) are common to tobacco, and tobacco has 173 compounds posing known health risks. A limitation of the chemical analyses and sampling of the collected aerosols from the filters is that very light compounds are not detectable by our approach. For example, formaldehyde and acetaldehyde, Group 1 and Group 2B carcinogens, respectively, were undetected by GC×GC-TOFMS, but were detected in both tobacco and marijuana smoke with additional analyses by a third-party laboratory. These two additional compounds are included in the health risk numbers above, as well as Table 2. Please see Supplementary Table S2 comparing results between the GC × GC-TOFMS and the third-party laboratory results. In order to appear in our results, compounds must have a sufficiently low vapor pressure to condense into the particle phase and be trapped by filters during the smoking experiment. Subsequently, their vapor pressure must be high enough at the extraction temperature that they can migrate from the filter to the headspace and then to the SPME fibre, while also having a sufficiently high partition coefficient driving them into the SPME fibre in order for sufficient mass of a compound to be concentrated for introduction to the GC×GC-TOFMS. In general, compounds in the range of C6 (hexane) to C25 (pentacosane) are readily observed under the conditions of this experiment. A second limitation of the non-target approach taken here is that different compounds will have different partition coefficients with the SPME fibre, and MS response factors are not constant across all compounds. This means that comparisons between different compounds are impossible in all but the most general terms. However, comparisons in the relative amounts of a particular compound (e.g. triacetin) in tobacco vs. marijuana are easily made.

While relative concentrations of compounds in the two types of aerosol can be estimated based on careful study of Supplementary Tables S3 and S4, an actual assessment of risk would also need to consider other factors such as the dose being received through smoking. As discussed in Supplementary Section S2, marijuana smokers tend to inhale larger volumes of smoke and also hold the smoke in their lungs longer than tobacco smokers^17,20,47–50^, which may lead to a different proportion of inhaled material entering the bloodstream. However, tobacco users typically smoke many more cigarettes per day than marijuana users smoke joints. In Canada, for example, the average smoker of tobacco will consume 13.7 cigarettes per day^51^ (just over half a pack or ≈390 cigarettes per month). This is in stark contrast to usage patterns for marijuana where 55% of users in Canada report using it three times or less per month, and only 19% of users report using it daily. Additionally on a “use day”, marijuana users will typically consume ≈1 g of marijuana (equivalent to two of the pre-rolled joints used in this study)^52^. Therefore, the data presented here should be viewed as a guide to compounds (and their metabolites) that should be targeted in future health studies.

## Discussion

The physical characteristics of aerosol particles produced by smoking tobacco cigarettes or marijuana joints are qualitatively similar with quantitative differences in size, mass and chemical composition. Smoking a marijuana joint produces larger particles than a tobacco cigarette on average as reflected by the 23% (±6%) and 29% (±2%) larger aerodynamic and mobility count median diameters, respectively. The primary mechanisms of particle deposition in human lungs include diffusional deposition and inertial impaction which are governed by a particle’s mobility and aerodynamic diameter, respectively. Diffusion is the primary deposition mechanism for particles smaller than 0.1 μm in mobility diameter^53^ and occurs mostly in the alveoli of the lungs^26,44^. Inertial impaction is an important deposition mechanism for particles with aerodynamic diameter greater than 1 μm^53^ and occurs mostly in the upper airways^26,44^. Therefore, the measured differences in particle size between marijuana and tobacco smoke could have limited, but potentially significant implications for locations of deposition of the chemicals they carry into human lungs.

Similarities between tobacco and marijuana smoke particles include roughly the same number concentrations (≈±20%) and similar average effective densities (993 vs. 987 kg m^−3^, respectively). These effective particle densities are independent of mobility size, indicating that the particles from either smoke source are spherical. This morphology is likely due to the particles having a liquid component, which agrees with other volatility and chemical measurements that indicate the presence of light hydrocarbons. These similarities in morphology, effective density and number concentration, while accounting for the marijuana smoke particles being larger, results in a 2.5 (±0.7) times higher mass concentration of aged particles in marijuana smoke than tobacco smoke. This estimate agrees within uncertainty with total particulate matter (TPM) measurements of fresh smoke also collected, which shows smoking a marijuana joint produces roughly 3.4 (±0.6) times more TPM than smoking a tobacco cigarette following the same puff routine.

It is also demonstrated for the first time that both aged tobacco and marijuana smoke are comprised almost entirely of semi-volatile chemical species (over 97% in terms of both particle volume and mass). This result agrees with chemical analyses of fresh tobacco and marijuana smoke collected on filters. The chemical analyses tentatively identified 536 and 670 compounds in marijuana and tobacco smoke particles, respectively, with approximately one-third being common to both smoke sources. Of those identified, 110 and 173 compounds found in marijuana and tobacco smoke (69 common to both), respectively, are known to pose health risks through carcinogenic, mutagenic, teratogenic or other toxic mechanisms. While there are compounds in marijuana which may have some therapeutic effects, these have not been thoroughly and rigorously studied in this work. Consequently, this study focuses on compounds which present known health risks and could act as a guide to compounds (and their metabolites) that should be targeted in future health studies.

While this study characterized and compared the mainstream smoke from marijuana joints and tobacco cigarettes most representative of that encountered by the general public (i.e. filtered tobacco cigarettes and nonfiltered marijuana joints), previous studies, as summarized in Hoffmann & Hoffmann^54^, indicate that filters on tobacco cigarettes are effective at reducing both total particulate matter (TPM) and nicotine delivery^55^. Therefore, additional insights could be gained by future studies of the smoke from filtered marijuana joints, such as if any of the similarities or differences between the tobacco and marijuana smoke observed in this study are due to being filtered and nonfiltered, respectively.

The aerosol properties of fresh smoke from marijuana relative to tobacco could also be compared using techniques which exhibit faster response times. These techniques are associated with higher uncertainties that must be carefully addressed, but will better capture the volatile and transient behaviour of the smoke particles. These techniques will also avoid the challenges and biases introduced when generating a “steady-state” aerosol sample, specifically multiple transfers of the smoke samples between bags and dilution with HEPA-filtered air. The decreased latency times would also be more representative of the aerosol that is inhaled by the smoker.

To assess some of the effects of the steady-state sampling on the smoke characteristics, the diffusion and settling losses of the particles in the sample bag during testing were estimated and found to be insignificant to the lognormal parameters (<5.5% change in CMD, GSD or *N*) of the smoke size distributions. These negligible losses of particles also agree with the insignificant diffusion and settling losses estimated by Johnson *et al*.^30^ for nonstripped tobacco smoke in a 10 L sample bag. Since these loss estimates are conservative, based on simplifying assumptions and negligible relative to the other uncertainties of the measurements (as summarized in the Statistical Analysis section), these loss corrections were not applied to the results presented in this study. Please see Supplementary Section S3 for further details.

Despite negligible particle losses of nonstripped tobacco smoke, Johnson *et al*.^30^ found the particle concentration in the sample bag decreased over time. They showed that particle coagulation is likely the main mechanism for this trend, which should increase the CMD of smoke particles. However, consecutive mobility size distribution measurements collected by Johnson *et al*.^30^ showed a negligible increase in the particle diameter (<2.7% shift from the average CMD over 12 minutes). This discrepancy was explained, based on mass conservation, by components of the particles likely evaporating over time. These results agree with the measurements of this study, which observed the decreasing particle concentration in the sample bag for all of the smoke samples (as shown in Supplementary Fig. S3). The diffusion and settling losses of the particles in the sample bag were also negligible (<5.5% change) and there was no significant increase in the particle diameter (<3.1% or <5.4% shift from the average CMD for tobacco or marijuana smoke over 15 minutes, respectively). For example, the consecutive mobility scans of nonstripped tobacco smoke are shown in Supplementary Fig. S4. This inference of particle evaporation is further supported by other results of this study, specifically that a portion of the aged smoke particles are likely liquid due their spherical morphology, the effective density of the particles is common for organics and the high semi-volatile fractions (≥97%) of the particles. Therefore, components of the particle evaporating over time likely affected the representativeness of the aged smoke samples of this study. However, particle evaporation has also been observed in fresh tobacco smoke^32,56^, and this observation is further supported by the many volatile and semi-volatile compounds identified in the fresh smoke of this study.

In summary, the characterization of marijuana smoke presented comprises particle, chemical, and volatile species analyses, while using parallel tobacco smoke measurements and existing literature to provide context. These results provide a foundation for investigating other parameters, such as the effects of different smoking patterns^20^, cannabis strains, exposure paths (second^57^ or third-hand^58^), and cigarette/joint design (dimensions or filtered vs nonfiltered). Building on our work, researchers have a basis for which chemical compounds and particle properties to target in future toxicology or lung deposition studies of marijuana smoke to determine its associated health effects.

## Methods

### Experimental design

The objective of this study was to characterize marijuana smoke and contrast it against its well-understood analog - tobacco smoke - under identical testing conditions. This was accomplished by completing experiments at the University of Alberta’s Department of Mechanical Engineering in January of 2019. Tobacco cigarettes and marijuana joints were smoked using a dedicated smoking machine which allowed for programmable smoking routines, including the ability to vary the puff volume, profile and timing. For the online aerosol measurements, smoke was collected in sample bags which were pre-filled with dilution air and discarded after a single use. Material for offline measurements was collected using filters positioned immediately downstream of the tobacco cigarette or marijuana joint.

### Smoke generation

The smoke samples were produced using a smoking machine (Cambustion Ltd., UK) using a standard puff routine (Health Canada Intense, 55 mL puff of 2 s duration, every 30 s^59^) with either filtered reference tobacco cigarettes (University of Kentucky; 3R4F)^60^ or nonfiltered marijuana joints (Aurora Cannabis Inc.; type ACES). These products represent the most common method of consumption of tobacco cigarettes and marijuana joints. In 2016, 99.7% of the cigarettes purchased in the United States were filtered^61^, while approximately 99% of the pre-rolled marijuana joints currently available from provincial dispensaries in Canada are nonfiltered (i.e. cardboard tipping paper). Further details of these consumption methods are discussed in Supplementary Section S1.

The mainstream smoke produced from one cigarette or joint was either captured directly into quartz filters for chemical analyses or TPM measurements, or collected into a smoke bag (Kite Packing, Coventry, UK) from which aerosol characterization was performed. These two sampling methods are referred to as aged and fresh smoke, respectively. The aged samples are representative of smoke samples transferred twice during generation, diluted by a factor of 75 with HEPA filtered air and aged over the 4 min Health Canada Intense (HCI) puffing routine^59^ before being characterized. These aged samples likely differ in some aspects due to particle coagulation, evaporation and losses in the smoke bag^30^ from the aerosol inhaled during smoking. However, the particle losses within the sample bag were found to be negligible (<5.5%), and the handling, dilution, and aging processes were identical between the measurements of the aged tobacco and marijuana smoke, thus allowing a fair and unbiased comparison between the two smoke sources. Additional details regarding the cigarette samples, smoke generation/sampling and aged particle losses are outlined in Supplementary Sections S1–S3, respectively.

### Aerosol size distributions and concentrations

An aerosol is commonly characterized by its distribution of particle sizes and total particle number concentration. In this work, an Aerodynamic Aerosol Classifier (AAC, Cambustion Ltd.) and a Differential Mobility Analyzer (DMA, TSI Inc.) were used to classify particles based on their aerodynamic (*d*_a_) and mobility diameters (*d*_m_), respectively. The aerodynamic (-equivalent) diameter governs a particle’s impaction behaviour, such as during inhalation in the mouth and throat. It is the diameter of a spherical particle with unit density (1 g cm^−3^) that has the same settling velocity as the particle under consideration. The mobility (-equivalent) diameter governs a particle’s diffusion. It is the diameter of a spherical particle with the same mobility or same aerodynamic drag under a known external force as the particle under consideration^62^. The particle concentration at each DMA or AAC setpoint was measured using a CPC and the size distribution was calculated from these raw measurements following Wang & Flagan^63^ or Johnson *et al*.^64^, respectively. Further details regarding the aerosol size distribution measurements are outlined in Supplementary Section S3.1.

The total particle number concentration was determined using three different methodologies, directly with a CPC or by integrating the area under the aerodynamic or mobility size distributions. Further details regarding these different approaches and the sources of variability for these measurements are outlined in Supplementary Section S3.2.

### Effective density and mass

The effective density of a particle (ρ_eff_) provides insights into the particle’s composition and morphology^65^, and is determined by dividing its mass (*m*) by its mobility-equivalent volume (*v*_m_)^66^:

1 $${\rho }_{{\rm{e}}{\rm{f}}{\rm{f}}}=\frac{m}{{v}_{{\rm{m}}}}=\frac{m}{\frac{\pi }{6}{d}_{{\rm{m}}}^{3}},$$

where *d*_m_ is the particle mobility diameter. This definition results in a constant effective density for homogeneous, spherical particle of any mobility diameter. The mass of the individual smoke particles were measured with a DMA, Centrifugal Particle Mass Analyzer (CPMA, Cambustion Ltd.), and CPC in series as first used by others^66,67^, and later used to measure the effective density of tobacco smoke particles^30^. Further details regarding the particle effective density measurements are outlined in Supplementary Section S3.3.

This effective density, combined with the particle mobility diameter that represents the average mass of the measured mobility size distributions as estimated by the Hatch-Choate equations^44^, allowed the total mass concentration of the aerosol to be estimated. This estimate was compared against the total particulate matter (TPM) collected from fresh smoke on a filter directly downstream of the cigarette or joint (i.e. avoiding dilution or aging). Further details of the TPM measurements are outlined in Supplementary Section S3.4.

### Semi-volatile fractions

The semi-volatile mass (*f*_m_) and volume (*f*_v_) fractions indicate the fraction of semi-volatile material relative to the total mass and volume, respectively. This study defines semi-volatile as particle material that is readily removed by a catalytic stripper at 350 °C. These semi-volatile fractions provide insights into the overall composition of the particles, as semi-volatile particles from combustion sources are likely comprised of organic hydrocarbons^43^.

The measurements were completed at a particle size (470 nm mobility diameter) near the median of the distributions, as well as for the entire polydispersed aerosol source. The semi-volatile mass fractions (*f*_m_) of a 470 nm smoke particle were determined using the nonstripped (*m*_ns_) and stripped (*m*_cs_) particle masses measured by the CPMA (*f*_m_ = 1 − *m*_cs_/*m*_ns_). Similarly, the semi-volatile volume fractions (*f*_v_) of a 470 nm particle were determined using the nonstripped (*v*_m,ns_) and stripped (*v*_m,cs_) particle mobility-equivalent volumes measured by a DMA (*f*_v_ = 1 − *v*_m,cs_/*v*_m,ns_).

The semi-volatile fractions for the polydispersed size distributions were determined following a similar methodology, however using the mass and volume concentrations of the aerosols estimated using the Hatch-Choate equations^44^ and measured mobility size distributions, rather than the individual particle mass and volume. Further details regarding the aerosol volatility measurements are outlined in Supplementary Section S3.5.

### Chemical composition

Chemical compounds from particulates captured on pre-fired quartz filters were sampled by solid phase microextraction (SPME) for analyses. Of four different SPME sample fibres tested, Divinylbenzene/Carboxen/Polydimethylsiloxane (DVB/CAR/PDMS) fibres were selected for use in the final study, since the fibres show better extraction efficiency towards a larger number of analytes with diverse chemical functionalities. Chromatograms of both types of smoke samples extracted with different SPME fibre types are shown in Supplementary Fig. S6.

Tentative identification of chromatographic peaks was carried out by searching mass spectra against NIST and Wiley mass spectral libraries (>750/1000 match required) and first-dimension linear temperature-programmed retention indices (LTPRI; ±10 required) matches. For a limited number of cases, mass spectral match (>750 MS match) alone was considered because of the lack of LTPRI data in the available databases. Peaks that did not meet the aforementioned criteria were treated as unknowns. Further details regarding the chemical analyses are outlined in Supplementary Section S4, including HS-SPME-GC×GC-TOFMS parameters (Supplementary Section S4.1) and SPME fibre selection (Supplementary Section S4.2).

### Statistical analysis

All of the uncertainties stated or shown in this study are the total uncertainty based on propagating the repeatability of the measurements and biased uncertainty of the measurement methods through the analysis. Due to the smaller sample sizes (*N* < 30), the repeatabilities of the measurements were determined using a *t*-distribution with a 95% confidence interval. With the exception of the mobility size distribution measurements (*N* = 15), volume fraction measurements with the TDMA (*N* = 6) and TPM filter samples (*N* = 4), each measurement was repeated three times (i.e. *N* = 3) for each smoke source (tobacco or marijuana) and aerosol conditioning (nonstripped or stripped). The biased uncertainty of each measurement method was based on previous studies, specifically 3% uncertainty in particle mobility diameter by DMA classification^68^, 4.7% uncertainty in particle aerodynamic diameter by AAC classification^69^, 2.8% uncertainty in particle mass by CPMA classification^70^ and 10% uncertainty in particle concentration using a CPC^71^.

## Supplementary information

Supplementary information.

## Data Availability

The datasets generated during and/or analysed during the current study are available from the corresponding authors on reasonable request.
