# Supplementary material for: Comprehensive characterization of mainstream marijuana and tobacco smoke
Source: Sci Rep. 2020 Apr 28;10:7160. doi: 10.1038/s41598-020-63120-6 (PMC7188852; doi:10.1038/s41598-020-63120-6)
Supplement: Supplementary file 1 — Supplementary information. [file 41598_2020_63120_MOESM1_ESM.pdf]

Supplementary Information: Comprehensive characterization of mainstream marijuana and tobacco smoke

Brian M. Graves[1,+], Tyler J. Johnson[1,+], Robert T. Nishida[1,2,+,*], Ryan P. Dias[3], Benjamin Savareear[3], James J. Harynuk[3], Mohsen Kazemimanesh[2], Jason S. Olfert[2], and Adam M. Boies[1,*]

[1]Department of Engineering, University of Cambridge, Trumpington Street, Cambridge, CB2 1PZ, United Kingdom
[2]Department of Mechanical Engineering, University of Alberta, Edmonton, Alberta, T6G 1H9, Canada
[3]Department of Chemistry, University of Alberta, Edmonton, Alberta, T6G 2G2, Canada
[*]rnishida@ualberta.ca (Canada) or a.boies@eng.cam.ac.uk (UK)
[+]these authors contributed equally to this work

S1 Cigarette samples

The tobacco cigarettes smoked were 3R4F reference cigarettes (Kentucky Tobacco Research & Development Center, USA)[1]. Each 3R4F tobacco sample consists of 0.775 g of tobacco in a 57 mm long rod with a circumference of 24.8 mm[1]. A 27 mm long cellulose acetate filter forms the remainder of the 84 mm cigarette length. Smoking one 3R4F reference cigarette produces 0.73 mg of nicotine and 9.4 mg of tar[1]. The Kentucky reference tobacco products are designed to represent tobacco products of US consumers without any additional flavorings, with the 3R4F cigarette being more representative of the most popular tobacco cigarettes available in the US and having a stock-level of approximately 28 million[2]. Due to this wide-acceptance by researchers in this field, using a reference cigarette allows comparison of the results not just within this study, but between data collected in different studies, including those with different tobacco products[2].

The marijuana cigarettes smoked were ACES pre-rolled joints with milled sativa flower (Aurora Cannabis Inc., Edmonton, Canada). Neglecting the twisted paper end, each joint was approximately 90 mm long (6 mm longer than the 3R4F cigarette), including the cardboard tipping paper that was approximately 27 mm long (similar length as the 3R4F cellulose filter). Since no standard or reference joint exists, the ACES were chosen as the best alternative since (a) they contained milled Cannabis sativa flower, a common strain of marijuana, (b) they each contained 0.5 g of marijuana, a common mass for marijuana joints and the closest in size to the 3R4F tobacco cigarettes, (c) they were pre-rolled by machine to minimize variability between samples, (d) they are produced by the second largest cannabis company in the world by market capitalization (at the time of writing), (e) they are available for purchase direct to private consumers from provincial governments in Canada (from at least six provinces representing over 88% of the population of Canada[3]). Based on the Aurora product labelling, smoking one ACES joint produces 88.0 mg of THC (accounting for the decarboxylation of the THC-A during combustion) and 0 mg of CBD.

From the introduction of filtered cigarettes in the 1950s, their market share grew to more than 97% by the mid 1990s in the United States[4,5], United Kingdom[5] and Japan[6]. In 2016, filtered cigarettes accounted for 99.7% of the cigarettes purchased in the United States[4]. However, marijuana is smoked differently than cigarettes, usually without a filter[7–10], down to a smaller butt[9,11–13] and using longer/deeper inhalation techniques[9–12,14,15]. Of the thirteen provinces or territories in Canada, ten currently provide online government dispensaries, with nine dispensaries offering 244 pre-rolled products[*] at the time of writing. Eight of these nine government dispensaries were contacted[†], with three dispensaries confirming that all 61 pre-rolled products offered were nonfiltered (i.e. cardboard tipping paper). Based on online photos and descriptions of the other 183 pre-rolled products offered by the five other dispensaries, only two products were filtered. Therefore, approximately 99% of the pre-rolled marijuana joints currently offered across Canada are nonfiltered (i.e. cardboard tipping paper). To represent the most common consumption methods, this study compares the smoke produced from a filtered tobacco cigarette with that from a nonfiltered marijuana joint.

S2 Smoke generation and sampling

Smoke was produced from either tobacco cigarettes or marijuana joints using a Smoking Cycle Simulator machine (SCS; Cambustion Ltd., Cambridge, UK) as shown in Fig. S1. The SCS controlled the inhalation pattern during smoking using the

[*]This product total does not account for the same product being sold in different provinces/territories
[†]Currently, Nova Scotia requires an in-person identification validation before providing access to the online dispensary

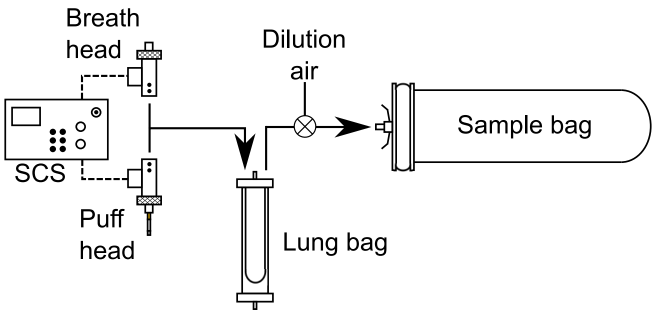

Figure S1. Experimental setup for smoking procedure

Health Canada Intense (HCI) puffing parameters (55 mL puff of 2 s duration, every 30 s, for 8 puffs)[16]. HCI was selected as it is a compromise between the ISO standard[17] puff routine (35 mL puff of 2 s duration, every 60 s, for 8 puffs) commonly used for tobacco cigarette smoking and the more aggressive routine (70 mL puff of 2 s duration, every 30 s, for 8 puffs) developed by Moir et al.[14] to represent the longer/deeper inhalation techniques used during marijuana joint smoking[9–12, 14, 15].

For aerosol characterization, mainstream smoke was sampled from the cigarette or joint through the heated puff head into a cylindrical lung bag (6.4 cm diameter and 35 cm length). Immediately prior to starting the puff routine, the lung bag was pre-filled using the heated breath head with 300 mL of HEPA-filtered air to act as a buffer volume during smoke generation. To limit smoke particle coagulation during the puff routine, 297 mL of HEPA-filtered dilution air was also added to the lung bag immediately prior to each 55 mL puff. The 355 mL volume (i.e. combined smoke and dilution air volume added) present in the lung bag after each puff was transferred through the heated breath head into a cylindrical sample bag (29.1 cm diameter and 100 cm length) between each puff. At the end of the puff routine, the remaining volume in the lung bag (i.e. last puff with dilution air and buffer air) was transferred to the sample bag. Prior to connecting to the SCS, the sample bag was manually pre-filled with 30 L of HEPA-filtered dilution air to provide sufficient volume for sampling with the particle characterization equipment. The lung and sample bag were both replaced between each cigarette or joint smoked, and were made of medium-duty (62.5 micron thick), polyethylene tubing (Kite Packing, Coventry, UK) with one end heat sealed. To avoid cross-contamination, the smoke generation apparatus was cleaned at the start of each day or when switching between smoking tobacco or marijuana samples.

For chemical analyses and total particulate matter (TPM) measurements, the non-vapor component of the smoke produced was sampled using quartz filters which were mounted in a stainless steel filter holder attached to the heated SCS puff head. This setup captured mainstream smoke directly from the mouth end of the cigarette or joint, therefore limiting sample losses and phase re-partitioning. During this filter loading, the stainless filter holder was cleaned between each cigarette or joint smoked and no lung and sample bag were required.

The filters (for chemical analyses and TPM measurements), cigarettes and joints were conditioned prior to sampling at a temperature of 22±1 °C and relative humidity of 60±3% for a minimum of 48 hours, in line with the ISO standard for tobacco conditioning and testing[18]. Flameless ignition was achieved using a coiled electric heater with a flat surface.

S3 Aerosol analyses

To assess some of the effects of the steady-state sampling on the smoke characteristics, the diffusion and settling losses of the particles during the aerosol analyses were estimated. The particle losses in each aerosol instrument was considered within its standard inversion procedure (i.e. SMPS correction following TSI[19] or AAC correction following Johnson et al.[20]) or accounted for based on a previous study (i.e. catalytic stripper correction following Dickau et al.[21]). The other aerosol measurements (i.e. particle mass, effective density and semi-volatile fraction) did not require corrections for particle losses in the DMA, CPMA or catalytic stripper as the results only depended on the location of the measurement peak in the mobility or mass domain, not its amplitude.

The particle losses in the sample bag were estimated following a similar approach as Johnson et al.[22], which is based on the aerosol theory summarized by Hinds[23]. The diffusion and losses in the sample bag were found to be insignificant to the lognormal parameters ($<$ 5.5% change) of the smoke size distributions. As expected, these corrections slightly increased the amplitudes of both the lower (i.e. diffusion losses) and upper (i.e. settling losses) extremities of the size distributions. As a result, the GSD and N for both the mobility and aerodynamic size distributions increased slightly, between 0.3-1.0% and 2.2-3.7%, respectively, for all but one smoke sample. The nonstripped marijuana smoke, with its larger particles, had slightly higher settling losses in the sample bag with the GSD and N of both the mobility and aerodynamic size distributions increasing

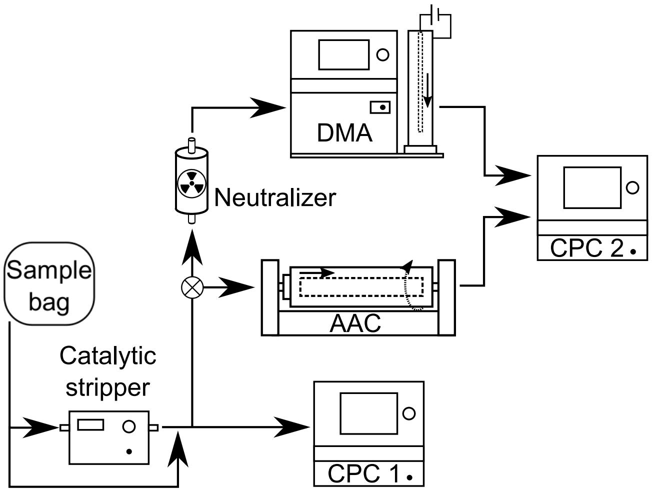

Figure S2. Experimental setup for measurement of either mobility size or aerodynamic size distributions of smoke particles.

between 1.1-1.3% and 4.4-5.5%, respectively. Due to these loss corrections being relatively symmetric, the CMDs of both the mobility and aerodynamic size distributions remained the same (< 2% change) for all of the smoke samples.

Furthermore, these changes in the size distribution are likely overestimated due to the loss estimates being conservative. The losses were estimated assuming the particle concentration remained constant in the sample bag and that the aerosol sample remained in the bag for 15 minutes before being sampled. In reality, the particle concentration in the bag decreases over time due to particle coagulation (as summarized in the Discussion section of the main text) and a portion of particles were sampled immediately after the puff routine finished. To further limit particle losses, the SMPS voltage was scanned upwards to measure the smallest particles first (i.e. where diffusion losses increase), and the AAC speed was stepped upwards to measure the largest particles first (i.e. where settling losses increase). These negligible losses of particles also agree with the insignificant diffusion and settling losses estimated by Johnson et al.[22] for nonstripped tobacco smoke in a 10 L sample bag. Since these loss estimates are conservative, are based on simplifying assumptions and are negligible relative to the other uncertainties of the measurements (as summarized in the Statistical Analysis section of the main-text), these loss corrections were not applied to the results presented in this study.

S3.1 Measurement of aerosol size distributions

The setup used to measure the particle size distributions is shown in Fig. S2. An aerosol sample flow was drawn from the sample bag at 0.6 L min^{-1}. This sample flow was first passed through a catalytic stripper (Catalytic Instruments GmbH & Co.KG, Rosenheim, Germany: Model CS015) operated with an internal gas temperature of 350°C (i.e. hot condition) or 21°C (i.e. cold condition). Under the hot catalytic stripper condition, the semi-volatile component of the aerosol was removed[24], whereas in the cold catalytic stripper, the semi-volatile component remained. The difference in particle losses within the catalytic stripper between the hot and cold operating conditions were corrected for following Dickau et al.[21].

The aerosol sample flow was then split, with 0.3 L min^{-1} drawn to a condensation particle counter (CPC), labelled as CPC 1 in Fig. S2. The CPC quantifies the particle number concentration of the aerosol by condensing butanol on the particles to generate droplets (several microns in diameter) large enough to be detected optically[25]. Due to this operating principle with the consistent working fluid, the CPC measurements are insensitive to the refractive index of the particles. The remaining 0.3 L min^{-1} sample flow was used to measure either the aerodynamic or mobility particle size distribution.

To measure the aerodynamic size distributions of the aerosol samples, an aerodynamic aerosol classifier (AAC; Cambustion Ltd.) and a CPC were used[20]. The AAC uses a centrifugal force to move particles that are in-turn resisted by their drag force in the carrier gas. Only particles with a narrow range of aerodynamic (-equivalent) diameters follow the correct trajectory and pass through the AAC[26]. This aerodynamic diameter setpoint can be set anywhere between 25 nm and 6.8 μm by varying the classifier speed and gas flows of the AAC[27]. At the flow rates used in this study, 0.3 and 3 L min^{-1} for the sample and sheath flow[‡], respectively, the classification range of the AAC was approximately 30 nm to 2.86 μm. By measuring the classified particle concentrations using a CPC (labelled CPC 2 in Fig. S2) as the AAC setpoint is stepped, the aerodynamic size distribution of an aerosol can be quantified[20]. These aerodynamic size distribution measurements were repeated three times, each from a new sample bag, and averaged to generate the size distribution parameters reported in Fig. 1a and in-text.

[‡]These classifier flow rates correspond to a classification resolution of 10.

Both CPCs shown in Fig. S2 and used in the other experimental setups for the aerosol portion of this study were the same model (TSI Inc., Shoreview, MN, USA: Model 3776) and detect particles with diameters from 2.5 nm to 3 μm[28]. Since the particle number concentrations in the sample bag decreased over time and the AAC scans were approximately 15 mins long, the particle number concentrations upstream of the AAC (measured by CPC 1) were used to normalize those downstream of the AAC (measured by CPC 2). This correction method was also utilized by Johnson et al.[22] for effective density measurements of aged tobacco smoke particles.

To measure the mobility size distributions of the aerosol samples, a Scanning Mobility Particle Sizer was used (SMPS; TSI Inc.: 3077 Bipolar charge conditioner, 3080 Electrostatic Classifier, 3081 Differential Mobility Analyzer [DMA], 3776 Condensation Particle Counter [CPC]). A bipolar charge conditioner was placed upstream of the DMA to charge the aerosol to a known equilibrium distribution of electrical charges. The DMA uses an electrostatic force to move the charged particles that are in-turn resisted by their drag force in the carrier gas. Only particles with a narrow range of mobility (-equivalent) diameters follow the correct trajectory and pass through the DMA[29]. This mobility diameter setpoint[§] can be set anywhere between 10 nm and 1 μm by varying the classifier voltage and gas flows of the DMA[19]. At the flow rates used in this study, 0.3 and 3 L min^{-1} for the sample and sheath flow[‡], respectively, the classification range of the DMA operating within an SMPS over a 120 second scan was approximately 15 to 685 nm.

By measuring the classified particle concentrations using a CPC (labelled CPC 2 in Fig. S2) as the DMA setpoint is scanned, the mobility size distribution of an aerosol can be quantified[30]. This measured distribution was corrected for multiply-charged particles following He & Dhaniyala[31]. The five consecutive mobility size distribution measurements (i.e. consecutive three minute scans started after the puff routine finished) were repeated three times, each from a new sample bag, and averaged to generate the size distribution parameters reported in Fig. 1b and in-text. The mobility scan shown in Fig. 1b is an average of all fifteen SMPS scans, accounting for the change in the smoke particle concentration over time by scaling the entire distribution by its total particle concentration relative to that from the first scan.

Both the aerodynamic and mobility particle size distributions were characterized by a log-normal distribution using a least-squares regression to determine the fitted count median diameter (CMD), geometric standard deviation (GSD), and total number concentration. These measurements focused on the measured CMD and GSD of the smoke particles, as the total particle number concentrations quantified by these methods were limited due to the transient nature of the smoke particles as discussed in the next section.

S3.2 Measurement of total number concentration

The particle number concentration of each aerosol sample was directly measured as a function of time using CPC 1 as labelled in Fig. S2. The particle number concentration was also indirectly measured by integrating the area under the aerodynamic or mobility size distributions.

The measured particle number concentration decreased over time as shown in Fig. S3 and Fig. S4. These concentrations are corrected for the sample dilution factor of 75 which accounts for the 30 L of dilution air in the sample bag, and 297 mL of dilution air per puff and 300 mL of buffer dilution air added into the lung bag.

Fig. S4 shows the mobility size distributions of the nonstripped tobacco smoke particles measured by the SMPS as a function of time. This measurement demonstrates that the mobility CMD remained relatively constant (within 1.4% of the average CMD of nonstripped tobacco smoke) between the 5 consecutive 3 minute scans (i.e. 15 minute total measurement time). The change in the mobility CMD over the 5 consecutive mobility scans from either smoke source and condition (nonstripped vs stripped) varied by less than 5.4% over a similar period.

All three measurement methods of particle concentration varied due to the transient nature and high concentration of the smoke particles. The reported CPC 1 measurements include a coincidence correction as the measured particle concentrations were greater than 3×10^5 particles cm^{-3}, a threshold above which the CPC has reduced measurement accuracy[28]. Furthermore, CPC 1 was measuring at the maximum of its range (1×10^6 particles cm^{-3}) for the first 0 to 15 seconds of sampling depending on the smoke source and its conditioning (nonstripped or stripped). It should be noted that these conditions did not occur in CPC 2 as the particle concentrations were below the CPC coincidence correction threshold after classification by the AAC, DMA, or CPMA. The number concentrations determined from the mobility measurements are sensitive to the SMPS multiple-charge correction[20,32]. Furthermore, the common practice for characterizing steady-state aerosols (as alluded to in Annex G of BS ISO 15900[33]) is to use a SMPS to measure the mobility size distribution and in parallel use a CPC to measure the total particle number concentration since charge corrections are not required for a CPC. While the aerodynamic measurements do not require a multiple charge correction[20], its long scan time ($\approx$15 minutes) of a transient aerosol likely had the greatest effect on the area under the measured aerodynamic size distribution. This inference is supported by the stability of the mobility CMD between consecutive SMPS measurements. However, despite these additional sources of concentration measurement errors, the particle

[§]Assuming the particles are singly charged.

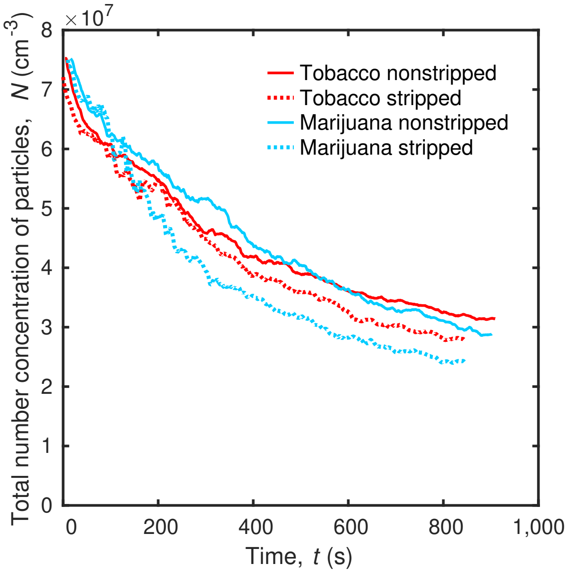

Figure S3. Change in particle number concentration of nonstripped tobacco and marijuana smoke over time.

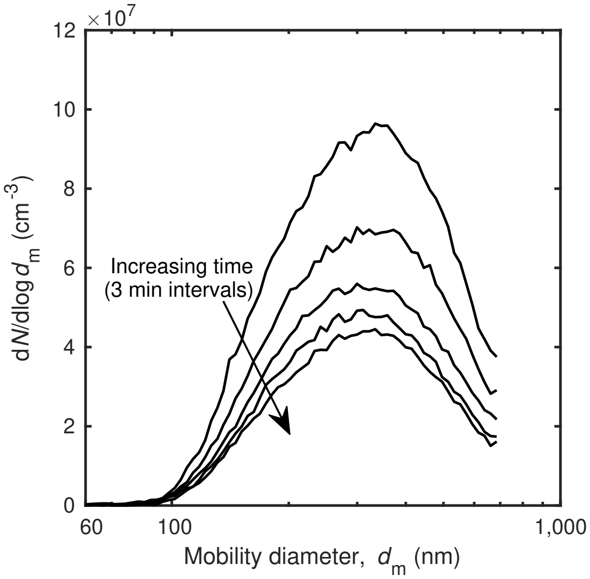

Figure S4. Change in mobility size distribution of nonstripped tobacco smoke over time. Total number concentration reduces over time and mean diameter remains relatively constant.

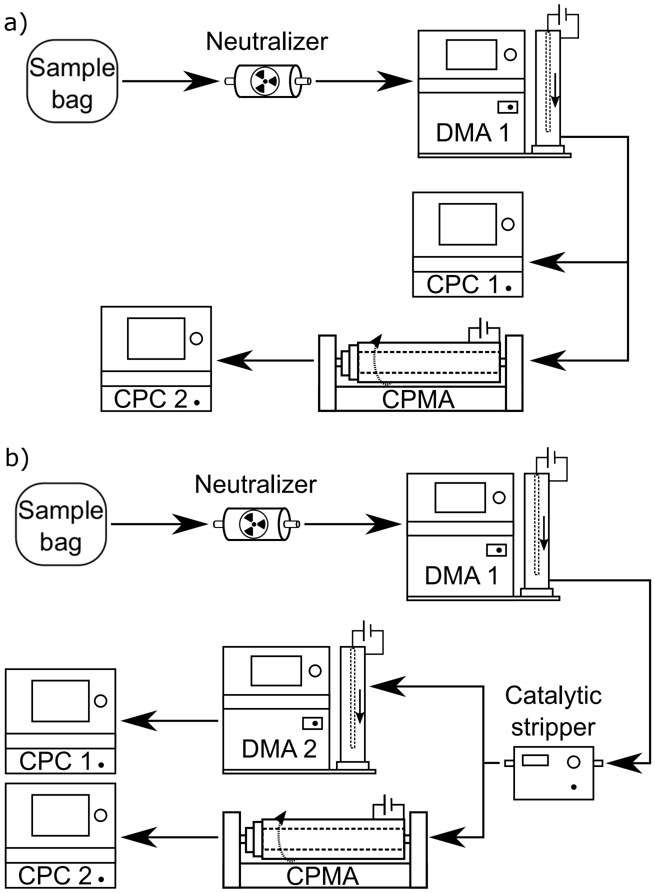

Figure S5. Experimental setups for measurement of size-selected effective density and size-selected semi-volatile volume fraction of smoke particles using (a) nonstripped and (b) stripped particles.

concentration ratio between marijuana and tobacco smoke from all three measurement methods agreed within 20% for both nonstripped and stripped aged smoke samples as shown in Fig. 1c and in-text.

S3.3 Measurement of effective density

Effective density of nonstripped smoke particles as a function of particle mobility diameter was measured using the setup shown in Fig. S5a. Similar to the experimental setups for aerosol size distribution measurements, a sample flow rate of 0.6 L min^{-1} was used. However, in this case, the smoke particles were first classified by mobility using DMA 1 at a constant diameter setpoint prior to a second aerosol classifier. Similar to the previous setups, the sample flow was split, with 0.3 L min^{-1} drawn by CPC 1 to measure the total number concentration of the classified particles (i.e. downstream of DMA 1), which were monodispersed in electrical mobility. The remaining 0.3 L min^{-1} classified sample flow was passed through a centrifugal particle mass analyzer (CPMA) to classify the particles by their mass-to-charge ratio using opposing centrifugal and electrostatic forces generating by charged, rotating concentric cylinders[34]. This mass-to-charge setpoint[§] can be set anywhere between 0.2 ag and 1050 fg by varying the classifier speed and voltage of the CPMA[35]. At the sample flow of 0.3 L min^{-1} and the classification resolution of 20 used in this study, the classification range of the CPMA was approximately 3.8 ag to 296 fg. The CPMA was operated at twice the classification resolution of the AAC or DMA (i.e. 20 vs 10) to further distinguish the charge states of the particles classified by the upstream DMA.

By measuring the classified particle concentrations using a CPC (labelled CPC 2 in Fig. S5a) as the CPMA setpoint is stepped, the mass-to-charge distribution of an aerosol can be quantified[36,37]. Since the charge states of the particles are known from the upstream neutralizer and DMA, the particle mass classified by the CPMA at the given DMA setpoint can be determined. Using the measured particle mass and mobility diameter, the effective density of the particles can be calculated following Eqn. 1 of main-text.

The effective densities of stripped particles were measured using the setup shown in Fig. S5b. The 0.6 L min^{-1} sample classified by DMA 1, which was monodispersed in particle mobility, was passed through a catalytic stripper at 350°C. The

effective density of the particles was measured by using the two downstream classifiers, DMA 2 and the CPMA, in parallel to measure the mobility diameter and mass of the stripped particles, respectively. Each downstream classifier was operated in tandem with a CPC operating at at 0.3 L min^{-1}. Due to this tandem classifier arrangement, DMA 2 was not operated in scanning mode like an SMPS to avoid introducing additional errors into its mobility measurements. These errors are caused by the upstream DMA (i.e. DMA 1) producing a narrow mobility distribution, which invalidates the SMPS inversion assumption that the aerosol distribution is constant over the width of the downstream DMA (i.e. DMA 2) transfer function[38]. To avoid this issue, the downstream DMA was stepped, rather than scanned, and the tandem DMA inversion developed by Stolzenburg & McMurry[39] was applied. This tandem DMA inversion was also previously used to study the hygroscopic properties of tobacco smoke particles[40].

For both the nonstripped and stripped smoke samples, this process was repeated at various particle sizes (i.e. different DMA setpoint) to identify the relationship between particle mobility diameter and effective density as shown in Fig. 1d and in-text.

S3.4 Measurement of total particulate matter

The total particulate matter (TPM) produced by smoking a tobacco cigarette or marijuana joint was measured by collecting fresh smoke (i.e. without dilution or aging) on a quartz filter directly downstream of the cigarette or joint. The quartz filters (47 mm Type A/E Glass Fiber Filter from Pall Life Sciences [P/N 61631]) were weighed before and after loading using an ultra-microbalance (UMX2; Mettler Toledo, Columbus, Ohio, USA). To avoid the TPM filters becoming overloaded and causing the puff routine to deviate significantly, only six of the eight puffs of the HCI smoking routine were used during the TPM filter loading for both the tobacco and marijuana samples. A TPM sample was deemed acceptable if the total puff volume was within 10% of the 330 cm^3 target. The total puff volumes deviated by -0.6% to -3.2% for the TPM filters loaded with tobacco smoke, and by 0.2% to -8.7% for those loaded with marijuana smoke. These deviations are on the same order of magnitude as the uncertainty of other studies, such as the 5% to 7% confidence interval calculated from the data of Moir et al.[14], which had a larger TPM sample size of 30 repeats.

Immediately after loading the filters were placed on the ultra-microbalance, however each filter's mass would continue to decrease for tens of minutes. This decrease was small relative to the total TPM (less than 3.6%) and is likely due to the volatility of the TPM. This observation at room temperature supports the conclusion that the particles from either smoke source have a highly volatile component. The TPM filters were also conditioned before and after loading, however the change in total mass due to conditioning after loading was insignificant (less than 0.6%). Over the 6 HCI puffs, the marijuana joint produced 27 ($\pm$4) mg of TPM, while the smoking the tobacco cigarette produced 7.8 ($\pm$0.9) mg of TPM. The reported TPM measurement limits represent the 95% confidence of the measurement repeatability assuming a t-distribution. These results agree with the results of Ingebrethsen et al.[41], which found smoking the same 3R4F tobacco cigarette following the same HCI puff routine produced 1.0$\pm$0.3 mg and 1.9$\pm$0.3 mg of TPM for puff 2 and puff 5, respectively, thus agreeing with the 1.3$\pm$0.3 mg average TPM per tobacco cigarette puff currently measured. This per puff average is only a high-level comparison as it neglects that the TPM produced changes as a function of rod length or with each puff over a smoking routine.

Based on the TPM measurements and associated confidence intervals determined in the current study, smoking a marijuana joint produces roughly 3.4 ($\pm$0.6) times more TPM than a tobacco cigarette following the same six puff routine. This result qualitatively agrees with our observation that more than six HCI puffs from the marijuana joint would overload the TPM filter causing significant puff deviations, while no such overloading and puff deviations were observed for TPM samples loaded with up to eight HCI puffs from the tobacco cigarette. However, this 3.4 ratio is contrary to the results of Moir et al.[14], which found tobacco cigarettes and marijuana joints produce approximately the same amount of TPM per puff following either the ISO (35 ml puff over 2 seconds every 60 seconds) or extreme (70 ml puff over 2 seconds every 30 seconds) smoking routines. This difference between the studies could be due the different puffs routines used, and that the tobacco cigarettes studied by Moir et al.[14] were nonfiltered as filtered cigarettes are known to reduce the TPM produced from smoking tobacco[5]. The inference is supported by another study[42] which found on average that mainstream smoke from a marijuana joint yields on average 2.2 times greater TPM than mainstream smoke from a filter-tipped tobacco cigarette.

S3.5 Measurement of semi-volatile mass and volume fractions

The semi-volatile mass and volume fractions at a given aerosol particle size were measured using the setup shown in Fig. S5a and S5b. The semi-volatile mass fraction at a given particle size was measured by comparing the masses of nonstripped and stripped particles measured by the CPMA at a given DMA setpoint, in this case 470 nm mobility diameter. Specifically, the count median mass (CMM) measured by the CPMA in Fig. S5a is compared with the CMM measured by the CPMA in Fig. S5b. Each CMM was determined by fitting a log-normal distribution to each charge peak of the CPMA scan using a least-squares regression.

The semi-volatile volume fraction at a given particle size was measured by comparing the mobility-equivalent volume of nonstripped and stripped particles measured by DMA 2 at a given DMA 1 setpoint as shown in Fig. S5b. Note that DMA 2 was operated in stepping mode for the reasons described in Section S3.3. Specifically, the TDMA inversion median diameter of the

stripped particles measured by DMA 2 is compared with the nonstripped particle size selected by DMA 1 (i.e. 470 nm mobility diameter setpoint).

The semi-volatile fractions for the polydispersed size distributions were calculated using the mass and volume concentrations of the aerosols, rather than the individual particle mass and volume. The volume and mass concentrations were estimated using the Hatch-Choate equations[23] and the lognormal distributions fitted to the measured mobility size distributions (as described in Sections S3.1). The fitted lognormal distribution was used to account for particles smaller or larger than the SMPS measurement range (14.6 to 685.4 nm). It is clearly shown in the mobility size distributions of Fig. 1b that particles larger than the SMPS measurement range are present. The mass concentration estimate also utilized the effective density of the particles as described in Section S3.3.

S4 Chemical analyses

Volatile compounds from particulates captured in pre-fired quartz filters were sampled by headspace solid-phase microextraction (HS-SPME) followed by comprehensive two-dimensional gas chromatography with a time-of-flight mass spectrometric detector (GC×GC-TOFMS). The pre-fired quartz filters (47 mm PallFlex Tissuquartz™ 2500 QAT-UP filters from Pall Life Sciences [P/N 7202]) utilized for the chemical samples were different than the TPM filters and appeared to generate a lower pressure drop during loading. As a result, the chemical sample filters did not cause the puffs to deviate late in the smoking routine, and thus could be consistently loaded using all eight puffs for both the tobacco and marijuana samples.

S4.1 HS-SPME-GCxGC-TOFMS

The GC×GC-TOFMS system consisted of an Agilent 7890 (Agilent Technologies, Palo Alto, CA, USA) gas chromatograph and a Pegasus 4D TOFMS (LECO, St. Joseph, MI, USA) with quad jet liquid nitrogen-cooled thermal modulator. The first dimension (^{1}D) column was a low-polarity 5% phenyl / 95% polydimehthylsiloxane-type phase (Rtx-5MS; 60 m × 0.25 mm i.d.; 0.25 μm film thickness) connected by means of a SilTite μ-Union (Trajan Scientific and Medical, Victoria, Australia) to a second dimension (^{2}D) mid-polarity trifluoropropylmethyl polysiloxane-type phase (Rxi-200; 1.6 m × 0.25 mm i.d.; 0.25 μm film thickness). Both columns were from Restek Corporation (Restek Corp., Bellefonte, PA, USA). The ^{2}D column was installed in a separate oven located inside the main GC oven. The system was equipped with a Gerstel MultiPurpose Sampler (MPS 2XL) with SPME option for procedural automation. The carrier gas was helium at a corrected constant flow rate of 2 mL min^{-1} and the injector operated in splitless mode. The main oven temperature program was 50°C (5 min hold), a ramp of 5°C min^{-1} to 300°C (1 min hold). The secondary oven was programmed with a constant +10°C offset relative to the primary oven. The modulation period was 2.0 s (0.3 s hot pulse and 0.7 s cold pulse time) with a +15°C offset relative to the secondary oven. Mass spectra were acquired in the range m/z 40–500 at 200 spectra s^{-1}. The ion source temperature was set at 200°C and the transfer line temperature was set at 250°C. The detector voltage was run at an offset of -200 V relative to the tuning potential and the ionization electron energy (EI source) was set at 70 eV. Samples were acquired using LECO ChromaTOF software version 4.72.0.0

After smoking, filter pads were immediately placed in separate 20 mL headspace vials and sealed with magnetic crimp-top caps. Samples were stored in a refrigerator (≈4°C) prior to analysis. The filter pads used for this chemical sampling were pre-fired in a muffle furnace at 750°C for 4 hours. Cigarettes/joints, and filter pads were conditioned for at least 48 h at 60±3% relative air humidity and 22±1°C prior to use[18]. Four different SPME fibre coatings were examined. The fibre coatings comprised a tri-mode (50/30 μm DVB/CAR/PDMS; divinylbenzene/carboxen on polydimethylsiloxane) fibre, a mixed-mode (65 μm PDMS/DVB; polydimethylsiloxane/divinylbenzene) fibre, a PDMS (100 μm polydimethylsiloxane) fibre, and a PA (85 μm polyacrylate) fibre, all purchased from Millipore Sigma (USA). All fibres were used to perform extractions from the headspace over the filter pads using the following conditions: an incubation time of 3 min at 70°C and an extraction time of 15 min at 70°C. The inlet temperature was maintained at 250°C during fibre desorption (2 min). Fibres were initially conditioned according to the manufacturer's guidelines.

Data were processed using LECO ChromaTOF software version 4.71.0.0 with the following parameters. The expected peak width settings in the ^{1}D and ^{2}D were 12 s and 0.1 s, respectively. Peaks were detected from the raw chromatogram using a minimum signal-to-noise (S/N) ratio value of 100 with a minimum sub-peak S/N of 6. The minimum match required to combine peaks was 750. Identities of peaks were tentatively assigned on the basis of linear temperature-programmed retention indices (LTPRIs) for C5-C30 (LTPRI window ±10) and mass spectral similarity match (>750) against library spectra. Mass-spectral library searches were performed against the NIST/EPA/NIH Mass Spectral Library (NIST 17) and Wiley Registry of Mass Spectral Data (9th edition). Interactive LTPRI filters (±10) were performed by using the NIST/EPA/NIH Mass Spectral Library (NIST 17 version 2.3) as well as internet-based RI collections (i.e. PubChem) databases. Unless otherwise stated, all experiments were conducted with the aforementioned parameters. Detailed HS-SPME-GC×GC-TOFMS acquisition and data processing methods are provided at the end of the SI (SPME-GCxGC-TOFMS methods).

S4.2 SPME fibre selection

Uptake of analytes in SPME is highly dependent on the chemistry of the fibre used to perform the extraction and in this study, four different SPME fibres were investigated for their utility in profiling volatiles: polydimethylsiloxane (PDMS), polyacrylate (PA), Divinylbenzene(DVB)/PDMS, and DVB/Carboxen(CAR)/PDMS. A comprehensive profile of the compounds in the smoke is desired, while ensuring good responses for compounds that are expected to be of interest, namely the terpenes and cannabinoids. During initial development of the GC$\times$GC method, the PDMS fibre was abandoned as it gave poor recoveries for most compounds in tobacco and marijuana smoke. Typical chromatograms for both tobacco and marijuana smoke with the various fibre chemistries tested are included in Fig. S6.

Raw chromatograms consisted of several thousand peaks for each sample with the processing method employed. In curating the peak tables, unwanted peaks such as column bleed and extra hits from tailing peaks were removed. When we considered the total number of detected compounds, the PA fibre yielded 4329 and 1936 peaks for the particulate phase fraction of mainstream tobacco smoke and mainstream marijuana smoke, respectively. Similarly, using DVB/PDMS fibre 3680 peaks in the particulate phase fraction of mainstream tobacco smoke and 1845 peaks in the particulate phase fraction of mainstream marijuana smoke were detected. When the DVB/CAR/PDMS fibre was used, the total number of compounds detected increased to 4350 and 2575 for the particulate phase fraction of mainstream smoke from tobacco and marijuana, respectively. Although these values revealed the relative differences and complexity of the tobacco smoke and marijuana smoke, the DVB/CAR/PDMS fibre shows better extraction efficiency towards a larger number of analytes with diverse chemical functionality for both sample matrices. Hence this fibre type was selected for all the subsequent analyses.

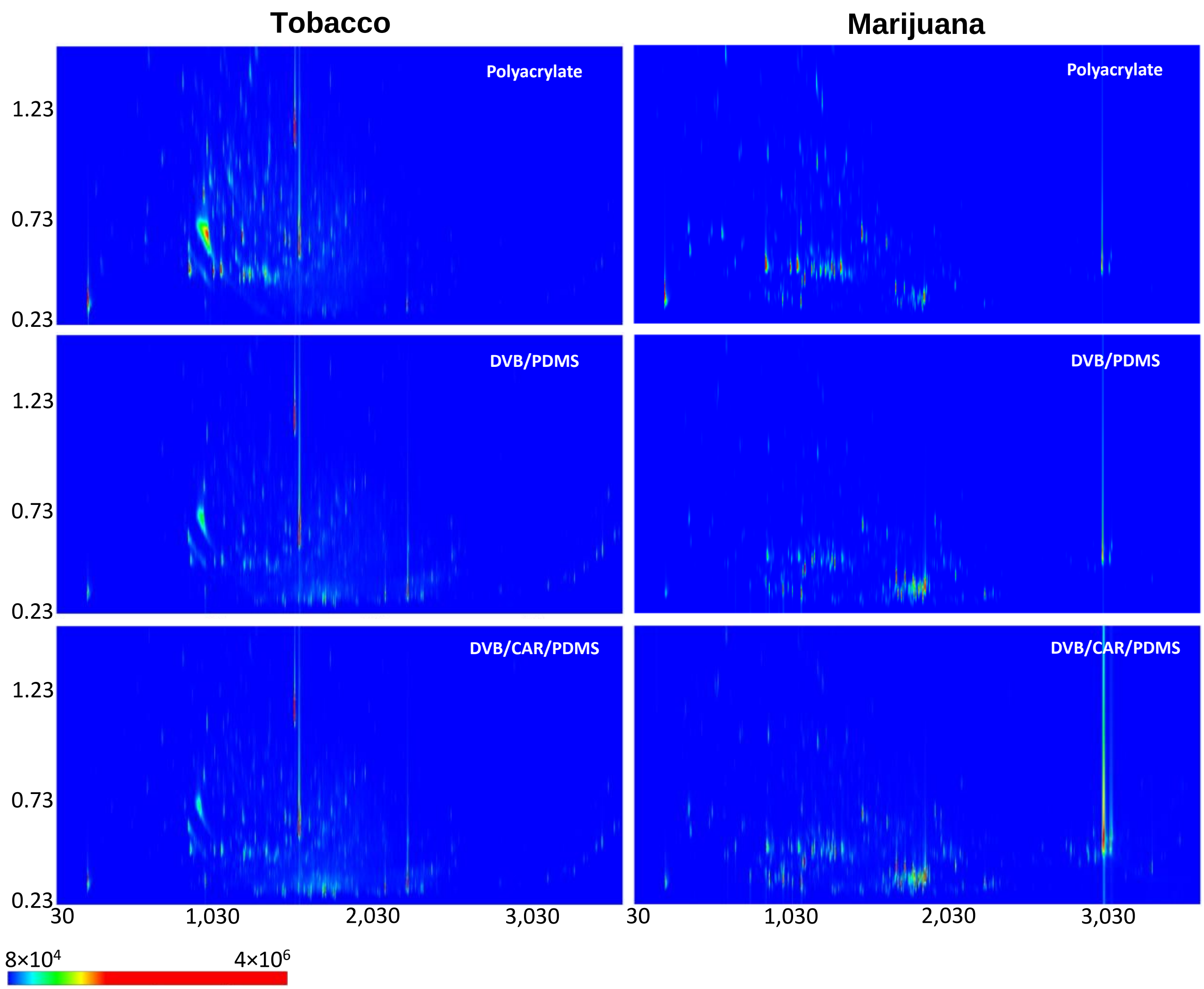

Figure S6. HS-SPME-GC×GC-TOFMS performed using three different fibres

Table S1. Summary table of identified peak classes and relative abundances (based on calculation of areas using unique ions) of compounds in mainstream marijuana and tobacco smoke on filter pads using HCI smoking regime

	Tobacco		Marijuana		Common		
Chemical Class	No. of Peaks	Total Peak Area (%)	No. of Peaks	Total Peak Area (%)	No. of Peaks	Tobacco Total Peak Area (%)	Marijuana Total Peak Area (%)
**Heterocycle**							
Furan	19	1.4	13	2.6	8	1.0	2.5
Pyran	9	0.6	ND	ND	NF	NF	NF
Pyrazine	16	0.8	12	2.1	9	0.7	1.8
Pyrazole	1	0.1	ND	ND	NF	NF	NF
Pyridine	46	7.1	18	2.8	12	1.4	2.5
Pyrimidine	1	trace	1	trace	NF	NF	NF
Pyrrole	17	1.1	17	2.8	9	0.5	2.2
Total	109	12	61	10	38	3.6	9.0
**Hydrocarbon**							
Alicyclic	15	0.6	24	1.1	NF	NF	NF
Alkane	19	2.8	24	1.7	17	2.8	1.6
Alkene	38	4.0	33	1.6	16	1.3	1.0
Alkyne	5	0.2	1	trace	NF	NF	NF
Diterpene	1	1.9	3	0.4	1	1.9	0.4
Monocyclic Aromatic	71	3.1	53	4.9	28	1.3	3.3
Monoterpene	7	0.6	24	5.6	6	0.6	3.0
Polycyclic Aromatic	82	4.9	34	2.1	26	2.9	2.0
Sesquiterpene	21	1.5	50	11	12	1.2	5.1
Triterpene	1	trace	ND	ND	NF	NF	NF
Total	260	18	246	28	106	12	16
**Nitrogenated**							
Amide	3	0.3	1	trace	NF	NF	NF
Amine	6	0.3	1	trace	NF	NF	NF
Amino Acid	2	trace	2	0.1	1	trace	trace
Aza-arene	30	2.7	9	2.7	8	2.2	2.7
Imide	2	trace	2	0.7	1	trace	trace
Lactam	1	trace	ND	ND	NF	NF	NF
Nitrile	21	1.4	26	4.8	10	1.0	3.6
Oxazole	2	trace	1	trace	1	trace	trace
Thiazole	1	trace	ND	ND	NF	NF	NF
Total	68	4.8	42	8.4	21	3.3	6.4
**Oxygenated**							
Alcohol	55	20	75	16	20	6.2	6.2
Aldehyde	16	0.2	3	trace	2	trace	trace
Anhydride	1	trace	ND	ND	NF	NF	NF
Cannabinoid	ND	ND	8	7.1	NF	NF	NF
Carboxylic Acid	2	3.2	2	2.0	1	3.1	2.0
Epoxide	4	0.2	7	0.7	NF	NF	NF
Ester	12	7.0	27	1.5	4	6.2	0.6
Ether	15	0.8	8	0.4	7	0.6	0.4
Ketone	119	10	49	5.1	29	3.4	4.3
Lactone	2	0.7	1	trace	NF	NF	NF
Peroxide	1	0.1	ND	ND	NF	NF	NF
Quinone	2	0.3	ND	ND	NF	NF	NF
Total	229	43	180	34	63	20	13
**Miscellaneous**							
Acetamide	ND	ND	1	trace	NF	NF	NF
Amphetamine	ND	ND	1	0.1	NF	NF	NF
Carbamate	ND	ND	1	trace	NF	NF	NF
Phosphonate	1	trace	ND	ND	NF	NF	NF
Thiocyanate	ND	ND	1	trace	NF	NF	NF
Trisulfide	1	trace	1	trace	1	trace	trace
Total	2	trace	5	trace	1	trace	trace
**Matrix Total**	**668**	**100**	**534**	**100**	**229**	**39**	**45**

Table S2. List of compounds from the particulate phase fraction of mainstream tobacco (TC) or marijuana (MJ) smoke detected by HS-SPME-GC×GC-TOFMS and a contract laboratory using their standard quantitative methods

Compound	Unit	RDL	TC 1	TC 2	MJ 1	MJ 2	GC×GC TOF Only	GC×GC TOF S/N (TC)	GC×GC TOF S/N (MJ)
delta-9-Tetrahydrocannabinol[+]	mg	0.05	<RDL	<RDL	20.0	18.6	-	ND	9,600
delta-8-Tetrahydrocannabinol[+]	mg	0.05	<RDL	<RDL	<RDL	<RDL	X	ND	2,500
Cannabidiol[+]	mg	0.05	<RDL	<RDL	<RDL	<RDL	X	ND	4,600
Cannabinol[+]	mg	0.05	<RDL	<RDL	<RDL	<RDL	X	ND	3,000
Cannabigerol[+]	mg	0.05	<RDL	<RDL	<RDL	<RDL	X	ND	500
Cannabichromene[+]	mg	0.05	<RDL	<RDL	<RDL	<RDL	X	ND	22,000
Nicotine*	μg	0.04	1040	1130	<RDL	<RDL	-	24,000	ND
Acenaphthene	μg	0.5	<RDL	<RDL	<RDL	<RDL	X	2,500	690
Acenaphthylene	μg	0.5	<RDL	<RDL	<RDL	<RDL	X	4,500	1,900
Anthracene	μg	0.5	<RDL	<RDL	<RDL	<RDL	X	670	870
Fluorene	μg	0.5	<RDL	<RDL	0.6	<RDL	-	8,200	2,000
Naphthalene, 1-methyl-	μg	0.5	<RDL	<RDL	1.3	1.0	-	19,000	7,600
Naphthalene, 2-methyl-	μg	0.5	<RDL	<RDL	1.8	1.3	-	16,000	10,000
Naphthalene	μg	0.5	<RDL	<RDL	2.3	1.7	-	36,000	29,000
Phenanthrene*	μg	0.5	<RDL	<RDL	<RDL	<RDL	X	2,600	ND
Pyrene[+]	μg	0.5	<RDL	<RDL	<RDL	<RDL	X	ND	300
Ethylbenzene	mg/kg	5.0	<RDL	<RDL	10.8	<RDL	-	660	8,700
Styrene	mg/kg	5.0	<RDL	<RDL	53.9	13.5	-	1,200	15,000
Toluene	mg/kg	5.0	<RDL	<RDL	6.4	<RDL	-	ND	ND
m-Xylene	mg/kg	5.0	<RDL	<RDL	*a*	<RDL	X	1,700	1,300
o-Xylene	mg/kg	5.0	<RDL	<RDL	*a*	<RDL	X	300	200
p-Xylene	mg/kg	5.0	<RDL	<RDL	*a*	<RDL	X	1,000	4,100
Formaldehyde[b]	μg	2.5	14.1	14.5	17.3	14.8	-	ND	ND
Acetaldehyde (cis)[c]	μg	2.5	12.3	12.6	22.7	21.1	-	ND	ND
Acetaldehyde (trans)[c]	μg	2.5	12.0	12.2	22.1	20.4	-	ND	ND

RDL is the reported detection limit of the analytical method (one-dimensional GC-MS or LC-UV) used by the contract laboratory performing quantitative analysis. Quantified compounds in two replicate samples employ the same units stated with RDL values.

ND = not detected by GC×GC-TOFMS

*Detected in tobacco only

[+]Detected in marijuana only

[a]Reported as "xylenes" at 25.5 mg/kg by external laboratory; individual quantification was not provided

[b]Formaldehyde is a Group 1 carcinogen detected by external laboratory only.

[c]Acetaldehyde is a Group 2B carcinogen (Group 1 carcinogen when associated with the consumption of alcoholic beverages) detected by external laboratory only.

Table S3. Tentatively identified compounds from the particulate phase fraction of mainstream tobacco smoke. G1 = Group 1 carcinogen, G2A = Group 2A carcinogen, G2B = Group 2B carcinogen, G3 = Group 3 carcinogen, M = Mutagen, T = Teratogen, X = Toxic by other mechanisms, ND = No risk data available or risks mitigated by proper protective equipment.

Tentative Identification	CAS	Peak Area	Health Effects	Class	Subclass	^1t_R (s)	^2t_R (s)	Quant Mass	Exp. RI*	Lib. RI*	Reverse†	Similarity‡
Acetic acid	64-19-7	19571743	-	Oxy	carboxylic acid	224	0.37	60	650	649	983	981
2-Propanone, 1-hydroxy-	116-09-6	4363457	M	Oxy	ketone	278	0.86	43	683	688	959	948
2,3-Pentanedione	600-14-6	127492	-	Oxy	ketone	306	0.78	57	700	698	963	815
2-Butanone, 3-hydroxy-	513-86-0	349470	-	Oxy	ketone	326	0.965	45	713	713	928	846
Butanenitrile, 3-methyl-	625-28-5	196831	X	Nit	nitrile	354	1.14	43	730	730	869	818
Pyridine	110-86-1	2058995	G2B	Het	pyridine	376	0.68	79	743	746	936	937
(Dimethylamino)-acetonitrile	926-64-7	130480	X	Nit	nitrile	380	1.04	83	746	NA	936	791
1H-pyrrole	109-97-7	249200	M	Het	pyrrole	388	0.58	67	750	755	968	833
Cyclopentanone	120-92-3	704948	-	Oxy	ketone	454	1.05	55	791	791	980	951
Pyridine, 2-methyl-	109-06-8	1027226	M	Het	pyridine	504	0.61	93	816	816	953	937
2-Methylpyrazine	109-08-0	1098300	M	Het	pyrazine	522	0.66	94	825	831	955	942
2-Cyclopenten-1-one	930-30-3	1210377	-	Oxy	ketone	540	1.24	82	834	834	951	934
Cyclopentanone, 2-methyl-	1120-72-5	376556	-	Oxy	ketone	556	0.96	42	841	843	944	922
Pentanenitrile, 4-methyl-	542-54-1	502268	-	Nit	nitrile	558	1.28	55	842	843	953	904
Cyclopentanone, 3-methyl-	1757-42-2	157752	-	Oxy	ketone	574	1.08	69	850	851	933	841
Ethylbenzene	100-41-4	158822	G2B	HC	monocyclic aromatic HC	600	0.46	91	862	862	912	881
2-Furanmethanol	98-00-0	463642	G2B	Het	furan	600	0.64	98	862	859	932	830
Pyridine, 3-methyl-	108-99-6	2815096	M	Het	pyridine	600	0.77	93	862	861	953	953
2-Propanone, 1-(acetyloxy)-	592-20-1	4938899	-	Oxy	ester	610	1.49	43	867	867	964	944
(2E,4E)-2,4-Hexadienal	142-83-6	279067	G2B	Oxy	aldehyde	614	1.12	81	868	876	860	843
m-Xylene	108-38-3	484063	G3	HC	monocyclic aromatic HC	620	0.47	91	871	871	942	933

Continued on next page

Table S3. Tentatively identified compounds from the particulate phase fraction of mainstream tobacco smoke (Continued)

Tentative Identification	CAS	Peak Area	Health Effects	Class	Subclass	^1t_R (s)	^2t_R (s)	Quant Mass	Exp. RI*	Lib. RI*	Reverse†	Similarity‡
o-Xylene	95-47-6	118989	G3	HC	monocyclic aromatic HC	624	0.46	91	873	874	928	802
Pyridine, 2,6-dimethyl-	108-48-5	220664	M	Het	pyridine	644	0.5	107	882	880	961	903
Styrene	100-42-5	411657	G2A	HC	monocyclic aromatic HC	668	0.5	104	894	894	958	920
p-Xylene	106-42-3	207834	G3	HC	monocyclic aromatic HC	670	0.49	91	895	894	942	918
Cyclohexanone	108-94-1	154507	G3	Oxy	ketone	674	1.04	55	897	895	911	812
Pyridine, 2-ethyl-	100-71-0	410300	-	Het	pyridine	690	0.55	106	904	904	928	918
2-Cyclopenten-1-one, 2-methyl-	1120-73-6	3068641	-	Oxy	ketone	694	1.01	67	906	907	971	970
Pyrazine, 2,6-dimethyl-	1192-62-7	1507214	M	Het	pyrazine	710	0.6	108	913	912	948	943
2-acetylfuran	108-50-9	985256	M	Het	furan	710	1.04	95	913	914	923	892
Pyrazine, ethyl-	13925-00-3	200279	M	Het	pyrazine	720	0.61	107	918	917	928	855
Pyrazine, 2,3-dimethyl-	5910-89-4	388943	X	Het	pyrazine	724	0.61	67	920	920	873	839
Pyridine, 2,4-dimethyl-	108-47-4	449871	M	Het	pyridine	752	0.62	106	932	932	955	925
2-Cyclohex-1-one	930-68-7	463075	M	Oxy	ketone	758	1.14	68	935	939	924	863
Pyridine, 2,5-dimethyl-	589-93-5	181366	-	Het	pyridine	760	0.615	106	936	946	834	791
Pyridine, 2,3-dimethyl-	583-61-9	232018	M	Het	pyridine	780	0.64	106	945	945	930	886
Benzene, 2-propenyl-	300-57-2	36610	X	HC	monocyclic aromatic HC	786	0.45	117	948	947	925	832
2-Heptanone, 6-methyl-	928-68-7	259963	-	Oxy	ketone	802	0.83	43	955	956	897	777
Benzene, propyl-	103-65-1	266447	-	HC	monocyclic aromatic HC	804	0.44	91	956	956	954	899
Cyclopentanone, 3,4-bis(methylene)-	27646-73-7	165346	-	Oxy	ketone	810	0.88	79	959	949	880	773

Continued on next page

Table S3. Tentatively identified compounds from the particulate phase fraction of mainstream tobacco smoke (Continued)

Tentative Identification	CAS	Peak Area	Health Effects	Class	Subclass	$^{1}t_R$ (s)	$^{2}t_R$ (s)	Quant Mass	Exp. RI*	Lib. RI*	Reverse†	Similarity‡
Pyridine, 3-ethyl-	536-78-7	646085	-	Het	pyridine	814	0.72	92	961	962	961	953
3-Ethylcyclopentanone	10264-55-8	126601	-	Oxy	ketone	816	0.995	83	962	965	941	884
Benzene, 1-ethyl-2-methyl-	611-14-3	487535	-	HC	monocyclic aromatic HC	820	0.44	105	964	965	921	920
Pentanoic acid, 3-methyl-	105-43-1	617905	-	Oxy	carboxylic acid	824	0.515	60	965	968	929	826
2-Butanone, 1-(acetyloxy)-	1575-57-1	519029	-	Oxy	ketone	824	1.17	57	966	969	874	816
Benzaldehyde	100-52-7	293884	M	Oxy	aldehyde	826	0.84	106	966	968	937	890
3-Methyl-2-cyclopent-1-one	2758-18-1	1796553	-	Oxy	ketone	826	1.38	96	967	969	945	943
2-Furancarboxaldehyde, 5-methyl-	620-02-0	582178	M	Het	furan	828	1.07	110	967	969	951	887
Pyridine, 3-ethenyl-	1121-55-7	2424660	-	Het	pyridine	836	0.69	105	971	972	936	933
Benzene, 1,2,4-trimethyl-	95-63-6	233187	M	HC	monocyclic aromatic HC	838	0.44	105	972	976	942	931
1,3-Dimethyltrisulfane	3658-80-8	50862	-	Misc	trisulfide	840	0.49	126	973	976	935	828
2-Methyl-6-methylene-1,7-octadiene	1686-30-2	61641	-	HC	alkene	852	0.375	79	978	981	885	784
3-Methyl-2(5H)-furnanone	22122-36-7	216625	-	Oxy	ketone	856	1.73	98	981	982	914	763
Benzene, 1-ethyl-4-methyl-	622-96-8	70791	-	HC	monocyclic aromatic HC	858	0.46	120	981	977	969	919
Benzene, (1-methylethenyl)-	98-83-9	44377	G2B	HC	monocyclic aromatic HC	864	0.47	118	984	986	918	846
Pyridine, 3,4-dimethyl-	583-58-4	76132	-	Het	pyridine	864	0.79	107	984	986	914	794
5-Hepten-2-one, 6-methyl-	110-93-0	565929	-	Oxy	ketone	868	0.77	43	986	986	929	897
Phenol	108-95-2	14254161	G3	Oxy	alcohol	870	0.49	94	986	987	965	963
Benzonitrile	100-47-0	1356953	M	Nit	nitrile	876	1.02	103	989	987	952	941
beta-Myrcene	123-35-3	351424	G2B	HC	monoterpene	878	0.36	69	990	990	909	891

Continued on next page

Table S3. Tentatively identified compounds from the particulate phase fraction of mainstream tobacco smoke (Continued)

Tentative Identification	CAS	Peak Area	Health Effects	Class	Subclass	$^{1}t_R$ (s)	$^{2}t_R$ (s)	Quant Mass	Exp. RI*	Lib. RI*	Reverse†	Similarity‡
Pyridine, 2,4,6-trimethyl-	111-13-7	98961	-	Het	pyridine	880	0.495	121	991	993	865	808
2-Octanone	108-75-8	176216	-	Oxy	ketone	880	0.8	58	991	991	861	776
1,2,3-Trimethylcyclohexane	1678-81-5	199774	-	HC	alicyclic HC	882	0.315	69	992	NA	840	817
Cyclopropene, 3-(1-methylethenyl)-1-(1-methylethyl)-	NA	76431	-	HC	alicyclic HC	884	0.435	91	993	NA	934	766
2-Cyclopenten-1-one, 3,4-dimethyl-	30434-64-1	939188	-	Oxy	ketone	886	1.21	95	994	986	885	890
Pyridine, 2-propyl-	622-39-9	217828	-	Het	pyridine	890	0.51	93	996	998	912	784
Benzene, 1-ethenyl-3-methyl-	100-80-1	414045	G3	HC	monocyclic aromatic HC	894	0.49	117	997	991	769	854
2-Cyclohexen-1-one, 6-(1-hydroxy-1-methylethyl)-3-methyl-	37902-41-3	275954	-	Oxy	ketone	894	0.87	82	998	NA	749	770
5-Hydroxy-2-heptanone	NA	1280604	-	Oxy	ketone	894	0.9	67	998	NA	924	890
1,5-Heptadiene, 2,3,6-trimethyl-	33501-88-1	322460	-	HC	alkene	896	0.315	69	998	NA	904	879
Pyrazine, 2-ethyl-6-methyl-	13925-03-6	408895	-	Het	pyrazine	896	0.53	121	998	998	914	906
Benzofuran	271-89-6	138824	-	Het	furan	898	0.54	90	999	1000	879	850
Decane	5584-69-0	99700	-	HC	alkane	900	0.31	57	1000	1000	924	870
2-Furanone, 2,5-dihydro-3,5-dimethyl	124-18-5	431799	X	Het	furan	900	1.73	69	1001	993	874	847
Benzene, 1-ethenyl-4-methyl-	622-97-9	90145	-	HC	monocyclic aromatic HC	906	0.48	118	1003	995	754	840
Pyrazine, trimethyl-	14667-55-1	1430567	-	Het	pyrazine	906	0.51	42	1003	1002	975	917
Pyrazine, 2-ethyl-3-methyl-	15707-23-0	109963	-	Het	pyrazine	906	0.52	121	1003	1003	836	827
Butanamide, 3-methyl-	541-46-8	624114	-	Nit	amide	912	1.15	59	1006	NA	909	799
Pyridine, 2,3,6-trimethyl-	1462-84-6	97524	-	Het	pyridine	914	0.48	120	1007	1009	873	847

Continued on next page

Table S3. Tentatively identified compounds from the particulate phase fraction of mainstream tobacco smoke (Continued)

Tentative Identification	CAS	Peak Area	Health Effects	Class	Subclass	$^{1}t_R$ (s)	$^{2}t_R$ (s)	Quant Mass	Exp. RI*	Lib. RI*	Reverse†	Similarity‡
2-Hydroxy-gamma-butyrolactone	19444-84-9	1653501	-	Oxy	lactone	916	1.325	57	1008	NA	904	873
1-Pyrrolidinylacetonitrile	29134-29-0	222313	X	Het	pyridine	920	0.92	82	1010	NA	945	935
1-Propanone, 1-(2-furanyl)-	3194-15-8	186103	-	Het	furan	922	0.84	95	1011	1011	947	761
3-Pyridine carbonitrile	100-54-9	445395	X	Het	pyridine	926	1.12	104	1013	1012	958	864
1-Decene, 4-methyl-	13151-29-6	50017	-	HC	alkene	930	0.325	57	1015	NA	854	753
5,6,7,8-Tetrahydroindolizine	13618-88-7	259913	-	Nit	aza-arene	930	0.535	120	1015	NA	809	843
Glycerin	56-81-5	72303956	M	Oxy	alcohol	932	0.685	61	1016	NA	922	920
N,3-Dimethylaniline	696-44-6	463788	-	Nit	amine	938	0.525	120	1019	NA	777	822
gamma-Terpinene	99-85-4	25541	M	HC	monoterpene	940	0.36	93	1020	1024	753	860
p-Methylanisole	22748-16-9	159767	-	Oxy	ether	948	0.52	122	1024	1024	922	894
4,4-Dimethyl-2-cyclopenten-1-one	104-93-8	542221	X	Oxy	ketone	948	1.34	95	1024	NA	884	874
Benzene, 1,2,3-trimethyl-	526-73-8	858011	M	HC	monocyclic aromatic HC	950	0.47	105	1025	1028	925	926
4-Isopropylpyridine	5749-72-4	101837	-	Het	pyridine	952	0.7	106	1026	1021	864	760
Cyclohexane, (1-methylethylidene)-	696-30-0	225112	-	HC	alicyclic HC	952	0.78	81	1026	NA	822	783
Cyclohexene, 3-methyl-6-(1-methylethyl)-	5256-65-5	526209	-	HC	alicyclic HC	954	0.35	67	1026	NA	917	916
o-Cymene	56613-80-0	675476	-	HC	monocyclic aromatic HC	956	0.39	119	1027	1027	946	937
d(-)-á-Phenylglycinol	765-70-8	237249	-	Oxy	alcohol	956	0.56	106	1028	NA	808	853
1,2-Cyclopentanedione, 3-methyl-	527-84-4	3253642	-	Oxy	ketone	956	0.835	112	1028	NA	963	959
Spiro[2.4]heptan-5-one	19740-31-9	134988	-	Oxy	ketone	960	0.94	110	1030	NA	855	841
1-Hexanol 2-ethyl-	104-76-7	1229495	M	Oxy	alcohol	962	0.465	57	1030	1030	953	905

Continued on next page

Table S3. Tentatively identified compounds from the particulate phase fraction of mainstream tobacco smoke (Continued)

Tentative Identification	CAS	Peak Area	Health Effects	Class	Subclass	^1t_R (s)	^2t_R (s)	Quant Mass	Exp. RI*	Lib. RI*	Reverse†	Similarity‡
Benzene, cyclopropyl-	873-49-4	703538	M	HC	monocyclic aromatic HC	962	0.48	117	1030	1032	919	945
D-Limonene	5989-27-5	4202273	G3	HC	monoterpene	966	0.35	68	1032	NA	949	948
Pyridine, 3-ethyl-4-methyl-	933-11-9	69006	M	Het	pyridine	968	0.59	106	1033	NA	854	768
1-Methylcyclooctene	529-21-5	355142	-	HC	alkene	968	0.795	96	1033	1023	835	820
1,3,5-Cycloheptatriene, 7,7-dimethyl-	1122-62-9	74150	M	HC	alicyclic HC	974	0.49	105	1036	NA	846	777
1-(2-Pyridinyl)ethanone	05/07/7533	232753	-	Het	pyridine	974	0.74	79	1036	1036	920	875
trans-beta-Ocimene	1193-79-9	256261	-	HC	monoterpene	976	0.34	93	1037	1037	874	885
2-Acetyl-5-methylfuran	1121-05-7	128502	-	Het	furan	976	1	124	1037	1037	896	867
2-Cyclopenten-1-one, 2,3-dimethyl-	13877-91-3	5811666	-	Oxy	ketone	976	1.08	67	1037	1036	892	893
Benzene, 1-ethenyl-2-methyl-	611-15-4	399199	M	HC	monocyclic aromatic HC	980	0.45	117	1039	NA	845	821
Cyclopentanone, 2-ethyl-	4971-18-0	123467	-	Oxy	ketone	980	0.81	84	1039	NA	859	776
3,4,5,6-Tetrahydrophthalic anhydride	2426-02-0	280177	-	Oxy	anhydride	982	1.03	79	1040	NA	857	792
(3E)-3,7-Dimethyl-1,3,7-octatriene	100-51-6	124778	M	HC	alkene	986	0.345	93	1042	1042	882	842
Benzyl alcohol	502-99-8	210470	-	Oxy	alcohol	986	0.58	79	1042	1041	930	896
Pyridine, 5-ethenyl-2-methyl-	872-50-4	130749	M	Het	pyridine	988	0.675	118	1043	1040	756	791
2-Pyrrolidinone, 1-methyl-	140-76-1	103660	X	Het	pyrrole	988	1.55	99	1044	1045	946	769
Benzene, 3-butenyl-	768-56-9	199958	-	HC	monocyclic aromatic HC	994	0.42	91	1046	1038	888	841
Pyridine, 2-(2-methylpropyl)-	6304-24-1	181614	-	Het	pyridine	996	0.49	93	1047	NA	878	848
(3E)-3,7-Dimethyl-1,3,6-octatriene	3779-61-1	341126	-	HC	alkene	998	0.345	93	1048	NA	926	910

Continued on next page

Table S3. Tentatively identified compounds from the particulate phase fraction of mainstream tobacco smoke (Continued)

Tentative Identification	CAS	Peak Area	Health Effects	Class	Subclass	$^{1}t_R$ (s)	$^{2}t_R$ (s)	Quant Mass	Exp. RI*	Lib. RI*	Reverse†	Similarity‡
3-Hydroxy-4,4-dimethyldihydro-2(3H)-furanone	52126-90-6	425299	-	Oxy	ketone	998	1.265	71	1048	NA	942	916
Indene	95-13-6	1195501	-	HC	polycyclic aromatic HC	1000	0.51	116	1049	1049	928	928
1,2-Diethylbenzene	135-01-3	42400	M	HC	monocyclic aromatic HC	1002	0.4	119	1050	1048	794	754
Benzene, 1-methyl-3-propyl-	1074-43-7	312320	-	HC	monocyclic aromatic HC	1010	0.4	105	1054	1054	846	883
Pyridine 3-propyl-	4673-31-8	444053	-	Het	pyridine	1014	0.675	92	1056	NA	876	865
2-Tolyloxirane	2783-26-8	287737	-	Oxy	epoxide	1020	0.4	105	1058	NA	866	861
Pyridine, 3-ethyl-5-methyl-	1193-18-6	84660	-	Het	pyridine	1020	0.6	121	1059	NA	797	845
2-Cyclohexen-1-one, 3-methyl-	3999-78-8	315740	-	Oxy	ketone	1020	1.205	82	1059	NA	954	897
Acetic acid, phenyl ester	122-79-2	370084	-	Oxy	ester	1022	0.83	94	1060	1065	955	924
Phenol, 2-methyl-	95-48-7	3522348	G3	Oxy	alcohol	1024	0.49	108	1060	1060	951	950
2-Cyclopenten-1-one, 2,3,4-trimethyl-	28790-86-5	982342	-	Oxy	ketone	1026	0.94	109	1062	1063	896	879
Pyridine, 2-ethyl-4,6-dimethyl-	1124-35-2	103131	-	Het	pyridine	1038	0.425	134	1067	1080	797	767
Benzene, 1-methyl-2-propyl-	3658-77-3	254982	M	HC	monocyclic aromatic HC	1040	0.44	105	1068	1067	901	887
2,5-Dimethyl-4-hydroxy-3(2H)-furanone	1074-17-5	193748	-	Het	furan	1040	0.675	57	1068	1068	927	811
Pyridine, 2,3,5-trimethyl-	695-98-7	70191	-	Het	pyridine	1042	0.61	121	1069	1076	894	806
2-Acetylpyrrole	98-86-2	533682	M	Het	pyrrole	1044	0.78	94	1070	1072	907	926
Acetophenone	1072-83-9	1246782	M	Oxy	ketone	1044	0.84	105	1070	1070	974	972

Continued on next page

Table S3. Tentatively identified compounds from the particulate phase fraction of mainstream tobacco smoke (Continued)

Tentative Identification	CAS	Peak Area	Health Effects	Class	Subclass	^1t_R (s)	^2t_R (s)	Quant Mass	Exp. RI*	Lib. RI*	Reverse†	Similarity‡
3-Methylbenzaldehyde	620-23-5	318144	-	Oxy	aldehyde	1046	0.91	90	1071	NA	952	925
2-Ethyl-3,5-dimethylpyridine	1123-96-2	40278	-	Het	pyridine	1048	0.415	134	1072	NA	863	771
Pyridine, 5-ethyl-2-methyl-	104-90-5	66317	M	Het	pyridine	1054	0.705	106	1075	NA	887	810
Benzaldehyde, 2-methyl-	529-20-4	160338	-	Oxy	aldehyde	1054	0.79	91	1075	1068	814	907
Pyrazine, 3-ethyl-2,5-dimethyl-	21834-98-0	99952	-	Het	pyrazine	1058	0.46	136	1077	1077	885	847
2-Hydroxy-3,5-dimethylcyclopent-2-en-1-one	5682-69-9	122759	-	Oxy	ketone	1058	0.825	111	1077	NA	780	986
2-Cyclopenten-1-one, 3-ethyl-	13360-65-1	1045353	-	Oxy	ketone	1058	1.23	81	1077	1071	910	873
Benzene, 2-ethyl-1,4-dimethyl-	1758-88-9	174002	-	HC	monocyclic aromatic HC	1060	0.42	119	1078	1077	926	906
Benzene, 1-ethyl-2,4-dimethyl-	616-45-5	391304	M	HC	monocyclic aromatic HC	1066	0.405	119	1081	1080	906	910
Octanenitrile	124-12-9	64009	-	Nit	nitrile	1066	0.97	82	1081	1082	887	771
2-Pyrrolidinone	874-41-9	962149	X	Het	pyrrole	1066	1.44	85	1081	1078	929	848
p-Cresol	106-44-5	9677027	M	Oxy	alcohol	1068	0.48	107	1082	1082	950	950
Pyrazine, 2-ethyl-3,5-dimethyl-	13925-07-0	118710	-	Het	pyrazine	1070	0.455	135	1083	1083	830	783
1,10-Undecadiene	13688-67-0	40492	-	HC	alkene	1072	0.33	67	1084	1085	870	779
5-Ethyl-2,3-dimethylpyrazine	22433-39-2	97513	-	Het	pyrazine	1076	0.465	135	1086	1087	962	833
Benzene,1-methyl-1,2-propadienyl-	15707-34-3	134797	-	HC	monocyclic aromatic HC	1076	0.48	115	1086	NA	832	847
Benzene, 1-methyl-3-(1-methylethyl)-	38877-21-3	410434	-	HC	monocyclic aromatic HC	1078	0.4	119	1087	NA	923	912
2,3-Dihydro-5-hydroxy-6-methyl-4H-pyran-4-one	535-77-3	1207247	X	Het	pyran	1078	0.775	43	1087	1088	974	918

Continued on next page

Table S3. Tentatively identified compounds from the particulate phase fraction of mainstream tobacco smoke (Continued)

Tentative Identification	CAS	Peak Area	Health Effects	Class	Subclass	$^{1}t_R$ (s)	$^{2}t_R$ (s)	Quant Mass	Exp. RI*	Lib. RI*	Reverse†	Similarity‡
1,5-Heptadiene, 2,5-dimethyl-3-methylene-	90-05-1	178834	G2B	HC	alkene	1082	0.325	93	1089	NA	892	877
1H-Indene, 2,3-dihydromethyl-	7251-61-8	63330	M	HC	polycyclic aromatic HC	1082	0.43	132	1089	1087	919	873
Guaiacol	620-22-4	3446021	-	Oxy	alcohol	1082	0.67	109	1089	1089	937	933
3-Methylbenzonitrile	27133-93-3	93252	-	Nit	nitrile	1082	0.81	91	1089	1086	910	796
2-Nonanone	81396-36-3	252097	-	Oxy	ketone	1086	0.72	58	1091	1091	918	859
1-Undecene	821-55-6	294508	-	HC	alkene	1088	0.32	56	1091	1091	928	928
2,5-Pyrrolidinedione, 1-methyl-	1121-07-9	251451	X	Het	pyrrole	1088	1.505	113	1092	NA	951	891
4,5-Dimethyl-4-hexen-3-one	17325-90-5	210882	-	Oxy	ketone	1092	1.49	97	1094	NA	921	794
Benzonitrile, 2-methyl-	529-19-1	354574	X	Nit	nitrile	1094	0.98	116	1095	NA	941	927
3-Undecene, (Z)-	821-95-4	37958	-	HC	alkene	1098	0.305	69	1096	1092	754	844
Undecane	23074-10-4	491737	-	HC	alkane	1104	0.29	57	1099	1100	952	933
5-Ethyl-2-furaldehyde	1120-21-4	240551	X	Het	furan	1104	1.09	109	1100	NA	850	812
Benzofuran, 7-methyl-	17059-52-8	129716	-	Het	furan	1106	0.505	131	1100	NA	850	863
3-Ethenyl-3-methylcyclopentanone	49664-66-6	981873	-	Oxy	ketone	1108	1.11	68	1102	NA	770	773
Benzene, 1-methyl-4-(2-methylpropyl)-	06/04/5161	248723	-	HC	monocyclic aromatic HC	1110	0.395	105	1102	1105	882	873
2-Methyl-6-propylpyridine	5397-28-4	183484	-	Het	pyridine	1110	0.495	107	1102	NA	824	779
1,3-Dimethyl-2-vinylbenzene	24081-57-0	48068	-	HC	monocyclic aromatic HC	1112	0.44	132	1103	NA	865	787
1-(1,5-Dimethylbicyclo[2.1.0]pent-5-yl)ethanone	218-029-4	114146	-	Oxy	ketone	1112	0.835	95	1104	NA	793	753
2-Undecene, (E)-	693-61-8	44179	X	HC	alkene	1114	0.3	70	1104	1104	863	899

Continued on next page

Table S3. Tentatively identified compounds from the particulate phase fraction of mainstream tobacco smoke (Continued)

Tentative Identification	CAS	Peak Area	Health Effects	Class	Subclass	$^{1}t_R$ (s)	$^{2}t_R$ (s)	Quant Mass	Exp. RI*	Lib. RI*	Reverse†	Similarity‡
Benzene, 2-ethyl-1,3-dimethyl-	04/04/2870	158420	-	HC	monocyclic aromatic HC	1118	0.43	119	1107	1105	775	860
2,6-Diethylpyridine	6940-57-4	28221	-	Het	pyridine	1118	0.505	135	1107	NA	872	759
2-Acetly-6-methylpyridine	93518-56-0	63460	-	Het	pyridine	1118	0.675	93	1107	1107	853	790
2,3-Dimethylanisole	1575-46-8	46805	-	Oxy	ether	1122	0.45	121	1109	1111	866	801
Benzonitrile, 4-methyl-	2944-49-2	283505	-	Nit	nitrile	1122	1	117	1109	NA	929	896
2,3-Dimethyl-4-hydroxy-2-butenoic lactone	104-85-8	307761	X	Oxy	ester	1122	1.88	55	1110	NA	920	843
6,7-Dihydro-5H-cyclopentapyrazine	626-97-1	87336	-	Het	pyrazine	1124	0.61	119	1110	1104	948	818
Pentanamide	NA	534540	-	Nit	amide	1124	1.045	59	1110	NA	765	854
Phenol, 2,6-dimethyl-	74744-54-0	642988	-	Oxy	alcohol	1126	0.5	107	1111	1108	945	933
2-Acetyl-4-methylthiazole	576-26-1	30286	X	Nit	thiazole	1126	0.64	113	1111	1118	950	807
Benzofuran, 2-methyl-	4265-25-2	609985	-	Het	furan	1128	0.53	131	1112	1117	930	924
Cyclohexane, 1-methyl-4-(1-methylethylidene)-	350-03-8	60114	M	HC	monoterpene	1130	0.725	95	1113	NA	838	789
1-(3-Pyridinyl)ethanone	1124-27-2	577365	-	Het	pyridine	1130	1.02	78	1113	1117	928	887
(E)-4,8-Dimethylnona-1,3,7-triene	19945-61-0	337089	-	HC	alicyclic HC	1132	0.34	69	1114	1114	883	855
2,3-Cyclopentenopyridine	533-37-9	365157	-	Het	pyridine	1132	0.66	118	1114	NA	873	821
Benzene, 1-ethyl-4-methoxy-	118-71-8	236537	M	Oxy	ether	1136	0.47	121	1116	1122	929	901
1H-Indole, 2,3-dihydro-	496-15-1	123433	-	Nit	aza-arene	1136	0.58	118	1116	NA	871	831
Maltol	1515-95-3	822603	-	Oxy	alcohol	1136	0.91	126	1116	1122	926	916
2-Acetyl-3-methylpyrazine	54458-61-6	85387	-	Het	pyrazine	1138	0.78	94	1118	1128	810	752
2-Cyclopenten-1-one, 2,3,4,5-tetramethyl-	23787-80-6	206877	-	Oxy	ketone	1138	0.835	123	1118	NA	858	846
3-Pyridinyl acetate	17747-43-2	215416	-	Het	pyridine	1138	1.015	95	1118	1127	899	784

Continued on next page

Table S3. Tentatively identified compounds from the particulate phase fraction of mainstream tobacco smoke (Continued)

Tentative Identification	CAS	Peak Area	Health Effects	Class	Subclass	^1t_R (s)	^2t_R (s)	Quant Mass	Exp. RI*	Lib. RI*	Reverse†	Similarity‡
Bicyclo[5.1.0]octane, 8-(1-methylethylidene)-	54166-47-1	106957	-	HC	polycyclic aromatic HC	1140	0.325	93	1118	NA	829	791
Benzeneethanol	60-12-8	323888	X	Oxy	alcohol	1140	0.58	92	1118	1117	931	899
Benzene, 1,2,4,5-tetramethyl-	622-80-0	143148	-	HC	monocyclic aromatic HC	1142	0.41	119	1119	1118	857	840
N-Phenyl-N-propylamine	21835-01-8	110715	-	Nit	amine	1142	0.53	106	1119	NA	968	764
Benzoxazole, 2-methyl-	95-21-6	199375	-	Nit	oxazole	1142	0.56	133	1119	NA	910	865
2-Cyclopenten-1-one, 3-ethyl-2-hydroxy-	95-93-2	627333	X	Oxy	ketone	1142	0.77	83	1120	1121	947	926
Pentanamide, 4-methyl-	1119-29-5	646105	-	Nit	amide	1146	1.08	59	1122	NA	836	886
Benzene, 1,2,3,5-tetramethyl-	527-53-7	368949	X	HC	monocyclic aromatic HC	1148	0.42	119	1123	1123	888	899
Benzene, (2-methyl-1-propenyl)-	768-49-0	175001	-	HC	monocyclic aromatic HC	1150	0.45	117	1124	NA	897	869
1-(6-methly-2-pyrazinyl)-1-Ethanone	22047-26-3	58669	-	Het	pyrazine	1152	0.72	136	1125	NA	869	778
Isophorone	78-59-1	597876	X	Oxy	ketone	1154	1.18	82	1126	1126	965	939
2H-cyclopenta[b]furan-2-one, 3,3a,6,6a-tetrahydro-, (3aR-cis)-	43119-28-4	185274	-	Het	furan	1156	1.225	96	1127	NA	829	769
Benzene, 1-butenyl-, (E)-	1005-64-7	456780	-	HC	monocyclic aromatic HC	1158	0.46	117	1128	NA	946	936
3-Pyridinol	109-00-2	4853143	-	Het	pyridine	1158	0.555	95	1128	NA	934	927
Alloocimene	3016-19-1	292071	-	HC	monoterpene	1160	0.37	121	1129	1129	915	908
Benzyl methyl ketone	103-79-7	1456828	-	Oxy	ketone	1162	0.87	43	1130	1124	918	896
2-Ethylidenecyclohexanone	1122-24-3	501173	-	Oxy	ketone	1162	1.01	124	1130	NA	824	879

Continued on next page

Table S3. Tentatively identified compounds from the particulate phase fraction of mainstream tobacco smoke (Continued)

Tentative Identification	CAS	Peak Area	Health Effects	Class	Subclass	$^{1}t_R$ (s)	$^{2}t_R$ (s)	Quant Mass	Exp. RI*	Lib. RI*	Reverse†	Similarity‡
Cosmene	460-01-5	64363	-	HC	monoterpene	1166	0.43	119	1132	1132	905	852
Benzene, 1-methyl-4-(2-propenyl)-	3333-13-9	331903	-	HC	monocyclic aromatic HC	1170	0.455	117	1134	NA	940	920
Benzene, 1,3-dimethyl-5-(1-methylethyl)-	4706-90-5	104349	-	HC	monocyclic aromatic HC	1174	0.38	133	1136	NA	847	798
Benzeneacetaldehyde, á-ethyl-	2439-43-2	80968	-	Oxy	aldehyde	1176	0.385	119	1137	NA	744	767
3-Pyridinol, 2-methyl-	1121-25-1	435522	-	Het	pyridine	1176	0.52	109	1138	NA	938	864
Ethanone, 1-(2-methylphenyl)-	577-16-2	119774	-	Oxy	ketone	1178	0.71	119	1139	1139	954	889
1-Propanone, 1-(2-pyridinyl)-	3238-55-9	87768	-	Het	pyridine	1180	0.62	78	1140	1137	915	819
1-Methylenespiro[2.4]heptan-4-one	166193-08-4	167129	-	Oxy	ketone	1182	0.88	79	1141	NA	847	798
2-Hepten-4-one, 2-methyl-	22319-24-0	147659	-	Oxy	ketone	1182	1.825	83	1141	NA	799	757
Benzene, 1,3-diethyl-5-methyl-	140-29-4	45264	M	HC	monocyclic aromatic HC	1184	0.365	133	1142	1143	837	798
(3-methyl-3-butenyl)Benzene	1619-28-9	284181	-	HC	monocyclic aromatic HC	1184	0.405	91	1142	NA	888	804
Benzyl nitrile	2050-24-0	897570	-	Nit	nitrile	1184	0.98	90	1142	1142	941	939
2-Cyclopenten-1-one, 3-(1-methylethyl)-	6683-51-8	86044	-	Oxy	ketone	1184	1.205	82	1142	NA	916	837
4-Methylindane	874-23-7	481744	-	HC	polycyclic aromatic HC	1188	0.4	117	1144	1140	899	909
Cyclohexanone, 2-acetyl-	821-97-6	98636	-	Oxy	ketone	1188	0.695	140	1144	NA	767	753
2-Ethylhexyl acetate	103-09-3	528671	X	Oxy	ester	1192	0.555	70	1146	1144	923	908
4-Oxoisophorone	1125-21-9	427368	-	Oxy	ketone	1194	1.095	68	1147	1147	905	888

Continued on next page

Table S3. Tentatively identified compounds from the particulate phase fraction of mainstream tobacco smoke (Continued)

Tentative Identification	CAS	Peak Area	Health Effects	Class	Subclass	^1t_R (s)	^2t_R (s)	Quant Mass	Exp. RI*	Lib. RI*	Reverse†	Similarity‡
1,3-Hexadiene, 3-ethyl-2,5-dimethyl-	62338-07-2	177224	-	HC	alkene	1196	0.89	123	1148	NA	798	810
2-Isobutyl-4-methylpyridine	85665-88-9	552818	G3	Het	pyridine	1200	0.47	107	1150	1154	882	868
4H-Pyran-4-one, 2,3-dihydro-3,5-dihydroxy-6-methyl-	28564-83-2	1746635	M	Het	pyran	1202	0.645	101	1151	1151	907	907
3-Cyclopenten-1-one, 2,2,5,5-tetramethyl-	80933-76-2	451997	-	Oxy	ketone	1202	0.855	110	1152	NA	839	827
3-[(1E)-3-methyl-1-butenyl-1-Cyclohexene	56030-49-0	55132	-	HC	alicyclic HC	1204	0.32	81	1152	NA	830	780
1H-Indene, 1-methyl-	767-59-9	456875	-	HC	polycyclic aromatic HC	1206	0.47	115	1153	1149	926	928
2-Cyclohexen-1-one, 3,4,4-trimethyl-	17299-41-1	50433	-	Oxy	ketone	1206	0.845	96	1154	NA	860	771
Phenol, 2,4-dimethyl-	105-67-9	322554	-	Oxy	alcohol	1210	0.46	93	1156	1156	927	916
Benzene, (1-methyl-2-cyclopropen-1-yl)-	65051-83-4	467679	-	HC	monocyclic aromatic HC	1210	0.47	130	1156	NA	928	924
Benzene, 1-ethenyl-4-methoxy-	637-69-4	208631	-	Oxy	ether	1214	0.53	134	1158	1158	880	861
Bicyclo[10.1.0]tridec-1-ene	54766-91-5	164564	-	HC	polycyclic aromatic HC	1216	0.315	67	1159	NA	786	761
Pyridine, 3-butyl-	539-32-2	376844	-	Het	pyridine	1216	0.63	92	1159	NA	928	892
Benzene, pentyl-	538-68-1	349322	-	HC	monocyclic aromatic HC	1218	0.4	92	1160	1160	940	935
Benzene, 1-butynyl-	622-76-4	857567	-	HC	monocyclic aromatic HC	1218	0.5	129	1160	NA	928	933
2,5-Pyrrolidinedione, 3-methyl-	5615-90-7	1752839	-	Het	pyrrole	1222	1.24	42	1162	NA	975	904

Continued on next page

Table S3. Tentatively identified compounds from the particulate phase fraction of mainstream tobacco smoke (Continued)

Tentative Identification	CAS	Peak Area	Health Effects	Class	Subclass	^1t_R (s)	^2t_R (s)	Quant Mass	Exp. RI*	Lib. RI*	Reverse†	Similarity‡
Pyrazine, 2-methyl-6-(1-propenyl)-, (E)-	18217-81-7	76993	-	Het	pyrazine	1224	0.525	133	1163	NA	882	783
Benzene, (1-methylene-2-propenyl)-	13544-59-7	109190	-	HC	monocyclic aromatic HC	1230	0.45	129	1166	NA	894	807
cis-p-mentha-1(7),8-dien-2-ol	2288-18-8	183072	-	Oxy	alcohol	1230	0.485	67	1166	1165	874	868
Naphthalene, 1,2,3,4-tetrahydro-	122-46-3	121012	-	HC	polycyclic aromatic HC	1232	0.42	104	1167	1166	878	843
2-Butanone, 4-(5-methyl-2-furanyl)-	13679-56-6	252019	-	Het	furan	1232	0.71	95	1167	1160	834	773
m-Cresyl acetate	119-64-2	197245	X	Oxy	ester	1232	0.775	108	1167	NA	901	827
Benzene, 1-methyl-4-butyl	1595-05-7	349972	-	HC	monocyclic aromatic HC	1234	0.41	105	1168	1158	826	843
Benzeneacetonitrile, á-methyl-	1823-91-2	120324	-	Nit	nitrile	1234	0.965	116	1169	NA	930	838
trans-p-mentha-1(7),8-dien-2-ol	2102-62-7	266318	-	Oxy	alcohol	1236	0.485	67	1169	NA	856	842
1-Propanone, 1-phenyl-	935-28-4	653705	-	Oxy	ketone	1236	0.71	105	1170	1173	924	919
Egomaketone	59204-74-9	579204	X	Oxy	ketone	1238	0.34	95	1170	NA	798	785
Cycloprop[a]indene, 1,1a,6,6a-tetrahydro-	6909-19-9	241364	-	HC	polycyclic aromatic HC	1240	0.455	130	1172	NA	936	915
Carane, 4,5-epoxy-, trans	20547-99-3	135133	-	Oxy	epoxide	1240	0.995	67	1172	1179	805	769
1,4-Cyclohexanedione, 2,2,6-trimethyl-	15677-15-3	83409	-	Oxy	ketone	1240	1.225	42	1172	1170	901	764
Pyrrolidine, 1-acetyl-	4030-18-6	157478	-	Het	pyrrole	1240	1.38	70	1172	1162	902	808
1H-Pyrrole-2,5-dione, 3,4-dimethyl-	17825-86-4	587037	-	Het	pyrrole	1242	1	125	1173	NA	976	827
6-Ethyl-5,6-dihydro-2H-pyran-2-one	19895-35-3	3978068	-	Oxy	ketone	1244	1.44	68	1174	NA	928	919

Continued on next page

Table S3. Tentatively identified compounds from the particulate phase fraction of mainstream tobacco smoke (Continued)

Tentative Identification	CAS	Peak Area	Health Effects	Class	Subclass	^1t_R (s)	^2t_R (s)	Quant Mass	Exp. RI*	Lib. RI*	Reverse†	Similarity‡
Phenol, 4-ethyl-	123-07-9	7046087	X	Oxy	alcohol	1246	0.47	107	1175	1173	935	934
Benzene, (1-ethylpropyl)-	1196-58-3	165973	-	HC	monocyclic aromatic HC	1250	0.385	119	1177	NA	753	795
Ethanone, 1-(4-methylphenyl)-	122-00-9	779704	X	Oxy	ketone	1250	0.795	119	1177	1177	971	938
Benzonitrile, 3,5-dimethyl-	22445-42-7	293860	-	Nit	nitrile	1254	0.87	116	1179	NA	914	928
Benzene, 1-methyl-4-(1-methyl-2-propenyl)-	1438-94-4	276575	-	HC	monocyclic aromatic HC	1258	0.395	131	1181	1190	871	875
1H-Pyrrole, 1-(2-furanylmethyl)-	97664-18-1	462250	X	Het	pyrrole	1258	0.56	81	1181	1182	922	873
Benzenamine, 2-methoxy-	89-74-7	208219	-	Nit	amine	1260	0.78	108	1182	1181	867	771
1,11-Dodecadiene	5876-87-9	138214	-	HC	alkene	1262	0.3	55	1183	NA	824	876
Phenol, 2,3-dimethyl-	526-75-0	806665	-	Oxy	alcohol	1264	0.48	107	1184	1184	930	925
Ethylidenecyclooctane	19780-51-9	181577	-	HC	alicyclic HC	1270	0.99	95	1188	NA	737	765
Phenol, 2-(1-methylethyl)-	88-60-8	454460	X	Oxy	alcohol	1272	0.47	121	1189	1184	869	904
2-Methoxy-5-methylphenol	1195-09-1	27551	M	Oxy	ether	1274	0.615	138	1190	1191	880	775
2-Cyclohexen-1-one, 4-(1-methylethyl)-	500-02-7	94193	-	Oxy	ketone	1276	0.755	96	1191	1191	754	815
Ethanone, 1-(3-methylphenyl)-	585-74-0	914929	-	Oxy	ketone	1276	0.8	119	1191	1192	943	971
2,5-Dimethylbenzonitrile	13730-09-1	527961	X	Nit	nitrile	1276	0.895	116	1191	NA	816	887
1-Dodecene	5779-94-2	673939	-	HC	alkene	1278	0.3	56	1192	1192	922	919
(5Z)-5-DODECENE	112-41-4	87334	-	HC	alkene	1278	0.305	81	1192	1184	921	922
1H-Indene, 2,3-dihydro-1,1-dimethyl-	7206-19-1	738489	-	HC	polycyclic aromatic HC	1278	0.39	131	1192	NA	895	890
2-Decanone	6909-20-2	268380	-	Oxy	ketone	1278	0.66	58	1192	1192	888	846
Benzaldehyde, 2,5-dimethyl-	4912-92-9	39704	-	Oxy	aldehyde	1278	0.72	133	1192	NA	895	785

Continued on next page

Table S3. Tentatively identified compounds from the particulate phase fraction of mainstream tobacco smoke (Continued)

Tentative Identification	CAS	Peak Area	Health Effects	Class	Subclass	^1t_R (s)	^2t_R (s)	Quant Mass	Exp. RI*	Lib. RI*	Reverse†	Similarity‡
Naphthalene	91-20-3	4101274	G2B	HC	polycyclic aromatic HC	1280	0.55	128	1193	1192	952	956
Phenol, 2-methoxy-4-methyl-	93-51-6	861658	M	Oxy	alcohol	1280	0.64	123	1193	1193	940	938
2-Cyclohexen-1-one, 4-ethyl-4-methyl-	17429-32-2	292580	-	Oxy	ketone	1282	0.99	95	1194	NA	784	753
4-Dodecene, (E)-	2030-84-4	61083	-	HC	alkene	1284	0.29	56	1195	NA	885	846
2-Piperidinone	675-20-7	219287	X	Nit	lactam	1284	1.385	99	1195	NA	938	842
Dodecane	112-40-3	1305887	-	HC	alkane	1292	0.29	57	1199	1200	936	933
(-)-alpha-Terpineol	32780-06-6	70758	-	Oxy	alcohol	1292	0.465	59	1199	1192	895	832
(S)-(+)-2',3'-Dideoxyribonolactone	10482-56-1	2880815	X	Oxy	lactone	1292	1.505	85	1200	NA	913	901
Benzene, 1,4-diethyl-2-methyl-	13632-94-5	154210	-	HC	monocyclic aromatic HC	1294	0.38	133	1200	NA	877	806
4H-Pyran-4-one, 3,5-dihydroxy-2-methyl-	1073-96-7	146818	-	Het	pyran	1294	0.64	142	1200	1205	857	752
1,5-Cyclohexadiene-1-carboxyaldehyde, 4,4-dimethyl-	97997-08-5	61260	-	Oxy	aldehyde	1300	0.69	91	1204	NA	922	765
Acetone peroxide	1073-91-2	735872	-	Oxy	peroxide	1300	1.81	43	1205	NA	915	778
3-Dodecene, (Z)-	7206-28-2	270361	-	HC	alkene	1302	0.29	56	1205	NA	877	871
p-Ethylbenzonitrile	25309-65-3	77236	-	Nit	nitrile	1302	0.93	116	1205	NA	915	805
2,3-Dimethylbenzonitrile	5724-56-1	189138	X	Nit	nitrile	1308	0.89	116	1209	NA	841	879
Phenol, 2,4,6-trimethyl-	527-60-6	399622	M	Oxy	alcohol	1310	0.47	121	1210	1211	964	915
Benzene, 1-ethyl-4-(1-methylethyl)-	4218-48-8	117499	-	HC	monocyclic aromatic HC	1312	0.38	133	1211	NA	876	824
Benzene, 1,2-diethyl-4,5-dimethyl-	52095-40-6	52769	-	HC	monocyclic aromatic HC	1314	0.365	119	1212	NA	808	777

Continued on next page

Table S3. Tentatively identified compounds from the particulate phase fraction of mainstream tobacco smoke (Continued)

Tentative Identification	CAS	Peak Area	Health Effects	Class	Subclass	$^{1}t_{R}$ (s)	$^{2}t_{R}$ (s)	Quant Mass	Exp. RI*	Lib. RI*	Reverse†	Similarity‡
1-(2-Vinylphenyl)ethanone	19961-07-0	384294	-	Oxy	ketone	1314	0.485	131	1212	NA	809	816
Undecane, 2,6-dimethyl-	17301-23-4	1547981	-	HC	alkane	1316	0.3	57	1213	1213	947	929
Benzenamine, 2-(1-methylethenyl)-	52562-19-3	73592	-	Nit	amine	1316	0.62	117	1213	NA	841	779
5,6,7,8-Tetrahydroquinoxaline	34413-35-9	52179	-	Nit	aza-arene	1318	0.535	134	1214	1212	899	852
Ethyl 4-pyridyl ketone	1701-69-5	74249	X	Het	pyridine	1320	0.875	106	1215	NA	865	760
2-Caren-10-ol	6846-50-0	54218	X	Oxy	alcohol	1324	0.77	107	1218	NA	754	804
Benzofuran, 4,7-dimethyl-	28715-26-6	124672	-	Het	furan	1326	0.5	145	1219	1220	883	872
1-Nonanol, 4,8-dimethyl-	33933-80-1	217734	-	Oxy	alcohol	1328	0.3	56	1220	1229	822	853
Cyclohexanone, 2-(1-methylethylidene)-	13747-73-4	170885	-	Oxy	ketone	1330	0.945	67	1221	NA	806	785
Cinnamaldehyde, á-methyl-	1196-67-4	827995	X	Oxy	aldehyde	1332	0.495	145	1222	NA	787	795
6-Dodecyne	6975-99-1	206421	-	HC	alkyne	1338	0.325	54	1226	NA	850	837
Benzene, 2-ethenyl-1,3,5-trimethyl-	769-25-5	251147	-	HC	monocyclic aromatic HC	1338	0.405	131	1226	1224	914	900
4-Methyl-2H-chromene	21776-94-3	136967	-	Het	pyran	1338	0.51	145	1226	NA	764	838
(Z)-3-Phenylacrylaldehyde	57194-69-1	31949	-	Oxy	aldehyde	1340	0.82	131	1227	1223	754	843
Phenol, 2-propyl-	1007-32-5	385352	-	Oxy	alcohol	1342	0.435	107	1228	1229	755	778
1-Phenyl-2-butanone	20548-00-9	224688	-	Oxy	ketone	1342	0.74	57	1228	NA	888	795
4-Methyleneisophorone	644-35-9	69978	X	Oxy	ketone	1342	0.945	107	1228	1224	860	804
3-Phenylfuran-	13679-41-9	147003	-	Het	furan	1344	0.53	115	1229	1228	917	800
2,3-Dihydro-benzofuran	496-16-2	3841449	-	Het	furan	1348	0.48	120	1231	1226	911	903
Phenol, 3-(1-methylethyl)-	618-45-1	970578	X	Oxy	alcohol	1352	0.435	121	1234	1229	830	899
2,5-Dimethylbenzoxazole	17583-40-3	57595	-	Nit	oxazole	1354	0.535	147	1235	NA	890	844
Benzonitrile, 2-(methylamino)-	5676-58-4	411641	X	Nit	nitrile	1354	0.59	131	1235	NA	888	880
Phenol, 4-ethyl-3-methyl-	1123-94-0	423310	-	Oxy	alcohol	1360	0.43	121	1238	1239	832	889

Continued on next page

Table S3. Tentatively identified compounds from the particulate phase fraction of mainstream tobacco smoke (Continued)

Tentative Identification	CAS	Peak Area	Health Effects	Class	Subclass	$^{1}t_{R}$ (s)	$^{2}t_{R}$ (s)	Quant Mass	Exp. RI*	Lib. RI*	Reverse†	Similarity‡
Isoquinoline, 3,4-dihydro-	3230-65-7	550157	-	Nit	aza-arene	1360	0.645	130	1238	NA	834	821
Phenol, 2,3,6-trimethyl-	20189-42-8	115369	-	Oxy	alcohol	1362	0.475	120	1239	1239	885	833
1H-Pyrrole-2,5-dione, 3-ethyl-4-methyl-	2416-94-6	468503	X	Het	pyrrole	1362	0.94	139	1240	1239	925	907
Benzene, 3-hexenyl-	35008-86-7	231660	-	HC	monocyclic aromatic HC	1364	0.385	91	1241	NA	860	760
Benzene, 1-methoxy-2-(1-methylethenyl)-	10278-02-1	53397	-	Oxy	ether	1368	0.685	133	1243	NA	780	822
Benzenepropanenitrile	645-59-0	2605873	-	Nit	nitrile	1368	1.01	91	1243	1243	944	936
Diacetin	25395-31-7	2874087	X	Oxy	ester	1370	1.065	43	1244	NA	957	952
Benzene, [(2,2-dimethylcyclopropyl)methyl]-	36939-18-1	783620	-	HC	monocyclic aromatic HC	1372	0.375	69	1245	NA	825	820
2-Butanone, 4-phenyl-	2550-26-7	179662	-	Oxy	ketone	1374	0.79	105	1246	1251	892	881
Isoquinoline	119-65-3	771054	M	Nit	aza-arene	1378	0.63	129	1249	1255	950	942
Phenol, 3-ethyl-5-methyl-	698-71-5	311869	-	Oxy	alcohol	1380	0.445	121	1250	NA	870	900
2-Hexanoylfuran	14360-50-0	134937	-	Het	furan	1380	0.79	110	1250	1239	839	762
2-Ethyl-1-H-indene	17059-50-6	292758	-	HC	polycyclic aromatic HC	1382	0.44	129	1251	NA	861	784
Benzene, 1-methyl-3-(1-methyl-2-propenyl)-	52161-57-6	208093	-	HC	monocyclic aromatic HC	1390	0.385	131	1255	NA	847	841
Ethanone, 1-(2,4-dimethylphenyl)-	89-72-5	49494	X	Oxy	ketone	1390	0.67	133	1256	NA	893	810
(3E)-3-Nonen-1-ol	10339-61-4	76837	-	Oxy	alcohol	1392	0.315	68	1257	NA	842	808
1H-Indene, 1,1-dimethyl-	32267-71-3	380513	-	HC	polycyclic aromatic HC	1392	0.445	129	1257	NA	858	808

Continued on next page

Table S3. Tentatively identified compounds from the particulate phase fraction of mainstream tobacco smoke (Continued)

Tentative Identification	CAS	Peak Area	Health Effects	Class	Subclass	$^{1}t_R$ (s)	$^{2}t_R$ (s)	Quant Mass	Exp. RI*	Lib. RI*	Reverse†	Similarity‡
2(3H)-Benzofuranone, 3-methyl-	18636-55-0	36565	-	Oxy	ketone	1392	0.875	120	1257	NA	935	755
(2E,4Z)-2,4-Dodecadiene	939-23-1	195690	M	HC	alkene	1400	0.32	68	1261	NA	814	799
Naphthalene, 1,2,3,4-tetrahydro-1,1,6-trimethyl-	74663-83-5	39403	-	HC	polycyclic aromatic HC	1400	0.35	159	1261	1264	764	786
4-Ethylphenyl acetate	3245-23-6	126587	-	Oxy	ester	1400	0.73	107	1261	NA	869	798
Benzene, (1-methylpentyl)-	03/01/6031	273787	-	HC	monocyclic aromatic HC	1402	0.365	105	1262	NA	854	811
Naphthalene, 1,2-dihydro-2-methyl-	21564-79-4	1513265	-	HC	polycyclic aromatic HC	1402	0.44	129	1262	NA	888	896
Benzene, hexyl-	1077-16-3	324906	-	HC	monocyclic aromatic HC	1404	0.37	92	1263	1263	884	878
Dodecane, 2-methyl-	1560-97-0	88630	-	HC	alkane	1406	0.29	57	1265	1265	896	827
Phenol, 4-propyl-	645-56-7	913975	-	Oxy	alcohol	1410	0.45	107	1267	1262	896	851
Ethanone, 1-(2,5-dimethylphenyl)-	2142-73-6	153409	-	Oxy	ketone	1410	0.735	133	1267	NA	820	863
2-Acetoxy-3-methyl-2-cyclopenten-1-one	1196-22-1	92929	-	Oxy	ketone	1414	1.365	112	1270	NA	943	890
Benzeneacetaldehyde	122-78-1	77589	X	Oxy	aldehyde	1416	0.98	51	1271	NA	906	851
2-Propyl-4-methylphenol	91-22-5	103039	G2B	Oxy	alcohol	1420	0.455	121	1273	NA	764	853
Quinoline	4074-46-8	311612	-	Nit	aza-arene	1420	0.7	129	1273	1255	946	900
1-Propanone, 1-(2-methylphenyl)-	603-76-9	309360	-	Oxy	ketone	1424	0.685	119	1275	NA	928	881
1H-Indole, 1-methyl-	2040-14-4	152948	-	Nit	aza-arene	1424	0.71	131	1275	1277	908	879
1H-Indene, 2,3-dimethyl-	4773-82-4	1149600	-	HC	polycyclic aromatic HC	1426	0.48	129	1276	NA	909	903

Continued on next page

Table S3. Tentatively identified compounds from the particulate phase fraction of mainstream tobacco smoke (Continued)

Tentative Identification	CAS	Peak Area	Health Effects	Class	Subclass	^1t_R (s)	^2t_R (s)	Quant Mass	Exp. RI*	Lib. RI*	Reverse†	Similarity‡
Benzene, 1-cyclopenten-1-yl-	824-22-6	145416	-	HC	polycyclic aromatic HC	1430	0.425	143	1278	NA	857	780
Benzene, 1,4-dimethyl-2-(2-methylpropyl)-	2785-89-9	54389	-	HC	monocyclic aromatic HC	1432	0.365	119	1280	NA	845	753
Phenol, 4-ethyl-2-methoxy-	55669-88-0	768057	-	Oxy	alcohol	1432	0.6	137	1280	1280	939	923
3,4-Dimethyl-3-pyrrolin-2-one	4030-22-2	183043	-	Het	pyrrole	1432	1.285	111	1280	NA	893	758
Ethanone, 1-(2,5-dihydroxyphenyl)-	490-78-8	368949	X	Oxy	ketone	1440	0.695	137	1284	NA	907	853
Benzonitrile, 4-(1-methylethyl)-	13816-33-6	79513	X	Nit	nitrile	1440	0.845	130	1284	NA	845	783
Phenol, 2-ethyl-5-methyl-	875-59-2	247139	-	Oxy	alcohol	1442	0.48	121	1285	NA	823	878
1H-Pyrrole, 1-phenyl-	635-90-5	84898	-	Het	pyrrole	1442	0.53	143	1285	NA	882	831
4-Hydroxy-2-methylacetophenone	1687-61-2	64225	X	Oxy	ketone	1442	0.725	135	1286	NA	782	890
1H-Indene, 2,3-dihydro-4,7-dimethyl-	83-33-0	124652	-	HC	polycyclic aromatic HC	1446	0.395	131	1288	1282	830	815
1H-Inden-1-one, 2,3-dihydro-	6682-71-9	1611858	-	Oxy	ketone	1446	0.84	104	1288	1285	968	955
1H-Indene, 1-ethylidene-	2471-83-2	101239	-	HC	polycyclic aromatic HC	1448	0.535	141	1289	NA	821	932
Ethanone, 1-(4-ethylphenyl)-	93-55-0	77673	X	Oxy	ketone	1448	0.73	133	1289	1295	888	809
2-Tridecene, (E)-	41446-58-6	2051379	-	HC	alkene	1454	0.305	56	1292	1297	913	911
Bicyclo[4.4.1]undeca-1,3,5,7,9-pentaene	2443-46-1	114850	-	HC	polycyclic aromatic HC	1454	0.495	141	1292	NA	778	857
2-Undecanone	112-12-9	189842	X	Oxy	ketone	1454	0.625	58	1292	1292	899	820
Hydroquinone	123-31-9	1538373	G3	Oxy	quinone	1460	0.545	110	1296	NA	915	885

Continued on next page

Table S3. Tentatively identified compounds from the particulate phase fraction of mainstream tobacco smoke (Continued)

Tentative Identification	CAS	Peak Area	Health Effects	Class	Subclass	$^{1}t_R$ (s)	$^{2}t_R$ (s)	Quant Mass	Exp. RI*	Lib. RI*	Reverse†	Similarity‡
2-Tridecene, (Z)-	41446-59-7	26349	-	HC	alkene	1462	0.295	57	1297	1302	893	817
Naphthalene, 1,2,3,4-tetrahydro-1,8-dimethyl-	2219-78-5	123665	-	HC	polycyclic aromatic HC	1464	0.37	145	1298	1318	851	831
Naphthalene, 1,2,3,4-tetrahydro-5-methyl-	25419-33-4	44384	-	HC	polycyclic aromatic HC	1464	0.415	118	1298	1279	854	803
2-Ethyl-4,5-dimethylphenol	2809-64-5	311257	-	Oxy	alcohol	1464	0.43	135	1298	1300	909	835
Tridecane	629-50-5	2696030	-	HC	alkane	1466	0.29	57	1299	1300	963	951
Indole	120-72-9	4495705	-	Nit	aza-arene	1470	0.64	117	1302	1303	937	936
2-Allylphenol	4360-47-8	212451	-	Oxy	alcohol	1472	0.48	134	1303	NA	836	800
Cinnamonitrile	1745-81-9	533463	X	Nit	nitrile	1472	0.96	129	1303	1301	925	908
3-Tridecene, (E)-	41446-57-5	314144	-	HC	alkene	1474	0.29	69	1304	NA	921	904
Naphthalene, 2-methyl-	91-57-6	2312506	-	HC	polycyclic aromatic HC	1476	0.54	142	1305	1305	956	930
1-Ethyl-1,2,3,4-tetrahydronaphthalene	13556-58-6	100871	-	HC	polycyclic aromatic HC	1480	0.405	131	1308	1309	861	797
Ethanone, 1-(2-hydroxy-5-methylphenyl)-	1450-72-2	127899	-	Oxy	ketone	1482	0.85	135	1309	1316	760	836
Quinoxaline, 2-methyl-	7239-23-8	121072	-	Nit	aza-arene	1484	0.54	144	1310	1304	909	875
3-Butyl-2-hydroxyl-2-cyclopenten-1-one	17496-14-9	63789	-	Oxy	ketone	1490	0.67	112	1314	NA	838	769
1H-Inden-1-one, 2,3-dihydro-2-methyl-	29798-72-9	618867	-	Oxy	ketone	1490	0.75	131	1314	NA	915	913
2-Methyl-6-propylphenol	3520-52-3	188644	-	Oxy	alcohol	1492	0.42	121	1315	1320	856	791
2,5-Diethylphenol	876-20-0	293612	-	Oxy	alcohol	1494	0.42	135	1316	NA	897	837
1H-Inden-1-one, 2,3-dihydro-3,3-dimethyl-	91-63-4	380673	M	Oxy	ketone	1496	0.465	145	1318	NA	774	766
Quinoline, 2-methyl-	77822-47-0	254318	-	Nit	aza-arene	1496	0.555	143	1318	1313	945	914

Continued on next page

Table S3. Tentatively identified compounds from the particulate phase fraction of mainstream tobacco smoke (Continued)

Tentative Identification	CAS	Peak Area	Health Effects	Class	Subclass	^1t_R (s)	^2t_R (s)	Quant Mass	Exp. RI*	Lib. RI*	Reverse†	Similarity‡
2-Methoxy-4-vinylphenol	26465-81-6	2531743	-	Oxy	ether	1496	0.62	135	1318	1317	943	938
2-Ethylhexyl butyrate	25415-84-3	331407	-	Oxy	ester	1500	0.48	71	1320	1321	897	867
Triasteranone	1903-34-0	352892	-	Oxy	ketone	1500	0.82	91	1320	NA	800	820
1-Buten-3-ynylbenzene	146276-26-8	100531	-	HC	monocyclic aromatic HC	1502	0.555	128	1321	NA	950	807
Naphthalene, 1-methyl-	90-12-0	1937024	-	HC	polycyclic aromatic HC	1504	0.53	142	1323	1315	936	941
1-Cyclohexyl-1-pentyne	NA	213071	-	HC	alicyclic HC	1506	0.32	93	1324	NA	754	824
Trinoranastreptene	35587-60-1	38109	-	HC	polycyclic aromatic HC	1508	0.4	145	1325	NA	922	753
1-Methylindan-2-one	86160-98-7	390441	-	Oxy	ketone	1508	0.84	131	1325	NA	906	901
Quinoline, 5-methyl-	7661-55-4	216317	M	Nit	aza-arene	1510	0.52	143	1326	NA	921	886
Naphthalene, 1,2,3,4-tetrahydro-1,4-dimethyl-	527-54-8	48500	-	HC	polycyclic aromatic HC	1512	0.395	118	1327	NA	852	764
Phenol, 3,4,5-trimethyl-	4175-54-6	132606	-	Oxy	alcohol	1512	0.505	121	1328	1331	854	887
2,3,5,6-Tetramethylbenzo-1,4-quinone	527-17-3	140508	M	Oxy	quinone	1514	0.72	121	1329	1326	910	844
1H-Inden-5-ol, 2,3-dihydro-	1470-94-6	114802	X	Oxy	alcohol	1516	0.485	133	1330	1335	873	819
1-Isoprenyl-2-isopropylbenzene	24524-55-8	163351	-	HC	monocyclic aromatic HC	1520	0.39	145	1332	NA	867	822
Benzene, [(1-methylethylidene)cyclopropyl]-, (R)-	5557-93-7	155928	-	HC	monocyclic aromatic HC	1520	0.42	143	1332	NA	771	884
Phenol, 2-(1-methylpropyl)-	88-69-7	227832	-	Oxy	alcohol	1522	0.425	121	1334	NA	770	811

Continued on next page

Table S3. Tentatively identified compounds from the particulate phase fraction of mainstream tobacco smoke (Continued)

Tentative Identification	CAS	Peak Area	Health Effects	Class	Subclass	^1t_R (s)	^2t_R (s)	Quant Mass	Exp. RI*	Lib. RI*	Reverse†	Similarity‡
2-Methyl-7-exo-vinylbicyclo[4.2.0]oct-1(2)-ene	107914-89-6	48110	-	HC	polycyclic aromatic HC	1522	0.735	105	1334	NA	843	752
Benzopyrimidine, 3,4-dihydro-	1904-64-9	88191	-	Het	pyrimidine	1522	0.76	131	1334	NA	908	770
1-(3-Methylphenyl)4-methyl-3-pentene	51082-26-9	312150	-	HC	monocyclic aromatic HC	1524	0.36	105	1335	NA	871	851
(3E)-3-Methyl-4-phenyl-3-buten-2-one	1901-26-4	339783	X	Oxy	ketone	1526	0.49	145	1336	NA	818	806
Phosphonic acid, ethyl-, diethyl ester	78-38-6	86736	X	Misc	phosphonate	1526	1.185	54	1337	NA	999	901
1H-Inden-1-one, 2,3,4,5,6,7-hexahydro-	22118-00-9	52609	-	Oxy	ketone	1528	0.925	136	1338	NA	916	830
Pyrrolo[1,2-a]pyrazine, 3-methyl-	102-76-1	294069	-	Het	pyrazine	1530	0.765	132	1339	NA	906	864
Triacetin	64608-61-3	33719502	-	Oxy	ester	1530	1.17	103	1339	1339	938	935
Isoquinoline, 3-methyl-	1125-80-0	93343	M	Nit	aza-arene	1534	0.57	143	1341	NA	750	815
1-Methyl-4-(1-pentyn-1-yl)benzene	NA	192889	M	HC	monocyclic aromatic HC	1542	0.43	129	1346	NA	816	845
Benzylidenemalonaldehyde	825-54-7	165838	-	Oxy	aldehyde	1542	0.52	132	1346	NA	856	874
1-Naphthalenol, 4-methyl-	10240-08-1	69143	-	Oxy	alcohol	1544	0.505	157	1347	NA	841	817
11-Tridecyn-1-ol	33925-75-6	76306	-	Oxy	alcohol	1546	0.305	68	1348	NA	847	776
Benzaldehyde, ethyl-	53951-50-1	99699	-	Oxy	aldehyde	1552	0.495	134	1352	NA	807	837
Phenol, 2,6-dimethoxy-	91-10-1	851551	-	Oxy	ether	1552	0.71	154	1352	1353	928	926
Nicotine	54-11-5	26793591	M	Het	pyridine	1560	0.575	133	1357	1360	948	948
Naphthalene, 1,2,3,4-tetrahydro-1-propyl-	25586-57-6	192730	-	HC	polycyclic aromatic HC	1562	0.375	131	1358	NA	859	828

Continued on next page

Table S3. Tentatively identified compounds from the particulate phase fraction of mainstream tobacco smoke (Continued)

Tentative Identification	CAS	Peak Area	Health Effects	Class	Subclass	$^{1}t_R$ (s)	$^{2}t_R$ (s)	Quant Mass	Exp. RI*	Lib. RI*	Reverse†	Similarity‡
Benzene, 2-heptenyl-, (Z)-	54725-18-7	59911	-	HC	monocyclic aromatic HC	1562	0.385	104	1358	NA	805	754
Phenol, 2-methoxy-3-(2-propenyl)-	66324-83-2	101576	-	Oxy	ether	1562	0.565	164	1358	1362	861	859
7-Methylindan-1-one	39627-61-7	330314	-	Oxy	ketone	1564	0.68	146	1360	NA	880	894
Solanone	54868-48-3	4316592	-	Oxy	ketone	1566	0.64	93	1361	NA	934	919
Naphthalene, 1,2-dihydro-1,1,6-trimethyl-	87791-00-2	328122	-	HC	polycyclic aromatic HC	1570	0.39	142	1363	1364	906	885
Phenol, 2-(1,1-dimethylethyl)-5-methyl-	30364-38-6	68974	-	Oxy	alcohol	1570	0.4	149	1363	1365	908	773
Tridecane, 2-methyl-	6968-35-0	172858	-	HC	monocyclic aromatic HC	1572	0.29	57	1364	1364	899	854
Naphthalene, 1,2,3,4-tetrahydro-1,1,6-trimethyl-	1007-91-6	365322	-	HC	polycyclic aromatic HC	1572	0.38	159	1364	NA	924	905
Benzene, (1,1-dimethyl-2-butynyl)-	1560-96-9	284177	-	HC	monocyclic aromatic HC	1572	0.42	143	1365	NA	803	837
7-Hydroxy-1-indanone	475-03-6	101062	-	Oxy	ketone	1572	0.74	148	1365	NA	855	825
Quinoline, 8-methyl-	611-32-5	221459	M	Nit	aza-arene	1574	0.625	143	1366	NA	935	907
Benzene, 1,3-hexadienyl-	1667-01-2	152436	-	HC	monocyclic aromatic HC	1576	0.46	143	1367	NA	787	815
Ethanone, 1-(2,4,6-trimethylphenyl)-	41635-77-2	47589	-	Oxy	ketone	1576	0.695	147	1367	NA	875	753
Benzene, heptyl-	1078-71-3	289211	-	HC	monocyclic aromatic HC	1578	0.37	92	1368	1368	858	815
Phenol, 2-methoxy-4-propyl-	2785-87-7	143665	X	Oxy	ether	1578	0.58	137	1368	1369	838	790

Continued on next page

Table S3. Tentatively identified compounds from the particulate phase fraction of mainstream tobacco smoke (Continued)

Tentative Identification	CAS	Peak Area	Health Effects	Class	Subclass	^1t_R (s)	^2t_R (s)	Quant Mass	Exp. RI*	Lib. RI*	Reverse†	Similarity‡
Tridecane, 3-methyl-	6418-41-3	134896	-	HC	alkane	1582	0.29	57	1371	1371	796	762
1H-Indene, 1,1,3-trimethyl-	2177-45-9	353320	-	HC	polycyclic aromatic HC	1582	0.425	143	1371	NA	829	835
Phenol, 2-(2-methyl-2-propenyl)-	612-58-8	55531	M	Oxy	alcohol	1584	0.48	148	1372	NA	834	789
Quinoline, 3-methyl-	20944-88-1	105272	-	Nit	aza-arene	1584	0.645	143	1372	1361	870	780
1H-Indole, 2-methyl-	95-20-5	175282	X	Nit	aza-arene	1586	0.67	130	1373	1373	917	844
1,3-Dimethyl -1H-indole	875-30-9	206229	-	Nit	aza-arene	1588	0.62	144	1375	1383	906	883
Farnesane	3891-98-3	1164068	-	HC	sesquiterpene	1590	0.3	71	1376	1376	938	929
2,3,5,6-Tetramethylacetophenone	2142-79-2	271421	-	Oxy	ketone	1592	0.62	161	1377	NA	836	827
(E)-1-(2,3,6-trimethylphenyl)buta-1,3-diene (TPB, 1)	18190-44-8	96034	-	HC	monocyclic aromatic HC	1598	0.395	157	1381	NA	820	757
Ethanone, 1-[4-(1-methylethenyl)phenyl]-	5359-046	289429	-	Oxy	ketone	1598	0.605	145	1381	NA	782	790
N-[2-Hydroxyethyl]succinimide	NA	79013	-	Nit	imide	1598	1.025	100	1381	NA	864	783
beta-Elemene	33880-83-0	39138	-	HC	sesquiterpene	1600	0.345	147	1382	1384	806	783
(1-Methylpenta-1,3-dienyl)benzene	116669-49-9	1333540	-	HC	monocyclic aromatic HC	1602	0.45	143	1383	NA	870	875
Quinoline, 2,7-dimethyl-	49826-53-1	94047	-	Nit	aza-arene	1606	0.47	157	1386	1392	772	829
beta-Damascenone	93-37-8	357561	-	Oxy	ketone	1606	0.625	69	1386	1385	804	771
1(2H)-Naphthalenone, 3,4-dihydro-	23726-93-4	61730	-	Oxy	ketone	1606	0.78	118	1386	NA	870	801
Bicyclo[3.2.1]oct-2-ene, 3-methyl-4-methylene-	529-34-0	212853	-	HC	alicyclic HC	1606	1.055	91	1386	NA	820	764

Continued on next page

Table S3. Tentatively identified compounds from the particulate phase fraction of mainstream tobacco smoke (Continued)

Tentative Identification	CAS	Peak Area	Health Effects	Class	Subclass	$^{1}t_R$ (s)	$^{2}t_R$ (s)	Quant Mass	Exp. RI*	Lib. RI*	Reverse†	Similarity‡
Benzene, (1,2,3-trimethyl-2-cyclopropen-1-yl)-	6393-13-1	318725	-	HC	monocyclic aromatic HC	1610	0.46	143	1388	NA	849	874
Methyl 5-oxo-2-pyrrolidinecarboxylate	54571-66-3	704779	-	Het	pyrrole	1610	1.495	84	1389	1391	912	826
Biphenyl	92-52-4	647857	G2A	HC	polycyclic aromatic HC	1612	0.47	154	1389	1391	955	944
1H-Pyrrole, 1-(4-methylphenyl)-	491-35-0	26567	M	Het	pyrrole	1614	0.51	157	1390	NA	847	755
Quinoline, 4-methyl-	82700-43-4	187270	-	Nit	aza-arene	1614	0.65	143	1391	1391	943	884
1-Tetradecene	1120-36-1	1246422	-	HC	alkene	1616	0.29	69	1392	1392	951	943
3-Dodecen-1-al	67886-53-7	135487	-	Oxy	aldehyde	1622	0.3	70	1395	1399	808	756
1H-Indole, 3-methyl-	83-34-1	4845616	-	Nit	aza-arene	1622	0.6	130	1395	1395	944	944
Pyrrol[1,2-a]pyrazine, 1,4-dimethyl-	64608-63-5	60767	-	Het	pyrazine	1626	0.62	146	1398	NA	880	795
1H-Indene, 2,3-dihydro-1,1,3-trimethyl-	2613-76-5	110883	-	HC	polycyclic aromatic HC	1628	0.375	145	1399	NA	908	857
Tetradecane	629-59-4	2645724	-	HC	alkane	1630	0.28	57	1400	1400	958	954
1H-Indene, 3-ethenyl-2,3-dihydro-1,1-dimethyl-	612-60-2	118898	M	HC	polycyclic aromatic HC	1634	0.415	142	1403	NA	823	799
5-methyl-1H-Indole	614-96-0	571301	-	Nit	aza-arene	1634	0.62	130	1403	1398	863	884
Quinoline, 7-methyl-	53909-98-1	94552	-	Nit	aza-arene	1634	0.68	143	1403	1355	861	788
3-Tetradecene, (Z)-	41446-67-7	148206	-	HC	alkene	1636	0.28	56	1404	1385	928	922
2-Methylbiphenyl	54484-71-8	48017	-	HC	polycyclic aromatic HC	1638	0.4	168	1406	1404	876	815
3,3,6-trimethyl-1-Indanone	1127-76-0	226119	-	Oxy	ketone	1638	0.425	159	1406	NA	779	759

Continued on next page

Table S3. Tentatively identified compounds from the particulate phase fraction of mainstream tobacco smoke (Continued)

Tentative Identification	CAS	Peak Area	Health Effects	Class	Subclass	$^{1}t_R$ (s)	$^{2}t_R$ (s)	Quant Mass	Exp. RI*	Lib. RI*	Reverse†	Similarity‡
1-Ethylnaphthalene	643-58-3	1106695	X	HC	polycyclic aromatic HC	1638	0.5	141	1406	1406	936	932
1-Phenylpyrrolidine	4096-21-3	70981	-	Het	pyrrole	1640	0.525	146	1407	NA	815	893
trans-beta-Caryophyllene	621-59-0	598182	M	HC	sesquiterpene	1642	0.33	93	1408	1406	844	804
Isovanillin	87-44-5	243745	-	Oxy	aldehyde	1642	0.895	151	1408	1401	915	875
Naphthalene, 2,6-dimethyl-	581-42-0	596589	-	HC	polycyclic aromatic HC	1656	0.52	156	1417	1416	944	932
Naphthalene, 1,7-dimethyl-	24644-78-8	310345	-	HC	polycyclic aromatic HC	1660	0.52	156	1420	1419	949	939
4-methyl-1-Indanone	575-37-1	763569	-	Oxy	ketone	1660	0.855	117	1420	1444	911	908
1(3H)-Isobenzofuranone, 5-methyl-	54120-64-8	101191	-	Het	furan	1668	1.065	119	1425	NA	789	853
Germacrene A	28387-44-2	231769	-	HC	sesquiterpene	1674	0.375	121	1429	NA	832	852
7-Methyl-1-naphthol	6939-33-9	104350	-	Oxy	alcohol	1676	0.745	158	1430	NA	866	815
Naphthalene, 1,5-dimethyl-	26452-86-8	914918	-	HC	polycyclic aromatic HC	1678	0.52	156	1432	1428	948	942
Benzene, (2-methyl-1-methylenebutyl)-	571-61-9	136225	-	HC	monocyclic aromatic HC	1678	0.805	132	1432	NA	780	760
cis-beta-Caryophyllene	550-44-7	590520	-	HC	sesquiterpene	1680	0.32	69	1433	1431	855	858
N-methylphthalimide	118-65-0	136747	-	Nit	imide	1680	0.89	161	1433	1425	924	902
Bicyclo[3.1.1]heptane, 2,6,6-trimethyl-3-(2-propenyl)-, (1á,2á,3á,5á)-	532-12-7	674916	M	HC	alicyclic HC	1684	0.315	81	1435	NA	784	764
Pyridine, 3-(3,4-dihydro-2H-pyrrol-5-yl)-	50746-55-9	2156822	-	Het	pyridine	1684	0.7	118	1436	1427	935	934
1,5,9-Decatriene, 2,3,5,8-tetramethyl-	230646-72-7	442246	-	HC	alkene	1686	0.295	69	1437	NA	804	802

Continued on next page

Table S3. Tentatively identified compounds from the particulate phase fraction of mainstream tobacco smoke (Continued)

Tentative Identification	CAS	Peak Area	Health Effects	Class	Subclass	$^{1}t_R$ (s)	$^{2}t_R$ (s)	Quant Mass	Exp. RI*	Lib. RI*	Reverse†	Similarity‡
Naphthalene, 1,6-dimethyl-	575-43-9	937384	X	HC	polycyclic aromatic HC	1686	0.515	156	1437	1428	895	925
Bicyclogermacrene	24703-35-3	335635	-	HC	sesquiterpene	1688	0.355	121	1438	NA	887	885
Naphthalene, 1,2,3,4-tetrahydro-1,1-dimethyl-	1985-59-7	844734	-	HC	polycyclic aromatic HC	1690	0.75	145	1439	NA	783	793
Benzylbenzene	101-81-5	45621	X	HC	polycyclic aromatic HC	1692	0.435	168	1441	1437	859	783
3-Tetradecene, (E)-	41446-68-8	89482	-	HC	alkene	1698	0.295	69	1444	NA	833	782
2,7-Ethanonaphth[2,3-b]oxirene, 1a,2,7,7a-tetrahydro-, (1aá,2á,7á,7aá)-	16017-24-6	97925	-	Oxy	epoxide	1700	0.405	143	1446	NA	850	773
2-Propyn-1-ol, 3-(4-methylphenyl)-	54515-76-3	290267	-	Oxy	alcohol	1700	0.495	131	1446	NA	837	806
2-Indolinone, 1-methyl-	61-70-1	60539	X	Nit	aza-arene	1700	0.87	118	1446	NA	854	786
trans-beta-Farnesene	07/05/6638	448069	-	HC	sesquiterpene	1702	0.32	93	1447	1446	794	812
3,5-Dimethoxy-4-hydroxytoluene	18794-84-8	131717	-	Oxy	ether	1702	0.68	168	1447	1447	798	757
Gernanylacetone	3796-70-1	2420019	-	Oxy	ketone	1704	0.57	43	1448	1451	928	918
cis-beta-Farnesene	91-64-5	973460	M	HC	sesquiterpene	1712	0.32	69	1454	1452	875	878
Coumarin	28973-97-9	182123	-	Het	pyran	1712	1.12	118	1454	1456	886	824
Naphthalene, 1,8-dimethyl-	569-41-5	161692	-	HC	polycyclic aromatic HC	1714	0.505	156	1455	1450	897	933
Phenol, 2-methoxy-4-(1-propenyl)-, (Z)-	5912-86-7	783503	X	Oxy	ether	1714	0.595	164	1455	1441	923	922
1,5-Cyclooctadiene, 3-(1-methyl-2-propenyl)-	16538-88-8	507392	-	HC	alicyclic HC	1718	0.32	93	1457	NA	817	787

Continued on next page

Table S3. Tentatively identified compounds from the particulate phase fraction of mainstream tobacco smoke (Continued)

Tentative Identification	CAS	Peak Area	Health Effects	Class	Subclass	$^{1}t_R$ (s)	$^{2}t_R$ (s)	Quant Mass	Exp. RI*	Lib. RI*	Reverse†	Similarity‡
1-Tridecanol	208-96-8	80111	M	Oxy	alcohol	1730	0.295	69	1465	NA	800	772
Quinoline, 2,4-dimethyl-	1198-37-4	47591	-	Nit	aza-arene	1730	0.52	156	1465	1472	779	840
Acenaphthylene	112-70-9	379721	X	HC	polycyclic aromatic HC	1730	0.59	152	1465	1466	950	903
Naphthalene, 1,2,3,5,8,8a-hexahydro-	62690-65-7	307873	-	HC	polycyclic aromatic HC	1732	0.93	134	1467	NA	797	821
1,2-Dimethylnaphthalene	573-98-8	285679	-	HC	polycyclic aromatic HC	1734	0.525	141	1468	1462	850	877
Pyridine, 2-phenyl-	1008-89-5	183106	M	Het	pyridine	1736	0.57	154	1469	1466	833	759
2,6-dimethyl-1H-Indole	5649-36-5	119974	-	Nit	aza-arene	1736	0.61	144	1469	NA	841	801
Tetradecane, 3-methyl-	18435-22-8	54704	-	HC	alkane	1738	0.28	71	1470	1470	839	756
Naphthalene, 1,2-dihydro-7-methyl-1-(1-methylethenyl)-	NA	123546	-	HC	polycyclic aromatic HC	1738	0.41	141	1470	NA	892	762
Alloaromadendrene	2189-60-8	23610	-	HC	sesquiterpene	1740	0.35	161	1472	NA	780	754
Benzene, octyl-	25246-27-9	313747	-	HC	monocyclic aromatic HC	1740	0.36	92	1472	1472	876	813
alpha-Humulene	6753-98-6	1277858	-	HC	sesquiterpene	1742	0.32	41	1473	1472	833	836
Pyridine, 4-phenyl-	15158-36-8	504920	-	Het	pyridine	1744	0.61	155	1474	1471	915	905
4-Methylphthalaldehyde	937-30-4	193539	-	Oxy	aldehyde	1744	1.215	119	1475	NA	893	833
1H-Inden-1-one, 2,3-dihydro-5,7-dimethyl-	6682-69-5	104103	-	Oxy	ketone	1752	0.66	160	1480	NA	885	850
Norsolanadione	NA	2661384	-	Oxy	ketone	1754	1.375	43	1481	NA	913	910
Pyridine, 2-(phenylmethyl)-	1203-08-3	88641	-	Het	pyridine	1756	0.5	168	1482	1482	904	835
4-(2,6,6-Trimethylcyclohexa-1,3-dienyl)but-3-en-2-one	101-82-6	473512	X	Oxy	ketone	1756	0.72	43	1482	NA	887	881

Continued on next page

Table S3. Tentatively identified compounds from the particulate phase fraction of mainstream tobacco smoke (Continued)

Tentative Identification	CAS	Peak Area	Health Effects	Class	Subclass	^1t_R (s)	^2t_R (s)	Quant Mass	Exp. RI*	Lib. RI*	Reverse†	Similarity‡
Naphthalene, 1,2-dihydro-2,5,8-trimethyl-	30316-23-5	113674	-	HC	polycyclic aromatic HC	1758	0.44	157	1483	NA	827	810
cis-alpha-Farnesene	79756-89-1	885095	-	HC	sesquiterpene	1760	0.305	93	1485	1484	851	863
trans-beta-Ionone	26560-14-5	43275	-	Oxy	ketone	1760	0.715	177	1485	1485	826	796
alpha-Curcumene	644-30-4	109976	-	HC	sesquiterpene	1762	0.34	132	1486	1486	909	851
3-(1-methyl-1H-pyrrol-2-yl)Pyridine	487-19-4	1779704	X	Het	pyridine	1762	0.67	158	1486	1488	905	891
Naphthalene, 1,2-dihydro-1,4,6-trimethyl-	55682-80-9	158080	-	HC	polycyclic aromatic HC	1764	0.43	157	1487	NA	897	870
5,6-dimethyl-1-Indanone	16440-97-4	140542	-	Oxy	ketone	1766	0.675	160	1489	NA	873	884
Benzenamine, N-(1-methyl-2-propynyl)-	53832-62-5	1222978	-	Nit	amine	1768	0.565	130	1490	NA	814	823
1-Pentadecene	13360-61-7	1143841	-	HC	alkene	1770	0.29	57	1491	1491	925	926
5-propionyl-2,3-dihydro-1H-Pyrrolizine	79-77-6	108010	X	Het	pyrrole	1770	0.585	134	1491	NA	931	796
Apocynin	498-02-2	167571	M	Oxy	ketone	1772	0.885	151	1493	1491	895	815
1,1’-Biphenyl, 3-methyl-	643-93-6	306974	-	HC	polycyclic aromatic HC	1776	0.46	168	1495	1490	906	919
1,4-Naphthalenediol	571-60-8	49222	M	Oxy	alcohol	1778	0.945	160	1497	NA	907	825
Acenaphthene	83-32-9	247895	G3	HC	polycyclic aromatic HC	1780	0.51	153	1498	1494	945	922
1H-Inden-1-one, 2,3-dihydro-3,4,7-trimethyl-	35322-84-0	77416	-	Oxy	ketone	1780	0.59	159	1498	NA	803	770
Pentadecane	91-55-4	4339793	M	HC	alkane	1782	0.28	57	1499	1500	950	944
2,3-dimethyl-1H-Indole	629-62-9	688844	-	Nit	aza-arene	1782	0.64	144	1499	1499	925	922

Continued on next page

Table S3. Tentatively identified compounds from the particulate phase fraction of mainstream tobacco smoke (Continued)

Tentative Identification	CAS	Peak Area	Health Effects	Class	Subclass	$^{1}t_R$ (s)	$^{2}t_R$ (s)	Quant Mass	Exp. RI*	Lib. RI*	Reverse†	Similarity‡
Naphthalene, 1-propyl-	2765-18-6	126961	-	HC	polycyclic aromatic HC	1784	0.49	141	1500	1504	867	756
5',6',7',8'-Tetrahydro-2'-acetonaphthone	774-55-0	80865	-	Oxy	ketone	1786	0.615	159	1502	NA	793	840
alpha-Farnesene	4506-36-9	965150	-	HC	sesquiterpene	1790	0.32	69	1504	1504	936	934
1,5,8-Trimethyl-1,2-dihydronapthalene	502-61-4	203364	-	HC	polycyclic aromatic HC	1790	0.425	157	1505	NA	775	782
Anthracene, 1,2,3,4,5,6-hexahydro-	613-46-7	390324	M	HC	polycyclic aromatic HC	1796	0.425	128	1509	NA	800	795
Naphthalene-2-carbonitrile	6109-22-4	129763	-	Nit	nitrile	1796	0.86	153	1509	1497	944	839
beta-Bisabolene	495-61-4	729409	-	HC	sesquiterpene	1802	0.32	69	1513	1513	849	850
3-methyl-2H-Chromen-2-one	2445-82-1	237011	X	Het	pyran	1806	1	131	1516	NA	917	874
(1,1'-Biphenyl)-2-amine	90-41-5	65702	M	Nit	amine	1808	0.595	168	1517	NA	837	758
13-Tetradece-11-yn-1-ol	NA	48192	-	Oxy	alcohol	1822	0.305	54	1527	NA	807	776
1H-Pyrazole, 3,5-dimethyl-1-phenyl-	1131-16-4	614183	-	Het	pyrazole	1822	0.575	171	1527	NA	780	789
alpha-Selinene	473-13-2	508576	-	HC	sesquiterpene	1824	0.365	93	1528	NA	845	769
1-Naphthol	90-15-3	95804	X	Oxy	alcohol	1826	0.55	144	1530	1525	875	758
Bibenzyl	2503-46-0	392078	-	HC	polycyclic aromatic HC	1828	0.44	91	1531	1528	931	851
Guiacylacetone	103-29-7	328662	-	Oxy	ketone	1828	0.975	137	1531	1532	886	759
Dibenzofuran	132-64-9	316528	M	Het	furan	1830	0.55	168	1532	1532	868	843
Naphthalene, 1,4,5-trimethyl-	1971-46-6	244462	-	HC	polycyclic aromatic HC	1836	0.5	155	1537	1536	935	929
1H-Indole, 1,2,3-trimethyl-	2131-41-1	57556	-	Nit	aza-arene	1836	0.63	158	1537	NA	894	791
1-Phenyl-1-octyne	16967-02-5	626540	-	HC	alkyne	1846	0.41	143	1543	NA	759	759

Continued on next page

Table S3. Tentatively identified compounds from the particulate phase fraction of mainstream tobacco smoke (Continued)

Tentative Identification	CAS	Peak Area	Health Effects	Class	Subclass	$^{1}t_R$ (s)	$^{2}t_R$ (s)	Quant Mass	Exp. RI*	Lib. RI*	Reverse†	Similarity‡
2(4H)-Benzofuranone, 5,6,7,7a-tetrahydro-4,4,7a-trimethyl-, (R)-	17092-92-1	286088	-	Oxy	ketone	1846	1.685	111	1544	1538	895	879
Naphthalene, 1,6,7-trimethyl-	2245-38-7	689027	-	HC	polycyclic aromatic HC	1848	0.495	155	1545	1544	848	846
2,3'-Dipyridyl	581-50-0	2195517	X	Het	pyridine	1850	0.715	155	1546	1536	915	900
Aromadendrene	27776-01-8	353319	-	HC	sesquiterpene	1854	0.35	105	1549	NA	854	833
Benzyltoluene	74685-33-9	28238	-	HC	polycyclic aromatic HC	1854	0.41	167	1549	NA	844	792
4-Hexadecen-6-yne, (Z)-	21391-99-1	103631	-	HC	alkyne	1856	0.335	69	1550	NA	780	755
alpha-Calacorene	6813-21-4	133349	-	HC	sesquiterpene	1858	0.39	157	1552	NA	876	789
Selina-3,7(11)-diene	295-48-7	31598	-	HC	sesquiterpene	1862	0.35	204	1554	1551	901	894
Cyclopentadecane	827-60-1	111428	-	HC	alicyclic HC	1864	0.295	56	1556	NA	879	861
Naphthalene, 2,3,6-trimethyl-	2489-86-3	340810	M	HC	polycyclic aromatic HC	1868	0.49	155	1559	1550	901	914
Naphthalene, 1-(2-propenyl)-	156747-45-4	55728	-	HC	polycyclic aromatic HC	1872	0.525	168	1562	NA	862	762
Isolongifolene, 4,5,9,10-dehydro-	28343-22-8	173541	-	HC	sesquiterpene	1876	0.395	157	1564	NA	807	793
Phenol, 4-ethenyl-2,6-dimethoxy-	2882-96-4	364961	-	Oxy	ether	1878	0.67	180	1566	1568	908	875
Pentadecane, 3-methyl-	27505-78-8	124343	-	HC	alkane	1884	0.29	57	1570	1570	796	839
Naphthalene, 1-methyl-7-(1-methylethyl)-	490-65-3	305851	-	HC	polycyclic aromatic HC	1884	0.48	169	1570	1578	895	889
2,3,7-trimethyl-1H-Indole	51766-65-5	61397	-	Nit	aza-arene	1884	0.645	158	1570	NA	881	764

Continued on next page

Table S3. Tentatively identified compounds from the particulate phase fraction of mainstream tobacco smoke (Continued)

Tentative Identification	CAS	Peak Area	Health Effects	Class	Subclass	^1t_R (s)	^2t_R (s)	Quant Mass	Exp. RI*	Lib. RI*	Reverse†	Similarity‡
gamma-Dehydro-ar-himachalene	62235-06-7	116474	-	HC	sesquiterpene	1888	0.405	157	1573	NA	809	756
(3E,7E)-4,8,12-Trimethyltrideca-1,3,7,11-tetraene	1081-77-2	684188	-	HC	alkene	1890	0.32	69	1574	1573	889	889
Benzene, nonyl-	5037-60-5	327963	-	HC	monocyclic aromatic HC	1892	0.36	92	1575	1576	939	885
1H-Phenalene	203-80-5	312105	-	HC	polycyclic aromatic HC	1902	0.58	165	1582	NA	888	852
1H-Inden-1-one, 2,3-dihydro-4,7-dimethyl-	212394-95-1	137442	-	Oxy	ketone	1902	0.85	117	1583	NA	889	849
Eudesma-1,4(15),11-triene	229-934-9	46115	X	HC	sesquiterpene	1908	0.36	108	1586	NA	804	752
2,2,4-Trimethyl-1,3-pentanediol diisobutyrate	525-40-6	426073	-	Oxy	ester	1912	0.635	71	1589	1588	846	804
1H-Pyrido[3,4-b]indole, 2,3,4,9-tetrahydro-1-methyl-	629-73-2	133515	-	Nit	aza-arene	1914	0.545	171	1591	NA	796	786
1-Hexadecene	14002-89-2	483957	-	HC	alkene	1916	0.29	83	1592	1592	929	930
2H-1-Benzopyran-2-one, 4,6-dimethyl-	21296-92-4	56117	M	Oxy	ketone	1920	0.87	145	1595	NA	796	855
Hexadecane	6627-88-9	1189451	-	HC	alkane	1926	0.28	57	1599	1600	933	929
1H-Indole, 2,3,5-trimethyl-	544-76-3	161784	X	Nit	aza-arene	1926	0.6	158	1599	1593	922	838
Methoxyeugenol	86-73-7	34186	G3	Oxy	ether	1926	0.62	194	1599	1602	850	798
Fluorene	613-33-2	869220	-	HC	polycyclic aromatic HC	1928	0.54	166	1600	1606	921	921
4,4'-Dimethylbiphenyl	1855-47-6	120985	-	HC	polycyclic aromatic HC	1942	0.455	182	1611	1608	904	869

Continued on next page

Table S3. Tentatively identified compounds from the particulate phase fraction of mainstream tobacco smoke (Continued)

Tentative Identification	CAS	Peak Area	Health Effects	Class	Subclass	^1t_R (s)	^2t_R (s)	Quant Mass	Exp. RI*	Lib. RI*	Reverse†	Similarity‡
1-Isopropenylnaphthalene	2523-37-7	138619	-	HC	polycyclic aromatic HC	1948	0.515	153	1615	NA	831	815
9-methyl-9H-Fluorene	4854-85-7	225786	-	HC	polycyclic aromatic HC	1954	0.51	165	1620	1579	904	895
trans-p-Dimethylaminocinnamonitrile	92-83-1	218938	-	Nit	nitrile	1956	0.655	171	1621	NA	801	763
9H-Xanthene	119-61-9	88395	G2B	Het	pyran	1978	0.515	181	1638	1635	913	769
1,4,5,8-Tetramethylnaphthalene	2717-39-7	134399	-	HC	polycyclic aromatic HC	1984	0.475	169	1642	NA	810	846
Benzophenone	14002-90-5	79281	-	Oxy	ketone	1984	0.665	105	1642	1644	937	769
4,7-Dimethylcoumarin	3892-00-0	107391	-	Het	pyran	1984	0.975	145	1643	NA	893	815
Pentadecane, 2,6,10-trimethyl-	34318-21-3	620107	-	HC	alkane	1990	0.29	57	1647	1649	901	901
1,4-Naphthalenedione, 2,3-dimethyl-	2197-57-1	84109	X	Oxy	ketone	1996	0.735	186	1651	NA	863	837
2-Cyclohexen-1-one, 4-(3-hydroxy-1-butenyl)-3,5,5-trimethyl-	610-35-5	840376	-	Oxy	ketone	1996	1.16	108	1652	1648	899	890
4-Hydroxyphthalate	229-95-8	174422	-	Oxy	ester	2000	0.545	181	1654	NA	905	821
6H-Dibenzo[b,d]-pyran	629-74-3	37063	-	Het	pyran	2012	0.54	181	1663	NA	793	875
1-Hexadecyne	16369-12-3	95723	-	HC	alkyne	2024	0.29	54	1672	NA	868	899
8-Heptadecene	NA	303858	-	HC	alkene	2034	0.29	69	1679	1679	878	887
Decylbenzene	104-72-3	154950	X	HC	monocyclic aromatic HC	2036	0.355	92	1681	1681	837	799
Propanedinitrile, 2-(2,2,3,4-tetramethyl-4-cyclopenten-1-ylidene)-	3031-15-0	202415	-	Nit	nitrile	2036	0.555	171	1681	NA	789	773

Continued on next page

Table S3. Tentatively identified compounds from the particulate phase fraction of mainstream tobacco smoke (Continued)

Tentative Identification	CAS	Peak Area	Health Effects	Class	Subclass	$^{1}t_R$ (s)	$^{2}t_R$ (s)	Quant Mass	Exp. RI*	Lib. RI*	Reverse†	Similarity‡
Naphthalene, 1,2,3,4-tetramethyl-	2040-10-0	66877	-	HC	polycyclic aromatic HC	2040	0.5	169	1684	NA	837	780
4'-tert-Butyl-2',6'-dimethylacetophenone	6765-39-5	191140	-	Oxy	ketone	2046	0.485	189	1688	NA	837	792
1-Heptadecene	2345-28-0	418702	-	HC	alkene	2052	0.29	57	1693	1693	925	933
2-Pentadecanone	629-78-7	125015	-	Oxy	ketone	2060	0.53	58	1699	1699	781	877
Heptadecane	20675-95-0	1230578	-	HC	alkane	2062	0.28	57	1700	1700	952	937
(E)-2,6-Dimethoxy-4-(prop-1-en-1-yl)phenol	1430-97-3	82807	-	Oxy	ether	2068	0.635	194	1705	1704	894	865
9H-Fluorene, 2-methyl-	36151-02-7	49239	-	HC	polycyclic aromatic HC	2074	0.565	180	1710	1720	822	770
Blumenol C	1556-99-6	36793	-	Oxy	alcohol	2078	1.175	108	1713	1713	870	784
4-methyl-9H-fluorene	486-56-6	800705	X	HC	polycyclic aromatic HC	2084	0.525	165	1717	NA	891	895
Cotinine	62600-05-9	774111	-	Het	pyrrole	2084	1.34	98	1718	NA	938	930
Cedran-diol, 8S,14-	3910-35-8	621536	-	Oxy	alcohol	2086	0.59	43	1719	NA	788	768
1H-Indene, 2,3-dihydro-1,1,3-trimethyl-3-phenyl-	1730-37-6	72676	-	HC	polycyclic aromatic HC	2092	0.41	143	1723	1714	792	765
9H-Fluorene, 1-methyl-	4569-45-3	330566	M	HC	polycyclic aromatic HC	2094	0.55	165	1725	1719	953	926
9H-Fluorene, 9,9-dimethyl-	529-05-5	192044	X	HC	polycyclic aromatic HC	2102	0.5	179	1731	NA	881	824
Chamazulene	24875-94-3	86785	-	HC	polycyclic aromatic HC	2106	0.515	169	1734	1734	806	848
6'-Methyl-2'-acetonaphthone	2980-70-3	36967	-	Oxy	ketone	2114	0.765	169	1741	NA	882	787

Continued on next page

Table S3. Tentatively identified compounds from the particulate phase fraction of mainstream tobacco smoke (Continued)

Tentative Identification	CAS	Peak Area	Health Effects	Class	Subclass	$^{1}t_{R}$ (s)	$^{2}t_{R}$ (s)	Quant Mass	Exp. RI*	Lib. RI*	Reverse†	Similarity‡
Cyclopentane, 1,1'-(1,4-butandiyl)bis-	20071-09-4	74995	-	HC	alicyclic HC	2138	0.305	67	1759	NA	789	781
Benzene, 1,1'-(1,2-cyclobutanediyl)bis-, trans-	486-25-9	379014	X	HC	monocyclic aromatic HC	2140	0.44	104	1760	NA	885	869
9H-Fluroen-9-one	20490-42-0	99389	-	Oxy	ketone	2144	0.755	180	1764	1752	932	821
1,4-Naphthalenedione, 2,3,6-trimethyl-	7206-15-7	179303	-	Oxy	ketone	2164	0.73	200	1779	NA	893	878
(3E)-3-Octadecene	593-45-3	404442	-	HC	alkene	2184	0.29	55	1794	1795	900	904
Octadecane	18787-63-8	506640	X	HC	alkane	2192	0.28	57	1800	1800	936	928
2-Hexadecanone	85-01-8	170462	G3	Oxy	ketone	2192	0.515	58	1800	1800	929	895
Phenanthrene	37841-91-1	327195	M	HC	polycyclic aromatic HC	2200	0.61	178	1807	1797	942	935
Isovelleral	54878-25-0	69041	-	Oxy	aldehyde	2204	0.73	91	1810	NA	799	783
Solavetivone	120-12-7	54695	G3	Oxy	ketone	2214	0.93	108	1819	NA	919	849
Anthracene	4612-63-9	101304	-	HC	polycyclic aromatic HC	2216	0.61	178	1820	1817	928	835
9H-Fluorene, 2,3-dimethyl-	504-96-1	213165	-	HC	polycyclic aromatic HC	2234	0.535	179	1835	NA	803	907
Neophytadiene	17239-99-5	12532161	-	HC	diterpene	2240	0.33	68	1840	1840	938	925
Anthracene, 9,10-dihydro-9-methyl-	2437-93-6	63867	-	HC	polycyclic aromatic HC	2240	0.52	178	1840	NA	763	810
2-Phytene (trans)	629-89-0	764375	-	HC	alkene	2244	0.3	70	1843	1838	873	870
1-Octadecyne	502-69-2	38040	-	HC	alkyne	2244	0.31	123	1843	NA	803	787
2-Pentadecanone, 6,10,14-trimethyl-	2854-40-2	879160	-	Oxy	ketone	2244	0.53	58	1843	1844	892	857
Cyclo(L-prolyl-L-valine)	942-43-8	57422	-	Nit	amino acid	2262	1.31	70	1859	NA	856	766

Continued on next page

Table S3. Tentatively identified compounds from the particulate phase fraction of mainstream tobacco smoke (Continued)

Tentative Identification	CAS	Peak Area	Health Effects	Class	Subclass	$^{1}t_R$ (s)	$^{2}t_R$ (s)	Quant Mass	Exp. RI*	Lib. RI*	Reverse†	Similarity‡
Anthracene, 9,10-dihydro-2-methyl-	244-99-5	87506	-	HC	polycyclic aromatic HC	2268	0.525	179	1863	NA	841	780
4-Azafluorene	NA	99321	-	Nit	aza-arene	2274	0.675	167	1868	NA	888	777
3,7,11-Trimethyl-2,4-dodecadiene	67858-77-9	110144	-	HC	alkene	2288	0.31	82	1879	NA	785	769
4,8,12-Tetradecatrien-1-ol, 5,9,13-trimethyl-	18435-45-5	70755	-	Oxy	alcohol	2304	0.305	69	1892	NA	823	785
1-Nonadecene	629-92-5	107088	-	HC	alkene	2306	0.3	83	1894	1894	914	927
Nonadecane	NA	368271	-	HC	alkane	2314	0.28	57	1900	1900	911	934
3-hydroxy-Solavetivone	2922-51-2	74626	-	Oxy	ketone	2314	0.84	108	1901	NA	777	753
2-Heptadecanone	70901-63-2	245750	-	Oxy	ketone	2316	0.51	58	1902	1902	930	894
beta-Springene	01/11/7557	271655	-	HC	alkene	2330	0.32	69	1914	NA	872	863
trans,trans-Farnesyl acetone	832-69-9	596314	G3	Oxy	ketone	2330	0.515	43	1914	1914	885	883
Phenanthrene, 1-methyl-	112-39-0	32942	-	HC	polycyclic aromatic HC	2340	0.615	192	1924	1934	912	787
Hexadecanoic acid, methyl ester	613-12-7	479573	M	Oxy	ester	2344	0.41	74	1926	1926	945	892
Anthracene, 2-methyl-	610-48-0	75571	-	HC	polycyclic aromatic HC	2348	0.615	192	1929	1928	940	865
Anthracene, 1-methyl-	123-69-3	32834	X	HC	polycyclic aromatic HC	2362	0.615	192	1941	1935	793	905
Oxacycloheptadec-8-en-2-one	19943-27-2	155135	-	Oxy	ketone	2366	0.56	67	1945	1936	867	846
Cycro-(Pro-Pro)	1898-13-1	260070	-	Nit	amino acid	2378	1.23	70	1955	NA	925	837
(+)-Cembrene	77898-97-6	386081	-	HC	alicyclic HC	2380	0.38	91	1956	NA	864	830
alpha-Springene	74685-27-1	359823	-	HC	alkene	2390	0.32	69	1965	1969	882	871

Continued on next page

Table S3. Tentatively identified compounds from the particulate phase fraction of mainstream tobacco smoke (Continued)

Tentative Identification	CAS	Peak Area	Health Effects	Class	Subclass	^1t_R (s)	^2t_R (s)	Quant Mass	Exp. RI*	Lib. RI*	Reverse†	Similarity‡
3-Eicosene, (E)-	112-95-8	89467	-	HC	alkene	2424	0.3	57	1994	NA	877	900
Eicosane	5508-58-7	198869	M	HC	alkane	2432	0.29	57	2000	2000	917	904
Andrographolide	29837-07-8	72113	-	Oxy	alcohol	2472	0.44	91	2036	NA	834	784
cis-alpha-Bisabolene epoxide	948-67-4	766502	-	Oxy	epoxide	2534	0.505	43	2092	NA	786	780
10-Heneicosene (c,t)	629-94-7	52730	-	HC	alkene	2538	0.305	57	2095	NA	861	895
Heneicosane	150-86-7	149168	-	HC	alkane	2544	0.3	57	2100	2100	912	888
Phytol	629-97-0	125892	-	Oxy	alcohol	2560	0.42	71	2116	2116	888	808
Docosane	107304-12-1	127122	-	HC	alkane	2650	0.3	57	2200	2200	892	842
Stigmastan-6,22-dien, 3,5-dedihydro-	10191-41-0	111497	-	HC	triterpene	3424	0.695	81	2945	NA	832	816
Vitamin E	64-19-7	1523058	-	Oxy	alcohol	3504	0.78	165	3022	NA	856	855

*RI is Kovats Retention Index. “Exp. RI” refers to an experimental retention index calculated for this analysis. “Library RI” refers to a tabulated RI value obtained from NIST, PubChem, or ChemSpider electronic sources.

†Similarity refers to a "forward" mass spectral match score (i.e., how well does an experimentally-obtained spectrum fit to a library entry). In the case of a forward search, the match score (out of a maximum of 1000) for the experimental spectrum is penalized for containing extra peaks that are not present in the library spectrum.

‡Reverse refers to a "backwards" mass spectral match score (i.e., how well does a library entry fit to an experimentally-obtained spectrum). A reverse search match score (out of a maximum of 1000) for the library spectrum is not penalized for having extra peaks not contained in the experimental spectrum. A reverse search only requires that peaks in the library spectrum are present in the experimental spectrum.

Health effects were classified using the International Agency for Research on Cancer (IARC) carcinogen list and the Registry of Toxic Effects of Chemical Substances (RTECS) provided by the Canadian Centre for Occupational Health and Safety (CCOHS).

G1 = Group 1 carcinogen, G2A = Group 2A carcinogen, G2B = Group 2B carcinogen, G3 = Group 3 carcinogen, M = mutagen, T = teratogen, X = toxic by other mechanisms, “–“ = no risk data available/risks mitigated by proper protective equipment.

NA = not available, HC = hydrocarbon, Het = heterocycle, Misc = miscellaneous, Nit = nitrogenated, Oxy = oxygenated

Table S4. Tentatively identified compounds from the particulate phase fraction of mainstream marijuana smoke. G1 = Group 1 carcinogen, G2A = Group 2A carcinogen, G2B = Group 2B carcinogen, G3 = Group 3 carcinogen, M = Mutagen, T = Teratogen, X = Toxic by other mechanisms, ND = No risk data available or risks mitigated by proper protective equipment.

Tentative Identification	CAS	Peak Area	Health Effects	Class	Subclass	^1t_R (s)	^2t_R (s)	Quant Mass	Exp. RI*	Lib. RI*	Reverse†	Similarity‡
Acetic acid	64-19-7	12425147	-	Oxy	carboxylic acid	224	0.35	60	650	645	978	978
3-methyl-2-butanone	563-80-4	83823	-	Oxy	ketone	224	0.45	86	650	653	909	909
2-methyl-butanal	96-17-3	82376	-	Oxy	aldehyde	274	0.59	58	681	681	946	822
Butanenitrile	109-74-0	475077	X	Nit	nitrile	282	0.965	41	686	686	955	899
(2E)-2-Butenenitrile	4786-20-3	841321	-	Nit	nitrile	296	1.05	41	695	697	963	889
2-Pentanone	107-87-9	508247	-	Oxy	ketone	296	0.78	43	694	691	954	875
Methyl thiocyanate	556-64-9	459740	X	Misc	thiocyanate	330	0.94	72	715	711	956	935
Butanenitrile, 2-methyl-	18936-17-9	1176152	X	Nit	nitrile	342	1.1	55	723	717	937	934
Butanenitrile, 3-methyl-	625-28-5	2487831	X	Nit	nitrile	352	1.14	43	729	731	909	908
Pyrazine	290-37-9	1582045	M	Het	pyrazine	358	0.625	80	732	734	968	968
1H-Pyrrole, 1-methyl-	96-54-8	737950	-	Het	pyrrole	364	0.64	80	736	735	917	895
4-methyl-2-pentanone	108-10-1	235009	-	Oxy	ketone	364	0.87	43	736	736	915	838
3-Penten-2-one, (E)-	3102-33-8	287355	X	Oxy	ketone	368	1.045	69	738	739	946	789
Pyridine	110-86-1	9542703	G2B	Het	pyridine	372	0.665	79	741	740	941	941
(Dimethylamino)-acetonitrile	926-64-7	249399	X	Nit	nitrile	380	1.035	83	746	NA	922	897
Pyrrole	109-97-7	7104916	M	Het	pyrrole	384	0.57	67	748	748	979	979
1,3,5-Cycloheptatriene	544-25-2	55934	M	HC	alicyclic HC	412	0.46	65	765	765	944	926
3,3-Dimethylacrylonitrile	4786-24-7	786277	-	Nit	nitrile	422	1.285	41	772	770	889	857
Pentanenitrile	110-59-8	343324	X	Nit	nitrile	428	1.21	54	775	777	915	858
3-Hexanone	589-38-8	87638	M	Oxy	ketone	442	0.81	57	783	783	944	782
2-Hexanone	591-78-6	127877	M	Oxy	ketone	450	0.92	58	788	789	919	825
Cyclopentanone	120-92-3	1640759	-	Oxy	ketone	454	1.055	55	791	791	980	974
1H-Pyrrole, 1-ethyl-	617-92-5	310687	-	Het	pyrrole	494	0.655	80	812	811	909	896
2-Methylpyridine	109-06-8	1797843	M	Het	pyridine	502	0.61	93	815	815	953	944

Continued on next page

Table S4. Tentatively identified compounds from the particulate phase fraction of mainstream marijuana smoke (Continued)

Tentative Identification	CAS	Peak Area	Health Effects	Class	Subclass	$^{1}t_R$ (s)	$^{2}t_R$ (s)	Quant Mass	Exp. RI*	Lib. RI*	Reverse†	Similarity‡
2-Methylpyrazine	109-08-0	4395969	M	Het	pyrazine	520	0.66	94	824	824	970	970
2-Cyclopenten-1-one	930-30-3	2122603	-	Oxy	ketone	538	1.25	82	833	832	959	952
4,4-Dimethyl-3-oxopentanenitrile	59997-51-2	1228765	X	Nit	nitrile	550	1.23	57	838	843	765	755
Pentanenitrile, 4-methyl-	542-54-1	3485465	-	Nit	nitrile	556	1.27	55	841	843	920	920
Cyclopentanone, 2-methyl-	1120-72-5	329084	-	Oxy	ketone	556	0.955	42	841	840	949	910
2-Hexanone, 4-methyl-	105-42-0	70851	-	Oxy	ketone	566	0.915	58	846	846	919	802
Cyclopentanone, 3-methyl-	1757-42-2	150877	-	Oxy	ketone	574	1.08	69	850	851	924	868
5-methyl-2-hexanone	110-12-3	396962	-	Oxy	ketone	588	0.945	43	856	857	943	899
2-Furanmethanol	98-00-0	973987	G2B	Het	furan	590	0.65	98	857	858	954	939
Ethylbenzene	100-41-4	2234959	G2B	HC	monocyclic aromatic HC	600	0.46	91	862	860	952	952
Pyridine, 3-methyl-	108-99-6	1048871	M	Het	pyridine	602	0.79	93	863	863	952	941
2-Propanone, 1-(acetyloxy)-	592-20-1	12941977	-	Oxy	ketone	608	1.485	43	866	867	970	948
m-Xylene	108-38-3	2852196	G3	HC	monocyclic aromatic HC	618	0.465	91	870	870	957	957
o-Xylene	95-47-6	52148	G3	HC	monocyclic aromatic HC	630	0.465	91	876	876	925	855
Phenylethyne	536-74-3	308920	X	HC	monocyclic aromatic HC	636	0.51	102	879	877	927	896
Hexanenitrile	628-73-9	473963	X	Nit	nitrile	636	1.195	54	879	879	928	905
Pyridine, 2,6-dimethyl-	108-48-5	207125	M	Het	pyridine	642	0.505	107	881	880	914	851
1,5-Heptadiene, 2,6-dimethyl-	6709-39-3	140490	-	HC	alkene	644	0.345	69	882	882	888	834
2-heptanone	110-43-0	604107	M	Oxy	ketone	660	0.88	58	890	890	930	928
1-nonene	124-11-8	58573	-	HC	alkene	662	0.345	56	891	890	913	837

Continued on next page

Table S4. Tentatively identified compounds from the particulate phase fraction of mainstream marijuana smoke (Continued)

Tentative Identification	CAS	Peak Area	Health Effects	Class	Subclass	^1t_R (s)	^2t_R (s)	Quant Mass	Exp. RI*	Lib. RI*	Reverse†	Similarity‡
Styrene	100-42-5	3579752	G2A	HC	monocyclic aromatic HC	666	0.5	104	893	894	952	952
p-Xylene	106-42-3	1052166	G3	HC	monocyclic aromatic HC	668	0.485	91	894	894	944	942
Cyclohexanone	108-94-1	99791	G3	Oxy	ketone	674	1.045	55	897	896	922	811
Annulene	629-20-9	139454	-	HC	monocyclic aromatic HC	682	0.51	104	900	894	900	802
Nonane	111-84-2	57625	X	HC	alkane	682	0.33	57	900	900	939	864
3-Methylcyclopentyl acetate	24070-70-0	162858	-	Oxy	ester	684	0.56	43	901	904	867	796
Pyridine, 2-ethyl-	100-71-0	407090	-	Het	pyridine	690	0.545	106	904	904	910	886
2-Cyclopenten-1-one, 2-methyl-	1120-73-6	2089619	-	Oxy	ketone	694	1.015	67	906	905	961	959
Furfuryl formate	13493-97-5	122285	-	Oxy	ester	698	0.755	81	908	904	952	848
2-Acetylfuran	1192-62-7	2597557	M	Het	furan	708	1.035	95	912	911	941	940
Pyrazine, 2,6-dimethyl-	108-50-9	1646230	M	Het	pyrazine	710	0.595	108	913	913	945	932
Pyrazine, 2,5-dimethyl-	123-32-0	279855	M	Het	pyrazine	716	0.59	42	916	915	912	849
Pyrazine, ethyl-	13925-00-3	673428	M	Het	pyrazine	718	0.6	107	917	917	961	948
Anisole	100-66-3	47452	-	Oxy	ether	722	0.57	65	919	918	929	846
2,3-dimethylpyrazine	5910-89-4	925690	X	Het	pyrazine	722	0.61	67	919	919	929	905
Cumene	98-82-8	142922	G2B	HC	monocyclic aromatic HC	736	0.455	105	925	925	952	914
alpha-Thujene	02/05/2867	156090	-	HC	monoterpene	744	0.35	93	929	929	913	891
2,4-dimethylpyridine	108-47-4	259182	M	Het	pyridine	754	0.625	106	933	932	934	861
Pyrazine, ethenyl-	4177-16-6	215011	-	Het	pyrazine	760	0.61	106	936	935	845	800
alpha-Pinene	80-56-8	322423	-	HC	monoterpene	760	0.365	55	936	927	916	914
2-methylcyclohexanone	583-60-8	41977	X	Oxy	ketone	772	0.89	84	942	937	802	714

Continued on next page

Table S4. Tentatively identified compounds from the particulate phase fraction of mainstream marijuana smoke (Continued)

Tentative Identification	CAS	Peak Area	Health Effects	Class	Subclass	^1t_R (s)	^2t_R (s)	Quant Mass	Exp. RI*	Lib. RI*	Reverse†	Similarity‡
Pyridine, 2,3-dimethyl-	583-61-9	176593	M	Het	pyridine	780	0.63	106	945	945	927	873
Benzene, 2-propenyl-	300-57-2	275674	X	HC	monocyclic aromatic HC	786	0.45	117	948	947	936	926
Camphene	79-92-5	225909	-	HC	monoterpene	798	0.38	93	953	954	950	930
Benzene, propyl-	103-65-1	1587107	-	HC	monocyclic aromatic HC	804	0.44	91	956	956	978	946
Cyclopentanone, 3,4-bis(methylene)-	27646-73-7	146127	-	Oxy	ketone	812	0.88	79	959	949	850	764
3-Ethyltoluene	620-14-4	1359132	-	HC	monocyclic aromatic HC	820	0.445	105	964	963	944	943
3,3-dimethylacrylic acid	541-47-9	115096	-	Oxy	carboxylic acid	822	0.545	83	964	953	831	745
2-Ethyltoluene	611-14-3	1364376	-	HC	monocyclic aromatic HC	826	0.425	105	966	967	938	938
3-methyl-2-cyclopenten-1-one	2758-18-1	829456	-	Oxy	ketone	828	1.38	96	968	969	941	925
Benzaldehyde	100-52-7	142955	M	Oxy	aldehyde	830	0.84	106	968	970	905	798
1,2,4-trimethylbenzene	95-63-6	573435	M	HC	monocyclic aromatic HC	838	0.435	105	972	976	940	939
Dimethyl trisulfide	3658-80-8	101042	-	Misc	trisulfide	840	0.485	126	973	973	916	878
(R)-Sabinene	3387-41-5	336230	-	HC	monoterpene	848	0.385	93	976	976	929	911
Methyl 2-furoate	611-13-2	315709	-	Oxy	ester	852	0.855	95	978	978	942	769
3-methyl-2(5H)-furanone	22122-36-7	287215	-	Oxy	ketone	854	1.755	98	980	979	924	861
4-Ethyltoluene	622-96-8	161892	-	HC	monocyclic aromatic HC	858	0.45	120	981	977	928	912
beta-pinene	18172-67-3	2555045	-	HC	monoterpene	858	0.37	93	981	980	948	936

Continued on next page

Table S4. Tentatively identified compounds from the particulate phase fraction of mainstream marijuana smoke (Continued)

Tentative Identification	CAS	Peak Area	Health Effects	Class	Subclass	$^{1}t_{R}$ (s)	$^{2}t_{R}$ (s)	Quant Mass	Exp. RI*	Lib. RI*	Reverse†	Similarity‡
Heptanenitrile	629-08-3	127966	-	Nit	nitrile	858	1.065	82	981	985	890	777
beta-terpinene	99-84-3	326085	-	HC	monoterpene	860	0.26	93	982	988	889	850
Phenol	108-95-2	23963163	G3	Oxy	alcohol	862	0.5	94	983	984	968	964
alpha-Methylstyrene	98-83-9	228795	G2B	HC	monocyclic aromatic HC	866	0.46	118	985	986	942	914
5-Hepten-2-one, 6-methyl-	110-93-0	1141547	-	Oxy	ketone	868	0.77	43	986	985	949	935
Benzonitrile	100-47-0	6747487	M	Nit	nitrile	874	1.01	103	989	989	941	941
beta-Myrcene	123-35-3	5721453	G2B	HC	monoterpene	878	0.355	69	990	986	957	950
2-Octanone	111-13-7	176549	-	Oxy	ketone	880	0.795	58	991	990	898	815
Cyclohexanone, 4-methylidene-	29648-66-6	160763	-	Oxy	ketone	880	1.1	82	991	NA	813	754
1-Decene	872-05-9	452605	-	HC	alkene	882	0.325	56	992	992	913	908
trans,trans-2,8-decadiene	19398-85-7	164847	-	HC	alkene	882	0.44	67	992	998	753	692
3-Methylstyrene	100-80-1	115001	G3	HC	monocyclic aromatic HC	884	0.445	117	993	991	856	798
2-Furanmethanol, acetate	623-17-6	323533	M	Oxy	ester	884	0.68	81	993	995	950	936
3,3-dimethyl-6-methylenecyclohexene	20185-16-4	393782	-	HC	alicyclic HC	884	0.43	107	993	997	856	855
1H-Pyrrole, 1-butyl-	589-33-3	166688	-	Het	pyrrole	888	0.585	80	995	NA	913	897
1,2-Propadienylbenzene	2327-99-3	53815	-	HC	monocyclic aromatic HC	888	0.475	116	995	NA	895	801
2-Cyclopenten-1-one, 3,4-dimethyl-	30434-64-1	196277	-	Oxy	ketone	888	1.21	95	995	986	901	837
4-methylstyrene	NA	806934	-	HC	monocyclic aromatic HC	894	0.49	118	997	995	911	871
Pyrazine, 2-ethyl-6-methyl-	13925-03-6	495075	-	Het	pyrazine	896	0.54	121	998	998	923	918
Benzofuran	2792-39-4	443879	G2B	Het	furan	898	0.555	90	999	1000	957	940

Continued on next page

Table S4. Tentatively identified compounds from the particulate phase fraction of mainstream marijuana smoke (Continued)

Tentative Identification	CAS	Peak Area	Health Effects	Class	Subclass	$^{1}t_R$ (s)	$^{2}t_R$ (s)	Quant Mass	Exp. RI*	Lib. RI*	Reverse†	Similarity‡
2,6-Octadiene, 2,6-dimethyl-	2792-39-4	1081849	-	HC	alkene	898	0.31	69	999	990	919	919
Decane	124-18-5	347655	X	HC	alkane	902	0.305	57	1001	1000	942	932
Pyrazine, trimethyl-	14667-55-1	1826615	-	Het	pyrazine	906	0.495	42	1003	1003	914	914
Pyrazine, 2-ethyl-3-methyl-	15707-23-0	237534	-	Het	pyrazine	906	0.505	121	1003	1003	852	841
Dehydroxylinalool oxide	54750-69-5	135697	-	Het	furan	914	0.45	67	1007	1007	889	863
alpha-Phellandrene	99-83-2	1494367	-	HC	monoterpene	918	0.36	93	1009	1009	923	919
Isobutylbenzene	538-93-2	32384	-	HC	monocyclic aromatic HC	920	0.425	134	1010	1009	966	917
1-Pyrrolidinylacetonitrile	29134-29-0	96879	X	Het	pyridine	922	0.925	82	1011	NA	902	839
(-)-3-carene	20296-50-8	1115470	-	HC	monoterpene	924	0.35	93	1012	1010	932	929
Oxalic acid, allyl octyl ester	NA	200293	-	Oxy	ester	930	0.33	57	1015	NA	872	850
3-Pyridinecarbonitrile	100-54-9	477481	X	Het	pyridine	930	1.115	104	1015	1012	940	839
7-vinylbicyclo[4.2.0]oct-1-ene	NA	144355	-	HC	alicyclic HC	938	0.38	91	1019	1023	779	760
gamma-Terpinene	99-85-4	1085572	M	HC	monoterpene	940	0.36	93	1020	1023	913	910
2,6-dimethylnonane	17302-28-2	53493	-	HC	alkane	946	0.32	71	1023	1024	897	790
Benzoxazole	273-53-0	27508	-	Nit	oxazole	946	0.615	64	1023	NA	901	790
Benzene, (2-methyl-2-propenyl)-	3290-53-7	135613	-	HC	monocyclic aromatic HC	948	0.415	117	1024	1022	894	824
p-Methylanisole	104-93-8	185341	X	Oxy	ether	948	0.52	122	1024	1024	938	916
Benzene, 1,2,3-trimethyl-	526-73-8	881976	M	HC	monocyclic aromatic HC	950	0.47	105	1025	1028	939	929
Mesitylene	108-67-8	881976	M	HC	monocyclic aromatic HC	950	0.47	105	1025	1013	941	933
4,4-Dimethyl-2-cyclopenten-1-one	22748-16-9	82207	-	Oxy	ketone	950	1.35	95	1025	NA	904	793

Continued on next page

Table S4. Tentatively identified compounds from the particulate phase fraction of mainstream marijuana smoke (Continued)

Tentative Identification	CAS	Peak Area	Health Effects	Class	Subclass	$^{1}t_R$ (s)	$^{2}t_R$ (s)	Quant Mass	Exp. RI*	Lib. RI*	Reverse†	Similarity‡
4-butylcyclohexene	21524-26-5	131289	-	HC	alicyclic HC	954	0.365	54	1026	1036	769	753
Bicyclo[4.2.0]oct-1-ene, 7-endo-ethenyl-	NA	40281	-	HC	alicyclic HC	956	0.385	106	1027	NA	830	816
o-Cymene	527-84-4	3399473	-	HC	monocyclic aromatic HC	956	0.4	119	1027	1027	963	952
(+)-Carvomenthene	1195-31-9	393503	-	HC	monoterpene	956	0.345	95	1027	1028	897	893
2-hydroxy-3-methyl-2-cyclopenten-1-one	80-71-7	809831	M	Oxy	ketone	962	0.835	112	1031	1028	929	918
Indane	496-11-7	1503230	-	HC	alicyclic HC	962	0.49	117	1030	1030	921	920
D-Limonene	5989-27-5	8749710	G3	HC	monoterpene	966	0.35	68	1032	1031	945	944
beta-phellandrene	555-10-2	3257485	-	HC	monoterpene	970	0.385	93	1034	1033	930	930
Eucalyptol	470-82-6	1353566	T	Oxy	alcohol	974	0.415	43	1036	1035	934	925
2-Acetylpyridine	1122-62-9	200388	M	Het	pyridine	976	0.73	79	1037	1036	933	880
alpha-ocimene	08/10/6874	507472	-	HC	monoterpene	976	0.325	93	1037	1037	929	921
Isocarvestrene	1461-27-4	25815	-	HC	monoterpene	986	0.34	93	1042	1032	805	731
Benzene, 3-butenyl-	768-56-9	925404	-	HC	monocyclic aromatic HC	994	0.425	91	1046	1038	954	948
trans-beta-Ocimene	13877-91-3	2665767	-	HC	monoterpene	998	0.345	93	1048	1046	948	947
Indene	95-13-6	2444987	-	HC	polycyclic aromatic HC	1000	0.515	116	1049	1049	935	935
1,2-Diethylbenzene	135-01-3	74047	M	HC	monocyclic aromatic HC	1002	0.41	119	1050	1048	835	791
Benzene, 1-methyl-3-propyl-	1074-43-7	402885	-	HC	monocyclic aromatic HC	1010	0.415	105	1054	1055	908	895

Continued on next page

Table S4. Tentatively identified compounds from the particulate phase fraction of mainstream marijuana smoke (Continued)

Tentative Identification	CAS	Peak Area	Health Effects	Class	Subclass	$^{1}t_R$ (s)	$^{2}t_R$ (s)	Quant Mass	Exp. RI*	Lib. RI*	Reverse†	Similarity‡
1-(2-methylbutly)-Pyrrole	13678-55-2	510794	-	Het	pyrrole	1012	0.555	81	1055	NA	905	783
Benzene, 1-methyl-2-(2-propenyl)-	1587-04-8	71954	-	HC	monocyclic aromatic HC	1016	0.435	117	1057	1055	881	807
o-Cresol	95-48-7	3029911	G3	Oxy	alcohol	1018	0.49	108	1058	1058	944	943
Benzene, 1-methyl-4-propyl	1074-55-1	311167	-	HC	monocyclic aromatic HC	1020	0.405	105	1058	1059	889	872
3,5-dimethyl-1,2-cyclopentadione (caramel dione)	13494-07-0	70043	-	Oxy	ketone	1020	0.76	126	1059	1048	835	793
Benzene, 1,4-diethyl	105-05-5	323348	-	HC	monocyclic aromatic HC	1022	0.405	119	1059	1057	908	901
Butylbenzene	104-51-8	508729	-	HC	monocyclic aromatic HC	1022	0.425	92	1059	1058	910	895
Phenyl acetate	122-79-2	459552	-	Oxy	ester	1024	0.83	94	1061	1065	944	917
alpha-terpinene	99-86-5	144721	M	HC	monoterpene	1026	0.33	107	1061	1058	904	879
2-Cyclopenten-1-one, 2,3,4-trimethyl-	28790-86-5	94737	-	Oxy	ketone	1026	0.935	109	1062	1058	902	817
Benzene, 1-propynyl-	673-32-5	125307	-	HC	monocyclic aromatic HC	1034	0.46	115	1065	1058	909	811
2-Amino-4-methylpyrimidine	108-52-1	260315	-	Het	pyrimidine	1036	0.575	109	1066	NA	846	766
Benzene, 1-methyl-2-propyl	1074-17-5	335361	-	HC	monocyclic aromatic HC	1040	0.435	105	1068	1065	898	885
Benzene, isobutenyl-	768-49-0	122986	-	HC	monocyclic aromatic HC	1044	0.4	117	1070	1071	885	855
2-Acetylpyrrole	1072-83-9	736197	M	Het	pyrrole	1046	0.78	94	1071	1072	933	880

Continued on next page

Table S4. Tentatively identified compounds from the particulate phase fraction of mainstream marijuana smoke (Continued)

Tentative Identification	CAS	Peak Area	Health Effects	Class	Subclass	^1t_R (s)	^2t_R (s)	Quant Mass	Exp. RI*	Lib. RI*	Reverse†	Similarity‡
Acetophenone	98-86-2	720372	M	Oxy	ketone	1046	0.845	105	1071	1071	958	952
2-Pyridinecarbonitrile	100-70-9	777742	-	Het	pyridine	1046	1.36	104	1072	1069	968	901
4-thujanol	15826-82-1	509930	-	Oxy	alcohol	1050	0.51	71	1073	1073	940	916
Benzenamine, 2-methyl-	636-21-5	51625	M	Nit	amine	1054	0.62	106	1075	1072	920	787
5-Undecene, 4-methyl-	NA	171241	-	HC	alkene	1054	0.325	69	1075	NA	836	804
2-Pyrrolidinone	616-45-5	2747459	M	Het	pyrrole	1060	1.465	85	1078	1078	949	913
Pyrazine, 3-ethyl-2,5-dimethyl-	13360-65-1	116804	-	Het	pyrazine	1060	0.465	136	1078	1078	916	807
Benzene, 2-ethyl-1,4-dimethyl-	1758-88-9	151788	-	HC	monocyclic aromatic HC	1060	0.415	119	1078	1077	860	841
m-Cresol	108-39-4	10111666	G3	Oxy	alcohol	1062	0.495	108	1079	1079	948	941
3-ethyl-2-cyclopenten-1-one	5682-69-9	201452	-	Oxy	ketone	1062	1.235	81	1079	1071	894	827
Benzene, 4-ethyl-1,2-dimethyl-	934-80-5	396644	-	HC	monocyclic aromatic HC	1066	0.41	119	1081	1079	902	896
Octanenitrile	124-12-9	111024	-	Nit	nitrile	1068	0.97	82	1082	1082	891	815
3-Methylbenzaldehyde	620-23-5	71266	-	Oxy	aldehyde	1070	0.55	120	1083	1081	852	710
1,10-Undecadiene	13688-67-0	115658	-	HC	alkene	1072	0.33	67	1084	1083	936	855
p-cymenene	1195-32-0	382162	-	HC	monoterpene	1074	0.43	117	1085	1085	934	917
Z-1,8-Dodecadiene	NA	413966	-	HC	alkene	1078	0.315	67	1087	NA	882	795
Benzene, 3-ethyl-1,2-dimethyl	933-98-2	276928	-	HC	monocyclic aromatic HC	1078	0.4	119	1087	1088	904	892
Linalyl oxide	5989-33-3	189676	-	Het	furan	1082	0.55	59	1089	1088	901	875
Terpinolene (delta-terpinene)	586-62-9	2818988	-	HC	monoterpene	1082	0.335	43	1089	1089	924	922
1H-Indene, 2,3-dihydromethyl-	27133-93-3	56842	-	HC	polycyclic aromatic HC	1082	0.44	132	1089	1085	897	866
Guaiacol	90-05-1	2679865	X	Oxy	alcohol	1084	0.675	109	1090	1090	973	956

Continued on next page

Table S4. Tentatively identified compounds from the particulate phase fraction of mainstream marijuana smoke (Continued)

Tentative Identification	CAS	Peak Area	Health Effects	Class	Subclass	^1t_R (s)	^2t_R (s)	Quant Mass	Exp. RI*	Lib. RI*	Reverse†	Similarity‡
2-Nonanone	821-55-6	128119	-	Oxy	ketone	1086	0.72	58	1091	1091	925	767
1-Undecene	821-95-4	801561	-	HC	alkene	1088	0.295	56	1091	1091	937	935
Benzene, 2-ethyl-1,3-dimethyl-	04/04/2870	74533	-	HC	monocyclic aromatic HC	1088	0.425	119	1092	1087	859	806
alpha-Naginatene	15186-51-3	39058	-	Het	furan	1090	0.39	150	1092	1093	841	797
3-Methylene-1,5,5-trimethylcyclohexene	16609-28-2	57007	-	HC	alicyclic HC	1090	0.33	121	1092	NA	883	825
m-cymenene	1124-20-5	3205300	-	HC	monocyclic aromatic HC	1090	0.43	91	1092	1092	950	919
(+)-Fenchone	1195-79-5	995577	-	Oxy	ketone	1090	0.76	81	1093	1088	937	935
3-Methylbenzonitrile	620-22-4	930665	-	Nit	nitrile	1094	0.985	117	1095	1094	945	936
Benzene, 1-ethenyl-4-ethyl-	07/07/3454	176971	-	HC	monocyclic aromatic HC	1098	0.45	117	1096	NA	894	864
Benzoic acid, methyl ester	93-58-3	291583	-	Oxy	ester	1102	0.66	105	1098	1098	929	880
4,4-dimethylcyclohexenone	1073-13-8	45153	-	Oxy	ketone	1104	0.955	124	1100	1101	808	692
Linalool	78-70-6	7227942	M	Oxy	alcohol	1106	0.43	71	1100	1101	953	953
Undecane	1120-21-4	906616	X	HC	alkane	1106	0.29	57	1100	1100	947	935
sec-Pentylbenzene	2719-52-0	143311	-	HC	monocyclic aromatic HC	1110	0.39	105	1102	1098	844	802
2,5-dimethylstyrene	2039-89-6	204202	-	HC	monocyclic aromatic HC	1112	0.44	117	1103	1101	874	835
2-Undecene, (E)-	693-61-8	62499	-	HC	alkene	1114	0.29	70	1104	1104	870	858
beta-terpineol	7299-41-4	622740	-	Oxy	alcohol	1114	0.55	43	1105	1101	917	903
Benzofuran, 2-methyl-	4265-25-2	606545	-	Het	furan	1122	0.54	131	1109	1108	919	903
6,7-dihydro-5H-Cyclopentapyrazine	NA	320737	-	Het	pyrazine	1126	0.6	119	1111	1104	955	869

Continued on next page

Table S4. Tentatively identified compounds from the particulate phase fraction of mainstream marijuana smoke (Continued)

Tentative Identification	CAS	Peak Area	Health Effects	Class	Subclass	$^{1}t_{R}$ (s)	$^{2}t_{R}$ (s)	Quant Mass	Exp. RI*	Lib. RI*	Reverse†	Similarity‡
Phenol, 2,6-dimethyl-	576-26-1	460482	X	Oxy	alcohol	1126	0.51	107	1111	1111	949	933
2,3-Cyclopentenopyridine	533-37-9	584415	-	Het	pyridine	1136	0.58	118	1116	NA	896	892
1,3,8-Menthatriene	18368-95-1	540607	-	HC	monoterpene	1136	0.38	91	1116	1118	913	895
Benzene, 1-ethyl-4-methoxy-	1515-95-3	66875	-	Oxy	ether	1136	0.48	121	1116	1110	919	893
Phenylethyl Alcohol	60-12-8	135274	X	Oxy	alcohol	1142	0.59	92	1120	1119	905	814
Benzoxazole, 2-methyl-	95-21-6	90966	-	Nit	oxazole	1144	0.57	133	1121	NA	913	825
2-Cyclopenten-1-one, 3-ethyl-2-hydroxy-	21835-01-8	159603	-	Oxy	ketone	1144	0.775	83	1121	1121	934	876
Fenchol, exo-	22627-95-8	4705295	-	Oxy	alcohol	1150	0.48	81	1124	1120	958	954
Benzene, 1,2,3,5-tetramethyl	527-53-7	151631	X	HC	monocyclic aromatic HC	1150	0.415	119	1124	1123	785	772
o-Cymenene	7399-49-7	129571	-	HC	monoterpene	1152	0.455	117	1125	1117	890	842
(E)-cyclodecene	2198-20-1	127075	-	HC	alicyclic HC	1154	0.34	54	1126	1122	846	798
Benzene, 1-butenyl-, (E)-	1005-64-7	587104	-	HC	monocyclic aromatic HC	1160	0.45	117	1129	1120	911	900
Alloocimene	3016-19-1	287176	-	HC	monoterpene	1160	0.37	121	1129	1128	926	916
2-Cyclohexen-1-ol, 1-methyl-4-(1-methylethyl)-, trans-	29803-81-4	240659	-	Oxy	alcohol	1160	0.49	43	1129	1130	828	814
Benzyl methyl ketone	103-79-7	567846	-	Oxy	ketone	1162	0.875	43	1130	1124	947	916
2,6,6-Trimethylbicyclo[3.1.1]hepta-2-ol	98510-89-5	1625956	-	Oxy	alcohol	1164	0.5	99	1131	1130	909	909
3-Pyridinol, 2-methyl-	1121-25-1	363820	-	Het	pyridine	1168	0.53	109	1133	NA	889	802
Cosmene	460-01-5	59488	-	HC	monoterpene	1168	0.43	119	1133	1132	919	880
2,5-Pyrrolidinedione, 1-ethyl-	2314-78-5	87341	-	Het	pyrrole	1170	1.345	56	1135	NA	911	753

Continued on next page

Table S4. Tentatively identified compounds from the particulate phase fraction of mainstream marijuana smoke (Continued)

Tentative Identification	CAS	Peak Area	Health Effects	Class	Subclass	$^{1}t_R$ (s)	$^{2}t_R$ (s)	Quant Mass	Exp. RI*	Lib. RI*	Reverse†	Similarity‡
1-Methyl-2-phenylcyclopropane 1	NA	173563	-	HC	alicyclic HC	1172	0.465	117	1135	NA	873	854
3,4-Dimethylcumene	NA	353153	-	HC	monocyclic aromatic HC	1174	0.385	133	1136	1139	889	887
p-Mentha-1,5,8-triene	21195-59-5	629040	-	HC	monoterpene	1180	0.4	91	1140	1139	923	922
2-Ethylphenol	90-00-6	1173803	X	Oxy	alcohol	1182	0.45	107	1141	1140	939	920
Benzyl nitrile	140-29-4	3474037	M	Nit	nitrile	1184	0.985	90	1142	1142	938	935
Cyclopentane, hexyl-	4457-00-5	46920	-	HC	alicyclic HC	1184	0.32	69	1142	1141	883	802
4-Methylindane	824-22-6	406876	-	HC	polyclyclic aromatic HC	1188	0.41	117	1144	1142	887	878
N,N,O-Triacetylhydroxylamine	17720-63-7	441976	-	Misc	acetamide	1188	1.605	43	1145	NA	931	812
Ipsdienol	35628-00-3	392218	-	Oxy	alcohol	1190	0.495	85	1145	1147	830	819
3-Methylheptyl acetate	72218-58-7	104481	-	Oxy	ester	1192	0.565	70	1146	NA	953	869
Epoxyterpinolene	NA	1934593	-	Oxy	epoxide	1194	0.575	79	1147	1138	966	769
o-Ethylbenzonitrile	34136-59-9	69989	-	Nit	nitrile	1198	0.845	116	1149	NA	906	773
Cis-2-pinanol	4948-28-1	223166	-	Oxy	alcohol	1204	0.535	99	1152	1143	889	888
5-Methylindane	874-35-1	180401	-	HC	alicyclic HC	1206	0.435	117	1153	1147	829	816
1,4-Dimethyl-4-acetylcyclohexene	43219-68-7	32841	-	Oxy	ketone	1208	0.695	109	1155	1152	877	813
Phenol, 2,4-dimethyl-	105-67-9	81632	-	Oxy	alcohol	1210	0.455	93	1156	1156	908	858
2-Undecyne	60212-29-5	47407	-	HC	alkyne	1212	0.33	68	1157	1152	824	757
1-Methylindene	767-59-9	436825	-	HC	polyclyclic aromatic HC	1212	0.475	115	1157	1149	883	836
2,6-Dimethylbenzonitrile	6575-13-9	61839	-	Nit	nitrile	1212	0.815	116	1157	NA	910	821

Continued on next page

Table S4. Tentatively identified compounds from the particulate phase fraction of mainstream marijuana smoke (Continued)

Tentative Identification	CAS	Peak Area	Health Effects	Class	Subclass	$^{1}t_R$ (s)	$^{2}t_R$ (s)	Quant Mass	Exp. RI*	Lib. RI*	Reverse†	Similarity‡
2,5-Pyrrolidinedione	123-56-8	374850	-	Het	pyrrole	1214	1.235	99	1158	NA	892	760
1H-Pyrrole-3-carbonitrile	7126-38-7	1070634	-	Het	pyrrole	1216	0.795	92	1159	NA	877	821
Glutarimide	1121-89-7	4097543	-	Nit	imide	1216	1.27	42	1159	1154	884	882
Phenol, 3,4-dimethyl-	95-65-8	175301	-	Oxy	alcohol	1216	0.475	107	1159	1155	875	787
4-Methoxystyrene	637-69-4	70954	-	Oxy	ether	1216	0.53	134	1159	1158	847	756
Naphthalene, 1,4-dihydro-	612-17-9	840051	-	HC	alicyclic HC	1218	0.505	130	1160	1167	910	903
Benzene, pentyl-	538-68-1	558678	-	HC	monocyclic aromatic HC	1218	0.4	92	1160	1160	941	932
5-Pentylcyclohexa-1,3-diene	56318-84-4	163521	-	HC	alicyclic HC	1222	0.35	79	1162	1161	860	821
2-Undecene, 9-methyl-, (Z)-	74630-45-8	41504	-	HC	alkene	1224	0.31	70	1163	NA	856	791
p-Tolyl acetate	140-39-6	155228	-	Oxy	ester	1232	0.79	108	1167	1166	843	773
Acetophenone, 2'-hydroxy-	118-93-4	125053	-	Oxy	ketone	1232	0.765	121	1167	1167	858	777
Naphthalene, 1,2,3,4-tetrahydro-	119-64-2	129659	X	HC	polyclyclic aromatic HC	1232	0.435	104	1167	1164	811	717
p-Isobutyltoluene	06/04/5161	275852	-	HC	monocyclic aromatic HC	1234	0.42	105	1168	NA	854	843
1H-Pyrrole-2,5-dione, 3,4-dimethyl-	17825-86-4	944293	-	Het	pyrrole	1236	1.035	125	1170	NA	961	835
Undecane, 3-methyl-	1002-43-3	52333	-	HC	alkane	1238	0.305	57	1170	1170	911	782
4-Ethylphenol	123-07-9	3759309	X	Oxy	alcohol	1238	0.48	107	1171	1170	929	927
1-Propanone, 1-phenyl-	93-55-0	211300	X	Oxy	ketone	1238	0.72	105	1171	1173	942	908
Pyrrolidine, 1-acetyl-	4030-18-6	160547	-	Het	pyrrole	1240	1.395	70	1172	1162	934	861
Naphthalene, 1,2-dihydro-	447-53-0	301186	-	HC	alicyclic HC	1240	0.46	130	1172	1166	946	937
(+)-Borneol	464-43-7	3907231	-	Oxy	alcohol	1252	0.51	95	1178	1177	926	926

Continued on next page

Table S4. Tentatively identified compounds from the particulate phase fraction of mainstream marijuana smoke (Continued)

Tentative Identification	CAS	Peak Area	Health Effects	Class	Subclass	$^{1}t_R$ (s)	$^{2}t_R$ (s)	Quant Mass	Exp. RI*	Lib. RI*	Reverse†	Similarity‡
Benzonitrile, 3,5-dimethyl-	22445-42-7	177588	-	Nit	nitrile	1256	0.865	116	1180	NA	895	881
1H-Pyrrole, 1-(2-furanylmethyl)-	1438-94-4	1683279	-	Het	pyrrole	1258	0.56	81	1181	1182	936	922
Benzene, (1-methyl-1-butenyl)-	53172-84-2	113835	-	HC	monocyclic aromatic HC	1258	0.4	131	1181	1184	870	847
Phenol, 2,3-dimethyl-	526-75-0	363156	-	Oxy	alcohol	1258	0.485	107	1181	1180	909	873
1H-Pyrrolo[2,3-B]pyridine	271-63-6	100746	-	Het	pyridine	1262	1.425	118	1184	1192	864	744
1,11-Dodecadiene	5876-87-9	313414	-	HC	alkene	1262	0.315	55	1183	1179	926	888
Limonen-4-ol	01/02/3419	1980399	-	Oxy	alcohol	1262	0.45	69	1183	1181	903	855
4-Terpineol	562-74-3	1590323	-	Oxy	alcohol	1266	0.46	71	1185	1186	910	908
(3Z,5E)-1,3,5-Undecatriene	19883-27-3	48622	-	HC	alkene	1268	0.38	80	1186	1187	923	835
2-Isopropylphenol	88-69-7	215658	-	Oxy	alcohol	1270	0.475	121	1188	1184	888	834
5-Dodecene, (E)-	7206-16-8	35095	-	HC	alkene	1272	0.305	55	1189	1182	884	811
1H-Indene, 1,3-dimethyl-	2177-48-2	487632	-	HC	alicyclic HC	1274	0.44	129	1190	NA	907	889
p-Cymen-8-ol	1197-01-9	3200547	-	Oxy	alcohol	1276	0.49	135	1191	1190	947	943
1H-Indene, 2,3-dihydro-1,6-dimethyl-	17059-48-2	427087	-	HC	alicyclic HC	1278	0.39	131	1192	NA	909	907
1-Dodecene	112-41-4	763252	-	HC	alkene	1278	0.3	56	1192	1192	916	916
Ethanone, 1-(3-methylphenyl)-	585-74-0	374857	-	Oxy	ketone	1278	0.795	119	1192	1192	956	920
Naphthalene	91-20-3	3472044	G2B	HC	polycyclic aromatic HC	1280	0.55	128	1193	1192	961	958
Phenol, 2-methoxy-4-methyl-	93-51-6	462417	M	Oxy	alcohol	1282	0.64	123	1194	1194	936	930
4-Dodecene	2030-84-4	29606	-	HC	alkene	1284	0.295	56	1195	1187	881	830
alpha-Terpineol	98-55-5	9365585	-	Oxy	alcohol	1292	0.475	59	1199	1198	953	943
1H-Indene, 2,3-dihydro-1,3-dimethyl-	4175-53-5	83583	-	HC	alicyclic HC	1294	0.42	131	1200	NA	825	784

Continued on next page

Table S4. Tentatively identified compounds from the particulate phase fraction of mainstream marijuana smoke (Continued)

Tentative Identification	CAS	Peak Area	Health Effects	Class	Subclass	^1t_R (s)	^2t_R (s)	Quant Mass	Exp. RI*	Lib. RI*	Reverse†	Similarity‡
Dodecane	112-40-3	1171663	-	HC	alkane	1294	0.295	57	1200	1200	942	940
Phenol, 3-ethyl-	620-17-7	26143	-	Oxy	alcohol	1294	0.515	133	1200	1195	859	791
Ethyl-2-benzofuran	3131-63-3	249985	-	Het	furan	1300	0.5	131	1204	1207	875	787
m-Ethylbenzonitrile	34136-57-7	100480	-	Nit	nitrile	1304	0.935	116	1206	Na	912	766
Phenol, 2,4,6-trimethyl-	527-60-6	185633	M	Oxy	alcohol	1310	0.475	121	1210	1211	944	895
alpha-Phellandrene epoxide	72138-69-3	57317	-	Oxy	epoxide	1310	0.5	92	1210	1211	817	758
Undecane, 2,6-dimethyl-	17301-23-4	1051696	-	HC	alkane	1316	0.305	57	1213	1213	953	942
3-Carvomenthenol	16721-39-4	358871	-	Oxy	alcohol	1318	0.49	84	1214	1214	865	810
Benzofuran, 4,7-dimethyl-	28715-26-6	529888	-	Het	furan	1332	0.5	145	1222	1220	876	868
2,3-Dihydrobenzofuran	496-16-2	9360544	-	Het	furan	1336	0.48	120	1224	1224	911	897
2,3-Dimethylbenzofuran	3782-00-1	103816	-	Het	furan	1340	0.515	131	1227	NA	851	752
trans-Chrysanthenyl acetate	54324-99-1	745903	-	Oxy	ester	1342	0.445	43	1228	NA	731	725
Furan, 3-phenyl-	13679-41-9	139070	-	Het	furan	1344	0.53	115	1229	1228	923	848
Phenol, 2-ethyl-6-methyl-	1687-64-5	599337	-	Oxy	alcohol	1346	0.44	121	1230	1236	910	834
1H-Pyrrole-2,5-dione, 3-ethyl-4-methyl-	20189-42-8	259561	-	Het	pyrrole	1358	0.95	139	1237	1238	914	891
Phenol, 4-ethyl-3-methyl-	1123-94-0	1026040	-	Oxy	alcohol	1364	0.45	121	1241	1239	920	891
Benzenepropanenitrile	645-59-0	3554324	-	Nit	nitrile	1368	1.01	91	1243	1243	950	941
Cyclohexane, hexyl-	4292-75-5	26323	-	HC	alicyclic HC	1370	0.32	82	1244	1244	904	780
1H-Indene, 2,3-dimethyl-	4773-82-4	66291	-	HC	polyclyclic aromatic HC	1370	0.445	129	1244	NA	864	780
2-Butanone, 4-phenyl-	2550-26-7	78349	-	Oxy	ketone	1376	0.79	105	1248	1251	898	849
Isoquinoline	119-65-3	245664	M	Nit	aza-arene	1378	0.64	129	1249	1255	945	918
2-Ethyl-1-H-indene	17059-50-6	151437	-	HC	polyclyclic aromatic HC	1382	0.45	129	1251	NA	900	838

Continued on next page

Table S4. Tentatively identified compounds from the particulate phase fraction of mainstream marijuana smoke (Continued)

Tentative Identification	CAS	Peak Area	Health Effects	Class	Subclass	$^{1}t_R$ (s)	$^{2}t_R$ (s)	Quant Mass	Exp. RI*	Lib. RI*	Reverse†	Similarity‡
Hexanoic acid, 2-methylbutyl ester	2601-13-0	183766	-	Oxy	ester	1382	0.505	99	1251	1251	930	915
Geraniol	106-25-2	1386581	-	Oxy	alcohol	1386	0.465	69	1253	1253	936	905
Benzene, (1,3-dimethylbutyl)-	19219-84-2	116244	-	HC	monocyclic aromatic HC	1388	0.37	105	1254	NA	828	778
Naphthalene, 2-ethyl-1,2,3,4-tetrahydro-	32367-54-7	51651	-	HC	polycyclic aromatic HC	1390	0.395	104	1255	NA	839	799
Naphthalene, 1,2-dihydro-3-methyl-	2717-44-4	98985	-	HC	polycyclic aromatic HC	1394	0.445	129	1258	NA	823	762
Phenol, 4-propyl-	645-56-7	366802	-	Oxy	alcohol	1402	0.465	107	1262	1262	869	831
Benzene, hexyl-	1077-16-3	246007	-	HC	monocyclic aromatic HC	1404	0.38	92	1263	1263	871	865
Picolinamide	1452-77-3	643635	-	Het	pyridine	1410	0.955	79	1267	1268	948	883
Quinoline, 5,6,7,8-tetrahydro-	10500-57-9	88336	-	Nit	aza-arene	1412	0.555	132	1268	NA	869	852
1-(5-methylfurfuryl)-Pyrrole	13678-52-9	392724	-	Het	pyrrole	1424	0.5	95	1275	NA	938	773
1H-Indole, 1-methyl-	603-76-9	66277	-	Nit	aza-arene	1424	0.695	131	1275	1273	865	779
Phenol, 2,3,5-trimethyl-	697-82-5	117015	-	Oxy	alcohol	1426	0.475	121	1276	1276	864	721
1,4-Benzenedicarbonitrile	623-26-7	323451	-	Nit	nitrile	1430	1.495	128	1279	NA	945	793
Phenol, 4-ethyl-2-methoxy-	2785-89-9	183169	-	Oxy	alcohol	1430	0.61	137	1279	1278	917	886
5H-Benzocycloheptene, 6,7-dihydro-	7125-62-4	89144	-	HC	alicyclic HC	1432	0.435	129	1280	1269	858	814
1,12-Tridecadiene	21964-48-7	301849	-	HC	alkene	1440	0.315	55	1284	1279	909	865
1H-Pyrrole, 1-phenyl-	635-90-5	50635	-	Het	pyrrole	1442	0.53	143	1285	NA	865	774
1,2,3-Trimethylindene	4773-83-5	167351	-	HC	alicyclic HC	1444	0.42	143	1286	NA	866	858
Bornyl acetate	76-49-3	279818	-	Oxy	ester	1448	0.615	95	1289	1289	933	928
Phenol, 2-ethenyl-, acetate	63600-35-1	176968	-	Oxy	ester	1448	0.76	120	1289	NA	916	810

Continued on next page

Table S4. Tentatively identified compounds from the particulate phase fraction of mainstream marijuana smoke (Continued)

Tentative Identification	CAS	Peak Area	Health Effects	Class	Subclass	$^{1}t_{R}$ (s)	$^{2}t_{R}$ (s)	Quant Mass	Exp. RI*	Lib. RI*	Reverse†	Similarity‡
1H-Inden-1-one, 2,3-dihydro-	83-33-0	364862	-	Oxy	ketone	1448	0.845	104	1289	1285	943	857
1H-Indene, 1-ethylidene-	2471-83-2	51136	-	HC	polyclyclic aromatic HC	1450	0.545	141	1290	NA	913	767
2-Tridecene, (Z)-	41446-58-6	1079417	-	HC	alkene	1454	0.305	56	1292	1296	932	920
Thymol	89-83-8	25723	-	Oxy	alcohol	1456	0.42	135	1293	1293	935	770
Carvacrol	499-75-2	131905	X	Oxy	alcohol	1460	0.45	135	1296	1296	901	852
Indole	120-72-9	9507460	-	Nit	aza-arene	1466	0.655	90	1299	1299	936	925
Tridecane	629-50-5	1134358	-	HC	alkane	1468	0.29	57	1300	1300	959	947
(1-Methylbuta-1,3-dienyl)benzene	54758-36-0	128719	-	HC	monocyclic aromatic HC	1468	0.465	129	1300	NA	857	811
2-Ethyl-4,5-dimethylphenol	2219-78-5	132802	-	Oxy	alcohol	1472	0.44	135	1303	1305	873	799
3-Tridecene, (E)-	41446-57-5	128663	-	HC	alkene	1474	0.295	69	1304	NA	903	875
Cinnamonitrile	4360-47-8	672520	-	Nit	nitrile	1474	0.96	129	1304	1301	928	917
Naphthalene, 2-methyl-	91-57-6	1077813	-	HC	polycyclic aromatic HC	1478	0.54	142	1307	1307	948	942
2-Methyl-6-propylphenol	3520-52-3	44651	-	Oxy	alcohol	1484	0.435	121	1310	1320	909	768
2,5-Diethylphenol	876-20-0	125693	-	Oxy	alcohol	1488	0.43	135	1313	NA	882	810
1H-Inden-1-one, 2,3-dihydro-2-methyl-	17496-14-9	68883	-	Oxy	ketone	1490	0.76	131	1314	NA	898	847
1-Ethyl-2-Heptylcyclopropane	74663-86-8	30232	-	HC	alicyclic HC	1492	0.295	70	1315	NA	881	785
2-Methoxy-4-vinylphenol	7786-61-0	1673080	M	Oxy	ether	1494	0.635	135	1316	1316	927	922
p-Isopropenylacetophenone	06/04/5359	133698	-	Oxy	ketone	1496	0.475	145	1318	1308	810	782
Naphthalene, 1-methyl-	90-12-0	675002	-	HC	polycyclic aromatic HC	1504	0.53	142	1323	1323	946	936

Continued on next page

Table S4. Tentatively identified compounds from the particulate phase fraction of mainstream marijuana smoke (Continued)

Tentative Identification	CAS	Peak Area	Health Effects	Class	Subclass	$^{1}t_R$ (s)	$^{2}t_R$ (s)	Quant Mass	Exp. RI*	Lib. RI*	Reverse†	Similarity‡
5-Isopropenyl-2-methylcyclopent-1-enecarboxaldehyde	NA	65082	-	Oxy	ketone	1508	0.615	150	1325	NA	839	811
1-Methylindan-2-one	35587-60-1	52433	-	Oxy	ketone	1510	0.85	131	1327	NA	875	802
1-(3-Methylphenyl)4-methyl-3-pentene	51082-26-9	112549	-	HC	monocyclic aromatic HC	1524	0.365	105	1335	NA	884	827
Triacetin	102-76-1	184532	-	Oxy	ester	1528	1.145	103	1338	1339	959	949
4-Methylpyrrolo[1,2-a]pyrazine	64608-60-2	90679	-	Nit	nitrile	1532	0.775	132	1340	1340	917	814
1H-Indenol	56631-57-3	102269	-	Oxy	alcohol	1538	0.53	132	1344	NA	909	825
1,5,9-Undecatriene, 2,6,10-trimethyl-, (Z)-	62951-96-6	165119	-	HC	alkene	1540	0.31	69	1345	1350	886	855
Dodecane, 2,7,10-trimethyl-	74645-98-0	273806	-	HC	alkane	1544	0.335	57	1347	NA	862	751
3-Terpinolenone	491-09-8	73662	-	Oxy	ketone	1544	0.82	150	1347	1347	922	901
Heptylcyclohexane	5617-41-4	26543	-	HC	alicyclic HC	1546	0.31	83	1348	1347	839	778
Benzoic acid, 2-amino-, methyl ester	134-20-3	650948	-	Oxy	ester	1548	0.63	119	1350	1347	885	765
Phenol, 2,6-dimethoxy-	91-10-1	245761	-	Oxy	ether	1552	0.71	154	1352	1352	918	912
Eugenol	97-53-0	75040	G3	Oxy	alcohol	1562	0.575	164	1358	1358	875	852
Naphthalene, 1,2-dihydro-1,1,6-trimethyl-	30364-38-6	144734	-	HC	polycyclic aromatic HC	1570	0.41	142	1363	1364	870	833
3-Pyridinecarbonitrile, 1,4-dihydro-	23974-91-6	290044	-	Nit	nitrile	1576	1.04	105	1367	NA	905	774
Benzene, heptyl-	1078-71-3	181573	-	HC	monocyclic aromatic HC	1578	0.37	92	1368	1369	885	855
(Z)-8-Hydroxylinalool	64142-78-5	326946	-	Oxy	alcohol	1578	0.565	71	1368	1370	823	792
Piperitenone oxide	3564-96-3	130137	-	Oxy	epoxide	1578	0.87	67	1368	1371	842	813

Continued on next page

Table S4. Tentatively identified compounds from the particulate phase fraction of mainstream marijuana smoke (Continued)

Tentative Identification	CAS	Peak Area	Health Effects	Class	Subclass	^1t_R (s)	^2t_R (s)	Quant Mass	Exp. RI*	Lib. RI*	Reverse†	Similarity‡
Tridecane, 3-methyl-	6418-41-3	61286	-	HC	alkane	1582	0.295	57	1371	1371	843	815
1,3-Dimethyl-1H-indole	875-30-9	60390	-	Nit	aza-arene	1588	0.625	144	1375	1383	865	785
Farnesane	3891-98-3	486656	-	HC	sesquiterpene	1590	0.305	71	1376	1376	929	919
Gernayl acetate	105-87-3	283776	-	Oxy	ester	1592	0.485	69	1377	1377	905	897
alpha-Ylangene	14912-44-8	1052081	-	HC	sesquiterpene	1596	0.35	105	1379	1379	922	920
1,13-Tetradecadiene	21964-49-8	112649	-	HC	alkene	1604	0.31	82	1384	1385	933	902
Hexanoic acid, hexyl ester	6378-65-0	175801	-	Oxy	ester	1604	0.47	99	1384	1384	945	928
1,4-Dimethylazulene	1127-69-1	37865	-	HC	alicyclic HC	1606	0.52	141	1386	1390	875	772
alpha-Copaene	3856-25-5	284522	-	HC	sesquiterpene	1606	0.345	119	1385	1384	940	926
Biphenyl	92-52-4	423669	G2A	HC	polycyclic aromatic HC	1612	0.48	154	1389	1391	934	922
Quinoline, 4-methyl-	491-35-0	31256	M	Nit	aza-arene	1614	0.66	143	1391	1391	907	785
1-Tetradecene	1120-36-1	754658	-	HC	alkene	1618	0.3	69	1393	1393	951	941
alpha-Cubebene	17699-14-8	321520	-	HC	sesquiterpene	1618	0.345	105	1393	1396	833	829
1H-Pyrrole, 1-(4-methylphenyl)-	827-60-1	41146	-	Het	pyrrole	1622	0.52	157	1395	NA	870	793
1H-Indole, 3-methyl-	83-34-1	5674462	-	Nit	aza-arene	1622	0.61	130	1395	1395	927	926
Tetradecane	629-59-4	1183079	-	HC	alkane	1630	0.29	57	1400	1400	947	943
5-methyl-1H-Indole	614-96-0	537250	-	Nit	aza-arene	1634	0.625	130	1403	1397	903	896
1-Ethylnaphthalene	1127-76-0	347995	-	HC	polycyclic aromatic HC	1638	0.51	141	1406	1406	919	898
2-Methylbiphenyl	643-58-3	28297	X	HC	polycyclic aromatic HC	1638	0.405	168	1406	1405	899	857
Sesquithujene	159407-35-9	339361	-	HC	sesquiterpene	1640	0.315	119	1407	1408	922	915
(+)-Sativene	3650-28-0	738704	-	HC	sesquiterpene	1640	0.38	108	1407	1405	848	848
trans-beta-Caryophyllene	87-44-5	162306	-	HC	sesquiterpene	1654	0.37	93	1416	1416	943	925

Continued on next page

Table S4. Tentatively identified compounds from the particulate phase fraction of mainstream marijuana smoke (Continued)

Tentative Identification	CAS	Peak Area	Health Effects	Class	Subclass	$^{1}t_R$ (s)	$^{2}t_R$ (s)	Quant Mass	Exp. RI*	Lib. RI*	Reverse†	Similarity‡
Naphthalene, 2,6-dimethyl-	581-42-0	168521	-	HC	polycyclic aromatic HC	1656	0.53	156	1417	1416	928	896
Naphthalene, 1,7-dimethyl-	575-37-1	126413	-	HC	polycyclic aromatic HC	1660	0.525	156	1420	1419	942	910
trans-alpha-Bergamotene	17699-05-7	769891	-	HC	sesquiterpene	1660	0.33	93	1420	1419	956	939
beta-Copaene	20479-06-5	53597	-	HC	sesquiterpene	1662	0.36	120	1421	1422	852	827
Naphthalene, 2-ethenyl-	827-54-3	75111	-	HC	polycyclic aromatic HC	1666	0.515	153	1424	NA	917	799
alpha-Santalene	512-61-8	639943	-	HC	sesquiterpene	1670	0.34	94	1426	1424	939	928
(-)-Aristolene	6831-16-9	829626	-	HC	sesquiterpene	1674	0.355	105	1429	1429	877	877
Naphthalene, 1,3-dimethyl-	575-41-7	258060	-	HC	polycyclic aromatic HC	1678	0.525	141	1432	1427	914	905
cis-beta-Caryophyllene	118-65-0	10066986	-	HC	sesquiterpene	1678	0.405	41	1431	1427	943	942
N-methylphthalimide	550-44-7	110804	-	Nit	imide	1682	0.895	161	1434	1425	922	859
Naphthalene, 1,5-dimethyl-	571-61-9	372178	-	HC	polycyclic aromatic HC	1686	0.52	156	1437	1438	952	941
gamma-Elemene	339154-91-5	142760	-	HC	sesquiterpene	1686	0.34	121	1437	1437	786	777
cis-alpha-Bergamotene	18252-46-5	4752831	-	HC	sesquiterpene	1690	0.335	93	1439	1439	937	934
alpha-Guaiene	01/12/3691	114731	-	HC	sesquiterpene	1698	0.345	105	1444	1444	851	832
Sesquisabinene	58319-04-3	151396	-	HC	sesquiterpene	1702	0.34	69	1447	1444	818	800
3,5-Dimethoxy-4-hydroxytoluene	07/05/6638	41316	-	Oxy	ether	1702	0.685	168	1447	1447	853	786
Geranyl acetone	3796-70-1	306253	-	Oxy	ketone	1704	0.58	43	1448	1448	881	859
Aromadendrene	489-39-4	837650	-	HC	sesquiterpene	1708	0.34	105	1451	1447	866	863

Continued on next page

Table S4. Tentatively identified compounds from the particulate phase fraction of mainstream marijuana smoke (Continued)

Tentative Identification	CAS	Peak Area	Health Effects	Class	Subclass	^1t_R (s)	^2t_R (s)	Quant Mass	Exp. RI*	Lib. RI*	Reverse†	Similarity‡
1,4-Dimethylnaphthalene	571-58-4	166033	-	HC	polycyclic aromatic HC	1712	0.545	141	1454	1445	863	792
trans-beta-Farnesene	18794-84-8	6043308	-	HC	sesquiterpene	1712	0.325	69	1454	1453	941	937
Isoeugenol	97-54-1	240482	X	Oxy	alcohol	1714	0.605	164	1455	1455	945	920
Alloaromadendrene	25246-27-9	493144	-	HC	sesquiterpene	1722	0.355	161	1460	1461	899	897
Metolcarb	1129-41-5	114387	M	Misc	carbamate	1728	0.45	108	1464	1465	864	756
Acenaphthylene	208-96-8	220557	M	HC	polycyclic aromatic HC	1730	0.595	152	1465	1466	934	863
cis-beta-Farnesene	28973-97-9	231849	-	HC	sesquiterpene	1730	0.34	69	1465	1460	863	856
(-)-Isoledene	95910-36-4	150364	-	HC	sesquiterpene	1732	0.37	133	1467	NA	831	820
2(1H)-Pyridinone, 1-(3-oxo-1-cyclohexen-1-yl)-	69914-13-2	163091	-	Het	pyridine	1734	0.37	133	1468	NA	965	841
alpha-Humulene	6753-98-6	2261781	-	HC	sesquiterpene	1734	0.4	41	1468	1468	943	920
Isocadinene	16729-00-3	63314	-	HC	sesquiterpene	1736	0.35	159	1469	1472	814	761
Valencene	03/07/4630	269086	-	HC	sesquiterpene	1736	0.365	133	1469	1467	809	808
Bornyl butyrate	13109-70-1	32081	-	Oxy	ester	1740	0.54	71	1472	1470	905	767
epi-beta-Caryophyllene	68832-35-9	748493	-	HC	sesquiterpene	1742	0.37	91	1473	1471	928	909
Pyridine, 3-phenyl-	1008-88-4	71024	-	Het	pyridine	1746	0.62	155	1476	1470	907	828
Diethylpropion (amfepramone)	90-84-6	660488	-	Misc	amphetamine	1748	1.15	100	1477	1477	749	634
gamma-Muurolene	30021-74-0	1844412	-	HC	sesquiterpene	1758	0.35	105	1483	1482	907	900
4-Dodecanol	10203-32-4	35507	-	Oxy	alcohol	1760	0.305	54	1485	1482	803	777
gamma-Gurjunene	22567-17-5	1163756	-	HC	sesquiterpene	1764	0.355	91	1487	1487	909	894
Benzonitrile, 2,4,6-trimethyl-	2571-52-0	1085818	-	Nit	nitrile	1768	0.57	130	1490	NA	856	849
trans-beta-Bergamotene	15438-94-5	123034	-	HC	sesquiterpene	1770	0.35	133	1491	1493	863	815
cis-muurola-4(15),5-diene	157477-72-0	106835	-	HC	sesquiterpene	1772	0.33	105	1492	1490	754	728

Continued on next page

Table S4. Tentatively identified compounds from the particulate phase fraction of mainstream marijuana smoke (Continued)

Tentative Identification	CAS	Peak Area	Health Effects	Class	Subclass	^1t_R (s)	^2t_R (s)	Quant Mass	Exp. RI*	Lib. RI*	Reverse†	Similarity‡
1,1’-Biphenyl, 4-methyl-	644-08-6	137194	-	HC	polycyclic aromatic HC	1776	0.47	168	1495	1492	928	906
4a,8-Dimethyl-2-(prop-1-en-2-yl)-1,2,3,4,4a,5,6,7-octahydronaphthalene	103827-22-1	1212529	-	HC	sesquiterpene	1776	0.36	133	1495	1492	933	915
(-)-Myrtenol	19894-97-4	226589	-	Oxy	alcohol	1776	0.37	108	1495	NA	796	770
alpha-Muurolene	5951-61-1	312165	-	HC	sesquiterpene	1778	0.34	161	1496	1499	843	843
Acenaphthene	83-32-9	64072	G3	HC	polycyclic aromatic HC	1780	0.52	153	1498	1496	930	816
2,3-dimethyl-1H-Indole	91-55-4	382997	M	Nit	aza-arene	1782	0.645	144	1499	1507	926	908
Pentadecane	629-62-9	1643950	-	HC	alkane	1782	0.285	57	1499	1500	947	938
(-)-Zingiberene	495-60-3	405932	-	HC	sesquiterpene	1782	0.33	119	1499	1500	776	767
beta-Selinene	17066-67-0	837908	-	HC	sesquiterpene	1786	0.395	108	1502	1500	915	911
alpha-Farnesene	502-61-4	1603821	-	HC	sesquiterpene	1790	0.33	69	1504	1506	858	858
(+)-Eremophilene	NA	1275292	-	HC	sesquiterpene	1790	0.355	161	1504	1504	897	895
alpha-Selinene	473-13-2	3367916	-	HC	sesquiterpene	1794	0.375	93	1507	1508	946	939
1-Naphthalenecarbonitrile	86-53-3	88851	M	Nit	nitrile	1796	0.86	153	1509	1503	900	797
Naphthalene, 1-butyl-1,2,3,4-tetrahydro-	38857-76-0	1200430	-	HC	alicyclic HC	1798	0.455	131	1510	1518	781	781
beta-Bisabolene	495-61-4	2956361	-	HC	sesquiterpene	1802	0.32	69	1513	1511	890	890
delta-Cadinene	483-76-1	944522	-	HC	sesquiterpene	1802	0.335	161	1513	NA	837	830
Sesquicineol	90131-02-5	213493	-	Oxy	alcohol	1812	0.37	139	1520	1520	811	808
beta-Cadinene	523-47-7	1522493	-	HC	sesquiterpene	1822	0.33	161	1527	1523	866	864
Germacrene B	15423-57-1	2172288	-	HC	sesquiterpene	1822	0.35	121	1527	1523	795	783
beta-Sesquiphellandrene	20307-83-9	1007655	-	HC	sesquiterpene	1826	0.35	69	1529	1525	943	906
Bibenzyl	103-29-7	757715	-	HC	polycyclic aromatic HC	1828	0.45	91	1531	1528	915	902

Continued on next page

Table S4. Tentatively identified compounds from the particulate phase fraction of mainstream marijuana smoke (Continued)

Tentative Identification	CAS	Peak Area	Health Effects	Class	Subclass	^1t_R (s)	^2t_R (s)	Quant Mass	Exp. RI*	Lib. RI*	Reverse†	Similarity‡
trans-Calamenene	73209-42-4	500790	-	HC	sesquiterpene	1828	0.38	159	1531	1529	864	854
1-Naphthol	90-15-3	60444	X	Oxy	alcohol	1828	0.56	144	1531	1525	895	804
(-)-Eremophilene	10219-75-7	1989151	-	HC	sesquiterpene	1830	0.37	161	1532	1541	892	887
Dibenzofuran	132-64-9	193780	M	Het	furan	1832	0.56	168	1534	1532	854	817
Naphthalene, 1,4,5-trimethyl-	2131-41-1	66058	-	HC	polycyclic aromatic HC	1838	0.505	155	1538	1535	875	825
alpha-Gurjunene	489-40-7	1231424	-	HC	sesquiterpene	1846	0.34	204	1543	NA	858	854
Naphthalene, 2,3,6-trimethyl-	829-26-5	218894	-	HC	polycyclic aromatic HC	1848	0.505	155	1545	1550	923	905
trans-alpha-Bisabolene	25532-79-0	5867686	-	HC	sesquiterpene	1848	0.31	93	1545	1540	919	909
Pyridine, 4-(1-pyrrolidinyl)-	2456-81-7	40085	-	Het	pyridine	1850	0.775	147	1546	NA	881	750
alpha-Cadinene	24406-05-1	357461	-	HC	sesquiterpene	1852	0.345	105	1548	1546	850	845
alpha-Calacorene	21391-99-1	1088887	-	HC	sesquiterpene	1860	0.39	157	1553	1550	953	918
Selina-3,7(11)-diene	6813-21-4	1819658	-	HC	sesquiterpene	1864	0.37	204	1555	1545	910	908
Ochracin	17397-85-2	72999	-	Oxy	lactone	1866	1	134	1558	NA	851	790
Naphthalene, 1,4,6-trimethyl-	2131-42-2	106267	-	HC	polycyclic aromatic HC	1868	0.505	155	1559	NA	809	752
Nerolidol	40716-66-3	1247143	-	Oxy	alcohol	1874	0.39	93	1563	1564	920	919
Phenol, 4-ethenyl-2,6-dimethoxy-	28343-22-8	95679	-	Oxy	ether	1880	0.67	180	1567	1568	901	855
Pentadecane, 3-methyl-	2882-96-4	111179	-	HC	alkane	1884	0.29	57	1570	1569	855	815
Benzene, nonyl-	1081-77-2	102398	-	HC	monocyclic aromatic HC	1892	0.365	92	1575	1576	808	752
cis-6-Eudesmen-11-ol	194607-96-0	99512	-	Oxy	alcohol	1892	0.48	59	1575	1566	852	830
Germacrene D-4-ol	198991-79-6	342520	-	Oxy	alcohol	1906	0.47	43	1585	1579	830	814
1-Hexadecene	629-73-2	221613	-	HC	alkene	1916	0.295	83	1592	1592	940	938

Continued on next page

Table S4. Tentatively identified compounds from the particulate phase fraction of mainstream marijuana smoke (Continued)

Tentative Identification	CAS	Peak Area	Health Effects	Class	Subclass	^1t_R (s)	^2t_R (s)	Quant Mass	Exp. RI*	Lib. RI*	Reverse†	Similarity‡
3,5,11-Eudesmatriene	193615-07-5	119580	-	HC	sesquiterpene	1920	0.36	187	1595	NA	874	866
beta-Caryophyllene oxide	1139-30-6	1043419	-	Oxy	epoxide	1924	0.665	79	1598	1596	945	945
Hexadecane	544-76-3	597530	X	HC	alkane	1926	0.285	57	1599	1600	942	941
Fluorene	86-73-7	217051	G3	HC	polycyclic aromatic HC	1928	0.545	166	1600	1604	935	922
Viridiflorol	552-02-3	92975	-	Oxy	alcohol	1930	0.465	43	1602	1601	804	756
3-Hexadecene, (Z)-	34303-81-6	93822	-	HC	alkene	1932	0.285	55	1603	1604	916	849
Champacol	13822-35-0	252025	-	Oxy	alcohol	1936	0.45	59	1606	1602	884	878
Humulene epoxide 1	19888-33-6	118018	-	Oxy	epoxide	1946	0.63	93	1614	1610	874	852
Humulene epoxide 2	19888-34-7	393487	-	Oxy	epoxide	1962	0.645	96	1626	1620	918	917
beta-Vetivenene	27840-40-0	170951	-	HC	sesquiterpene	1964	0.365	187	1627	NA	879	874
alpha-Muurolol	19912-67-5	27353	-	Oxy	alcohol	1966	0.45	119	1629	1626	849	826
Selin-6-en-4-alpha-ol	118173-08-3	778591	-	Oxy	alcohol	1978	0.485	81	1638	1636	875	864
gamma-Eudesmol	1209-71-8	417823	-	Oxy	alcohol	1988	0.465	59	1645	1642	855	838
Pentadecane, 2,6,10-trimethyl-	3892-00-0	444915	-	HC	alkane	1990	0.3	57	1647	1649	910	903
Hexanoic acid, 2-phenylethyl ester	6290-37-5	81090	-	Oxy	ester	1990	0.525	104	1647	1649	848	761
(-)-Cubenol	21284-22-0	35271	-	Oxy	alcohol	1992	0.46	179	1648	1642	839	814
cis-Lanceol	10067-29-5	113386	-	Oxy	alcohol	2014	0.465	187	1665	NA	802	790
alpha-Cadinol	481-34-5	127624	-	Oxy	alcohol	2020	0.465	95	1669	1669	855	815
beta-Atlantol	38142-56-2	150890	-	Oxy	alcohol	2022	0.45	187	1671	NA	823	807
beta-Eudesmol	473-15-4	833555	-	Oxy	alcohol	2024	0.495	59	1672	1674	892	882
Bisabolene oxide A	NA	390747	-	Oxy	epoxide	2028	0.505	43	1675	1672	793	772
8-Heptadecene	16369-12-3	126569	-	HC	alkene	2034	0.295	69	1679	1680	870	841
Bulnesol	22451-73-6	177808	-	Oxy	alcohol	2034	0.455	107	1679	1668	910	903
1-Heptadecene	6765-39-5	235035	-	HC	alkene	2052	0.3	57	1693	1692	928	910

Continued on next page

Table S4. Tentatively identified compounds from the particulate phase fraction of mainstream marijuana smoke (Continued)

Tentative Identification	CAS	Peak Area	Health Effects	Class	Subclass	^1t_R (s)	^2t_R (s)	Quant Mass	Exp. RI*	Lib. RI*	Reverse†	Similarity‡
alpha-Bisabolol	515-69-5	1515188	-	Oxy	alcohol	2054	0.41	119	1694	1695	946	920
2-Pentadecanone	2345-28-0	87941	-	Oxy	ketone	2060	0.54	58	1699	1699	933	838
Heptadecane	629-78-7	651123	-	HC	alkane	2062	0.29	57	1700	1700	948	932
Pristane	1921-70-6	147651	X	HC	alkane	2068	0.305	57	1705	1707	885	871
Juniper Camphor	473-04-1	1295466	-	Oxy	alcohol	2082	0.465	43	1716	NA	898	897
9H-Fluorene, 1-methyl-	1730-37-6	88338	-	HC	polycyclic aromatic HC	2084	0.535	165	1717	1719	926	859
Isospathulenol	88395-46-4	361161	-	Oxy	alcohol	2096	0.44	43	1727	NA	847	844
3-Methyl-5-(2,6,6-trimethyl-1-cyclohexen-1-yl)-1-pentyn-3-ol	5417-74-3	47576	-	Oxy	alcohol	2102	0.635	55	1731	NA	792	769
Geranyl hexanoate	10032-02-7	542521	-	Oxy	ester	2126	0.425	69	1750	1756	912	912
Olivetol	500-66-3	1023266	M	Oxy	alcohol	2158	0.525	124	1774	1771	899	878
alpha-Muurolene-14-ol	81968-62-9	159779	-	Oxy	alcohol	2178	0.455	91	1790	1781	802	754
5-Octadecene, (E)-	7206-21-5	163652	-	HC	alkene	2184	0.3	57	1794	NA	931	927
Octadecane	593-45-3	333781	-	HC	alkane	2192	0.29	57	1800	1800	908	903
Anthracene	120-12-7	113566	G3	HC	polycyclic aromatic HC	2202	0.615	178	1809	1802	937	878
2-Phytene (cis)	NA	276471	-	HC	alkene	2230	0.31	70	1831	1835	882	869
Neophytadiene	504-96-1	2346734	-	HC	diterpene	2238	0.335	68	1838	1838	931	919
2-Phytene (trans)	2437-93-6	554433	-	HC	alkene	2244	0.31	70	1843	1838	893	889
2-Pentadecanone, 6,10,14-trimethyl-	502-69-2	324771	-	Oxy	ketone	2244	0.545	58	1843	1843	894	875
1-Nonadecene	18435-45-5	97546	-	HC	alkene	2306	0.305	83	1894	1893	926	903
Nonadecane	629-92-5	258567	-	HC	alkane	2314	0.295	57	1900	1900	933	921
beta-Springene	70901-63-2	655600	-	HC	alkene	2330	0.33	69	1914	NA	893	882
trans,trans-Farnesyl acetone	762-29-8	117190	-	Oxy	ester	2330	0.53	43	1914	1914	862	814

Continued on next page

Table S4. Tentatively identified compounds from the particulate phase fraction of mainstream marijuana smoke (Continued)

Tentative Identification	CAS	Peak Area	Health Effects	Class	Subclass	^1t_R (s)	^2t_R (s)	Quant Mass	Exp. RI*	Lib. RI*	Reverse†	Similarity‡
Hexadecanoic acid, methyl ester	112-39-0	664110	-	Oxy	ester	2344	0.42	74	1926	1926	954	911
gamma-Camphorene	20016-73-3	185632	-	HC	diterpene	2378	0.33	69	1955	1960	818	810
alpha-Springene	77898-97-6	165741	-	HC	alkene	2390	0.335	69	1965	1969	857	836
Cyclo-L-leu-L-pro	5654-86-4	162198	-	Nit	amino acid	2390	1.26	154	1966	NA	853	795
Cycro-(Pro-Pro)	19943-27-2	547300	-	Nit	amino acid	2396	1.19	70	1971	NA	937	842
alpha-Camphorene	20016-72-2	66922	-	HC	diterpene	2420	0.33	69	1990	1994	825	780
Hexadecanoic acid, ethyl ester	628-97-7	116092	-	Oxy	ester	2422	0.42	88	1992	1990	936	874
1-Heptadecanol	1454-85-9	48717	-	Oxy	alcohol	2424	0.31	83	1994	1986	905	877
Eicosane	112-95-8	171899	-	HC	alkane	2432	0.3	57	2000	2000	906	893
Heneicosane	629-94-7	133774	-	HC	alkane	2544	0.305	57	2100	2100	927	906
9,12,15-Octadecatrienoic acid, methyl ester, (Z,Z,Z)-	301-00-8	147680	-	Oxy	ester	2544	0.445	79	2100	2100	878	850
Methyl 9-octadecenoate	1937-62-8	39096	-	Oxy	ester	2550	0.43	74	2106	2107	926	878
Phytol	150-86-7	214271	-	Oxy	alcohol	2562	0.43	71	2117	2117	901	866
Pyrene	129-00-0	35113	G3	HC	polycyclic aromatic HC	2604	0.69	202	2157	NA	913	807
Linoleic acid ethyl ester	544-35-4	35937	-	Oxy	ester	2608	0.44	67	2161	2159	831	778
Hexadecanamide	629-54-9	266783	-	Nit	amide	2636	0.685	59	2187	2186	768	738
1-Docosene	1599-67-3	41488	-	HC	alkene	2644	0.315	57	2195	2194	911	881
Docosane	629-97-0	104426	-	HC	alkane	2650	0.31	57	2200	2200	914	844
trans-Ferruginol	514-62-5	978408	-	Oxy	alcohol	2780	0.49	203	2326	2325	761	761
Cannabivarin	33745-21-0	74634	-	Oxy	cannabinioid	2840	0.54	267	2383	2383	835	760
Cannabidiol	521-37-9	695605	M	Oxy	cannabinioid	2882	0.495	231	2424	NA	818	815
3-t-Butyl-6,6,9-trimethyl-6a,7,10,10a-tetrahydro-6H-benzo[c]chromen-1-ol	NA	250952	-	Oxy	alcohol	2884	0.485	217	2426	NA	790	776
Cannabichromene	20675-51-8	5405025	M	Oxy	cannabinioid	2888	0.46	231	2429	NA	900	891

Continued on next page

Table S4. Tentatively identified compounds from the particulate phase fraction of mainstream marijuana smoke (Continued)

Tentative Identification	CAS	Peak Area	Health Effects	Class	Subclass	$^{1}t_R$ (s)	$^{2}t_R$ (s)	Quant Mass	Exp. RI*	Lib. RI*	Reverse†	Similarity‡
delta-8-Tetrahydrocannabinol	5957-75-5	613499	M	Oxy	cannabinioid	2954	0.46	231	2493	NA	901	898
11-Hydroxy-delta-9-tetrahydrocannabinol	36557-05-8	149971	M	Oxy	cannabinioid	2968	0.5	299	2506	NA	774	764
delta-9-Tetrahydrocannabinol	03/08/1972	33950035	M	Oxy	cannabinioid	2982	0.55	231	2520	NA	790	774
Cannabigerol	25654-31-3	761069	-	Oxy	cannabinioid	3030	0.5	115	2566	NA	908	896
Cannabinol	521-35-7	945625	M	Oxy	cannabinioid	3038	0.57	310	2574	NA	812	781
Octacosane (no library hit)	630-02-4	75282	-	HC	alkane	3274	0.4	57	2800	2800	902	845
Octacosane, 2-methyl-	1560-98-1	346200	-	HC	alkane	3352	0.45	57	2875	2866	856	842
Tetracosyl benzoate	103569-99-9	325500	-	Oxy	ester	3486	1.59	123	3005	NA	863	753
Vitamin E	10191-41-0	219745	-	Oxy	alcohol	3506	0.79	165	3024	NA	835	817

*RI is Kovats Retention Index. “Exp. RI” refers to an experimental retention index calculated for this analysis. “Library RI” refers to a tabulated RI value obtained from NIST, PubChem, or ChemSpider electronic sources.
†Similarity refers to a "forward" mass spectral match score (i.e., how well does an experimentally-obtained spectrum fit to a library entry). In the case of a forward search, the match score (out of a maximum of 1000) for the experimental spectrum is penalized for containing extra peaks that are not present in the library spectrum.
‡Reverse refers to a "backwards" mass spectral match score (i.e., how well does a library entry fit to an experimentally-obtained spectrum). A reverse search match score (out of a maximum of 1000) for the library spectrum is not penalized for having extra peaks not contained in the experimental spectrum. A reverse search only requires that peaks in the library spectrum are present in the experimental spectrum.
Health effects were classified using the International Agency for Research on Cancer (IARC) carcinogen list and the Registry of Toxic Effects of Chemical Substances (RTECS) provided by the Canadian Centre for Occupational Health and Safety (CCOHS).
G1 = Group 1 carcinogen, G2A = Group 2A carcinogen, G2B = Group 2B carcinogen, G3 = Group 3 carcinogen, M = mutagen, T = teratogen, X = toxic by other mechanisms, “–“ = no risk data available/risks mitigated by proper protective equipment.
NA = not available, HC = hydrocarbon, Het = heterocycle, Misc = miscellaneous, Nit = nitrogenated, Oxy = oxygenated

SPME-GCxGC-TOFMS methods

GERSTEL MAESTRO Method

C:\ProgramData\Gerstel\Maestro\1\Methods\Tob-Cannb.1.mth

Method Information

No Method Information

Global Parameters

Runtime	:	60.00 min
Cool Down Time	:	7.00 min
Cryo Timeout	:	15.00 min

GERSTEL MAESTRO Method

C:\ProgramData\Gerstel\Maestro\1\Methods\Tob-Cannb.1.mth

MPS SPME Injection

Syringe Parameters

Syringe	:	Fiber SPME

Sample Preparation

SPME from Tray	:	disabled
Incubator	:	Agitator 1
Incubation Temp.	:	70 °C
Incubation Time	:	3.00 min
Agitator On Time	:	10 s
Agitator Off Time	:	1 s
Agitator Speed	:	250 rpm

Sample Parameters

Sample Tray Type	:	VT15/20-CVM
Vial Penetration	:	43.00 mm
Extraction Time	:	15.00 min
Inj. Penetration	:	54.00 mm
Desorption Time	:	120 s

Fiber Bakeout

Bakeout at	:	NONE

Derivatisation

Derivatisation Mode	:	No Derivatisation

Information

Hardware control:

- (●) Agilent© 7890 — Agilent© 7890 Gas Chromatograph
- ( ) Agilent© 6890 — Agilent© 6890 Gas Chromatograph
- ( ) Shimadzu© GC-2010 — Shimadzu© GC-2010
- ( ) Generic — Generic Gas Chromatograph
- ( ) Direct Inlet — Direct Inlet to Calibration Compound

Option:

- ☐ MACH/LTM Oven
- ☑ LECO© GCxGC

Capillary Configuration:

No problems detected with column configuration.

Flow Path 1:

#	Type	Location	Length(m)	Int. Diameter(µ)	Max Temp	Film Thickness	Phase	Bleed Masses
1*	Inlet	Front						
2	Capillary	GC Oven	60.000	250.00	350.0	0.25	Rtx-5Sil	73 149 207 281
3	Capillary	Modulator	0.100	250.00	340.0	0.25	Rtx-200l	73 149 207 281
4	Capillary	Secondary	1.290	250.00	340.0	0.25	Rtx-200l	
5	Capillary	Detector o	0.210	250.00	340.0	0.25	Rtx-200l	
6	Detector	TOF						

Add | Delete | Promote | Demote | Copy | Paste

☐ Enable Flow Path 2

Mass Selection for Auto Mass Defect Tracking

(Set Auto Mass Defect Mode in MS method. Select masses between 130 to 384 inclusive.)

(●) Excluded Masses in Auto Mass Defect Mode

(For general unknown analyses. Select column bleed, matrix, interfering, and non-target masses.)

( ) Included Masses in Auto Mass Defect Mode

(Generally for target analyses. Select significant masses of target analytes, minimum of 2 masses required.)

Carrier Gas:

Helium

Front Inlet Type:

Split / Splitless

Front Inlet Mode:

Split

Active Inlet Location:

(●) Front ( ) Back

The active inlet must be present in the capillary configuration.

No problems detected with pressure / flow.

☑ Corrected constant flow via pressure ramps

Use this mode when in GCxGC mode or using short (< 5 m) single column or two columns.

Column 1 / Front Inlet flow(s):

#	Rate (mL/min²)	Target Flow (mL/min)	Duration (min)
1*	Initial	2.00	Entire Run

Front Inlet Septum Purge Flow (mL/min):

3

0 - 30 mL/min

Column 1 / Front Inlet Split Ratio:

20

The ratio of split flow to column flow. This value cannot be used if your column is not defined (i.e. length, diameter, thickness).

Column 1 / Front Inlet Split Flow (mL/min):

40

Represents the flow from the split/purge vent. This value cannot be used if your column is not defined.

Column 1 / Front Inlet Total Flow (mL/min):

42

This is the total flow into the inlet, which is the sum of the split flow, and column flow. It does not include the septum purge flow. When the total flow is changed, the split ratio and split flow change while the column flow and pressure remain the same.

Column 1 / Front Inlet Gas Saver

◉ Yes ○ No

Column 1 / Front Inlet Gas Saver Flow (mL/min)

15

Column 1 / Front Inlet Gas Saver Time (minutes):

3.5

Front Inlet temperature(s):

#	Rate (°C/min)	Target Temp (°C)	Duration (min)
1*	Initial	250	Entire Run

No problems detected with oven temperatures.

Oven Equilibration Time (sec):

10

Note: All oven temperature ramps (except the secondary oven) will have the same duration. This is accomplished by extending the final hold time.

Enter oven temperature ramp below:

#	Rate (°C/min)	Target Temp (°C)	Duration (min)
1*	Initial	50	5.00
2	5.00	300	5.00

Add

Remove

Coolant to Column Oven ○ On ◉ Off Coolant timeout 10

☑ Enable Secondary Oven

Secondary Oven Temperature Offset (°C, relative to the GC oven temperature):

10

+5 °C is recommended

☐ Use Advanced Secondary Temperature Programming

☑ Modulator Enabled

Modulator Temperature Offset (°C, relative to the secondary oven temperature):

15

+15 °C is recommended

☐ Use Advanced Modulator Temperature Programming

Purge Pulse Time (sec):

0

Modulation T

For 1D GC set second dimension time to 0

#	Start	End	Modulation Period (s)	Hot Pulse Time	Cool Time Between Stages
1*	Start of Run	End of Run	2.00	0.30	0.70

Add Remove

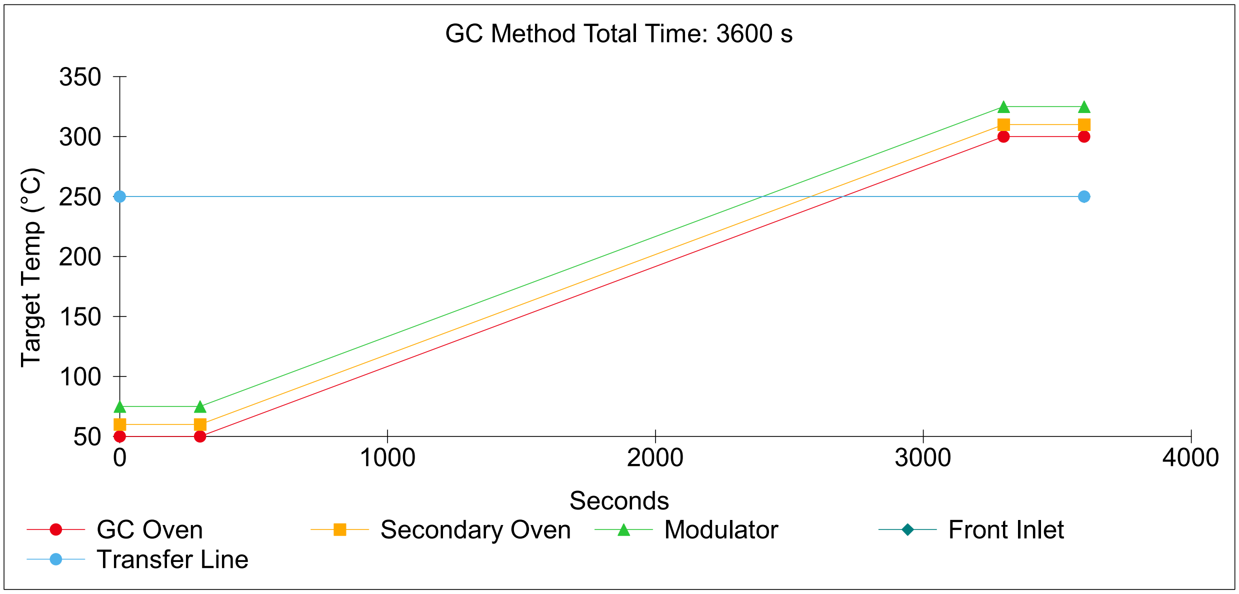

Transfer Line Temperature Equilibration Time (sec):

0

Transfer Line Temperature (°C):

250

☐ Specify Additional Detectors & Auxiliary Pneumatics

Use GC method total time for MS method total time:

(•) Yes ( ) No

Acquisition delay

30

(•) Sec.
( ) Min.

The length of time from injection until the data system will start storing data from the mass spectrometer.

Enter time(s) when the filament should be turned off (min of 3 sec) in the grid below

#	Start	End	Filament
1*	Start of Run	30 s	Off
2	30 s	End of Run	On

Add

Remove

Required Disk Space

NA

Enter the mass spectrometer settings:

Start Mass (u)

40

End Mass (u)

500

Acquisition Rate (spectra / second)

200

Detector Voltage:

[x] Specify Relative Detector Voltage

Optimized Voltage Offset: 200

Electron Energy (Volts)

-70

Mass defect mode

( ) Auto (Select masses for automatic tracking in column information section of GC method.)

(•) Manual

[x] Verify offset before collecting data

Mass Defect (mu / 100 u)

0

Set the temperature for the Ion Source.

Ion Source (°C)

200

☑ Wait for ion source temperatures to reach set point before starting acquisition

Source Temperature Equilibration Time (Seconds)

0

Enter the masses to display during acquisition

t

Examples

t TIC

69,131 Masses 69 and 131

69+131 Sum of masses 69 and 131

Select the task or tasks you wish to perform from the list below.

- [x] Baseline - computes baseline
- [x] Peak Find - finds peaks above the baseline using TSD®
- [x] Library Search - identifies all peaks found
- [x] Calculate Area / Height - computes the area and height of peaks without a calibration
- [x] Retention Index Method
- [ ] Classifications
- [ ] Apply Calibration(s) - computes the absolute concentration of peaks based upon a calibration
- [ ] Apply Reference(s) - computes the relative concentration of peaks with respect to a reference
- [ ] Semi Quantification - computes concentration based on another analytes calibration curve
- [ ] Tune Check
- [ ] Tailing Factor Check - checks to see if the analytes have an acceptable peak shape
- [ ] Calibration Check
- [ ] Blank Check - checks to make sure none of the analytes exceed their blank concentration
- [ ] Report - prints selected reports for each sample
- [x] Export peak information in ASCII CSV format
- [ ] Export data in Andi MS format (.cdf)
- [ ] Export data file
- [ ] Cache script results

Enter baseline tracking info below:

#	Start	End	Mode
1*	Start of Run	End of Run	Default

Add

Remove

Enter the baseline offset below (0.5-3.0):

0.7

Examples:
0.5 Through the middle of the noise
1.0 Just above the noise

Enter the number of data points that should be averaged for smoothing below:

Auto

- [x] GCxGC Parameters

1st Dimension

Enter the expected peak width in seconds below.

- [ ] Peak widths broaden throughout the chromatographic run

Peak Width: 12 | Retention Time:

Peak Width values should be the expected number of slices multiplied by the modulation period. Typically, 3 to 6 slices per

12	

Peak Width values should be the expected number of slices multiplied by the modulation period. Typically, 3 to 6 slices per analyte are expected.

Example: 6 slices x 4 sec. modulation period = Peak Width of 24

2nd Dimension

Match Required to combine: 750

☐ Override the allowed second dimension R.T shift for combine

Early

Late

Enter the expected peak width in seconds below: (as measured from baseline to baseline)

☐ Peak widths broaden throughout the chromatographic run

Peak Width	Retention Time
0.1	

For broadening, two peak widths may be specified at two different retention times. All peak widths will be extrapolated from these two points.

Subpeak Settings

Minimum S/N: 6

Enter the minimum required S/N for the subpeak to be retained.

Integration Approach:

◉ Traditional

○ Adaptive

☑ Filter peaks by classification

WWMD

Add...

Remove

Enter the maximum number of unknown peaks to find: 100000

☐ Keep False Peaks

Enter segmented processing info below:

#	Start	End	Peak Find	S/N	Masses	Number of Apexing Masses
1*	Start of Run	End of Run	On	100.0		4

Add

Remove

Common masses in derivatized products:

[ ]

Library Identity Search Mode:
(●) Normal ( ) Quick

Library Search Mode:
(●) Forward ( ) Reverse

Enter the number of library hits to return:
20

Enter the masses to library search below:

[ ]

Examples	
*	all masses collected
31:99	masses 31 through 99
31:99,200:211	masses 31:99 and 200:211

Minimum molecular weight allowed:
40

Maximum molecular weight allowed:
500

Mass Threshold (Relative abundance of base ion (0 - 998))
0

Minimum similarity match before name is assigned (0 - 999)
0

Add the libraries to use for searching below:
mainlib
replib
W8N08

Add... | Remove | Promote | Demote

☐ Enable Additional Library Search Criteria

Enter mass to use for area / height calculation:
U

Examples	
U	Unique mass
T	TIC
A	Apexing masses
55	m/z 55 used for every peak

☐ Allow skimming off small riding peaks

Select the retention index method to convert retention time to retention index:

Ryan_Ben.Retention Index [Select ...]

☑ Check Retention Index Analytes

Criteria:

☐ Maximum allowed retention index variation. 10

Options:

☐ Update the retention times of retention index analytes.

Select directory and columns to export below:

Directory

G:\Cannabis Smoke-CSV\ [Browse...]

☐ Remove repetition from sample name when generating filename

Header: (Leave Blank if no header information is desired)

[Functions...]

Field Separator

○ Comma

◉ Tab

Filter

☑ Calculate area percentage from filtered peaks only

☑ Quantifieds

☑ Analytes ☑ Surrogates ☑ Internal Standards

☐ Match

☑ Out of Tolerance

☑ Contaminants / Unknowns

☑ Not Founds

☐ Group: ☐ Classifications:

☐ Total Row ☐ Second Dimension Peaks

Sort by:

(•) Ascending
( ) Descending

Then by:

(•) Ascending
( ) Descending

Then by:

(•) Ascending
( ) Descending

Information not exported

Group
Height
Hit
Hit #
IntegrationBegin
IntegrationEnd
Ion Ratio Mass 1 Response 1
Ion Ratio Mass 1 Response 2
Ion Ratio Mass 1 Response 3
Ion Ratio Mass 2 Response 1
Ion Ratio Mass 2 Response 2
Ion Ratio Mass 2 Response 3
Ion Ratio Masses 1
Ion Ratio Masses 2
Ion Ratio Masses 3
Ion Ratio Result 1
Ion Ratio Result 2
Ion Ratio Result 3
Mass Defect
Noise
Non-Saturated Apex (s)
Peak Number
Probability
Profile Purity
Purity
RF
RRT
Ratio
S/N
Sample
Sample Concentration
Similarity
Synonyms
Synonyms List
Weight

Exported Information

Add >>

<< Remove

Name
R.T. (s)
Retention Index
Type
UniqueMass
LibraryId
Library
Match
Reverse
Quant Masses
Quant S/N
Area
Integration Modified
Quantification
Full Width at Half Height

Promote Demote

Library Hits

☑ Export Library Hits as part of the information for each peak.

Number of library hits to export:

10

Information not exported		Exported Information
Exact Mass<br>Mass Defect<br>Sample	Add >><br><< Remove	Similarity<br>Reverse<br>Probability<br>CAS<br>Library<br>Id<br>Formula<br>Weight<br>Contributor<br>Synonyms List

Promote Demote

Spectra

☐ Export the peak true spectrum as part of the information for each peak.

Mass Threshold (Relative abundance of base ion (0 - 998))

[illegible]

References

1. Center for Tobacco Reference Products (CTRP). Reference Cigarette Program. *University of Kentucky* https://ctrp.uky.edu/home (2019).
2. TJI Report. Reference products used in tobacco and smoke analyses. *Tob. J. Int.* 150–154 (2013). https://www.coresta.org/sites/default/files/pages/tji0213-p150-154-refproducts.pdf.
3. Statistics Canada. Canada at a glance 2018 - population. https://www150.statcan.gc.ca/n1/pub/12-581-x/2018000/pop-eng.htm (2018).
4. USA Federal Trade Commission. Federal trade commission cigarette report for 2016. https://www.ftc.gov/system/files/documents/reports/federal-trade-commission-cigarette-report-2016-federal-trade-commission-smokeless-tobacco-report/ftc_cigarette_report_for_2016_0.pdf (2018).
5. US Department of Health and Human Services. Smoking and tobacco control: Monograph 13-Risks associated with smoking cigarettes with low machine-measured yields of tar and nicotine. https://cancercontrol.cancer.gov/brp/tcrb/monographs/13/m13_complete.pdf (2001).
6. Ito, H. *et al.* Nonfilter and filter cigarette consumption and the incidence of lung cancer by histological type in japan and the united states: analysis of 30-year data from population-based cancer registries. *Int. J. Cancer* **128**, 1918–1928, DOI: https://doi.org/10.1002/ijc.25531 (2011).
7. Mehra, R., Moore, B. A., Crothers, K., Tetrault, J. & Fiellin, D. A. The association between marijuana smoking and lung cancer: A systematic review. *Arch. Intern. Medicine* **166**, 1359–1367, DOI: https://doi.org/doi:10.1001/archinte.166.13.1359 (2006).
8. Hancox, R. J. *et al.* Effects of cannabis on lung function: a population-based cohort study. *The Eur. Respir. J.* **35**, 42–47, DOI: https://doi.org/10.1183/09031936.00065009 (2010).
9. Gargani, Y., Bishop, P. & Denning, D. Too many mouldy joints- marijuana and chronic pulmonary aspergillosis. *Mediterr. J. Hematol. Infect. Dis.* **3**, e2011005, DOI: https://doi.org/10.4084/MJHID.2011.005 (2011).
10. Atakan, Z. Marijuana as medicine? The science beyond the controversy. *BMJ* **323**, 171, DOI: https://doi.org/10.1136/bmj.323.7305.171/a (2001).
11. Rickert, W. S., Robinson, J. C. & Rogers, B. A comparison of tar, carbon monoxide and pH levels in smoke from marihuana and tobacco cigarettes. *Can. J. Public Heal. / Revue Can. de Sante'e Publique* **73**, 386–391 (1982). https://www.jstor.org/stable/41987875.
12. Wu, T.-C., Tashkin, D. P., Djahed, B. & Rose, J. E. Pulmonary hazards of smoking marijuana as compared with tobacco. *New Engl. J. Medicine* **318**, 347–351, DOI: https://doi.org/10.1056/NEJM198802113180603 (1988).
13. Tashkin, D. P. *et al.* Tar, CO and Δ 9-THC delivery from the 1st and 2nd halves of a marijuana cigarette. *Pharmacol. Biochem. Behav.* **40**, 657–661, DOI: https://doi.org/10.1016/0091-3057(91)90378-F (1991).
14. Moir, D. *et al.* A comparison of mainstream and sidestream marijuana and tobacco cigarette smoke produced under two machine smoking conditions. *Chem. research toxicology* **21**, 494–502, DOI: https://doi.org/10.1021/tx700275p (2007).
15. Tashkin, D. P. *et al.* Effects of varying marijuana smoking profile on deposition of tar and absorption of CO and delta-9-THC. *Pharmacol. Biochem. Behav.* **40**, 651–656, DOI: https://doi.org/10.1016/0091-3057(91)90377-E (1991).
16. Health Canada. Canada government tobacco act: Tobacco reporting regulations, SOR/2000-273, Part 3: Emissions from designated tobacco products. https://laws-lois.justice.gc.ca/PDF/SOR-2000-273.pdf (2019).
17. ISO3308:2012. Routine analytical cigarette-smoking machine – Definitions and standard. International Organization for Standardization https://www.iso.org/standard/60404.html (2012).
18. ISO3402:1999. Tobacco and tobacco products - Atmosphere for conditioning and testing. International Organization for Standardization https://www.iso.org/standard/28324.html (1999).
19. TSI Inc. Series 3080 electrostatic classifiers operation and service manual, Revision J (2009).
20. Johnson, T. J., Irwin, M., Symonds, J. P. R., Olfert, J. S. & Boies, A. M. Measuring aerosol size distributions with the aerodynamic aerosol classifier. *Aerosol Sci. Technol.* **52**, 655–665, DOI: https://doi.org/10.1080/02786826.2018.1440063 (2018).
21. Dickau, M. *et al.* Methodology for quantifying the volatile mixing state of an aerosol. *Aerosol Sci. Technol.* **50**, 759–772, DOI: https://doi.org/10.1080/02786826.2016.1185509 (2016).
22. Johnson, T. J. *et al.* Steady-state measurement of the effective particle density of cigarette smoke. *J. Aerosol Sci.* **75**, 9–16, DOI: https://doi.org/10.1016/j.jaerosci.2014.04.006 (2014).
23. Hinds, W. C. *Aerosol technology: properties, behavior, and measurement of airborne particles* (John Wiley & Sons, 1999), 2 edn.
24. Swanson, J. & Kittelson, D. Evaluation of thermal denuder and catalytic stripper methods for solid particle measurements. *J. Aerosol Sci.* **41**, 1113–1122, DOI: https://doi.org/10.1016/j.jaerosci.2010.09.003 (2010).

25. Agarwal, J. K. & Sem, G. J. Continuous flow, single-particle-counting condensation nucleus counter. *J. Aerosol Sci.* **11**, 343 – 357, DOI: https://doi.org/10.1016/0021-8502(80)90042-7 (1980).

26. Tavakoli, F. & Olfert, J. S. An instrument for the classification of aerosols by particle relaxation time: Theoretical models of the aerodynamic aerosol classifier. *Aerosol Sci. Technol.* **47**, 916–926, DOI: https://doi.org/10.1080/02786826.2013.802761 (2013).

27. Cambustion Ltd. Aerodynamic aerosol classifier (AAC) user manual (original instructions), Version 1.13 (2018).

28. TSI Inc. Model 3776 ultrafine condensation particle counter: Operation and service manual, Revision B (2006).

29. Knutson, E. & Whitby, K. Aerosol classification by electric mobility: apparatus, theory, and applications. *J. Aerosol Sci.* **6**, 443 – 451, DOI: https://doi.org/10.1016/0021-8502(75)90060-9 (1975).

30. Wang, S. C. & Flagan, R. C. Scanning electrical mobility spectrometer. *Aerosol Sci. Technol.* **13**, 230–240, DOI: https://doi.org/10.1080/02786829008959441 (1990).

31. He, M. & Dhaniyala, S. A multiple charging correction algorithm for scanning electrical mobility spectrometer data. *J. Aerosol Sci.* **61**, 13 – 26, DOI: https://doi.org/10.1016/j.jaerosci.2013.03.007 (2013).

32. Leppä, J., Mui, W., Grantz, A. M. & Flagan, R. C. Charge distribution uncertainty in differential mobility analysis of aerosols. *Aerosol Sci. Technol.* **51**, 1168–1189, DOI: https://doi.org/10.1080/02786826.2017.1341039 (2017).

33. ISO15900:2009. Determination of particle size distribution – differential electrical mobility analysis for aerosol particles. International Organization for Standardization https://www.iso.org/standard/39573.html (2009).

34. Olfert, J. & Collings, N. New method for particle mass classification—the couette centrifugal particle mass analyzer. *J. Aerosol Sci.* **36**, 1338 – 1352, DOI: https://doi.org/10.1016/j.jaerosci.2005.03.006 (2005).

35. Cambustion Ltd. Centrifugal particle mass analyser (CPMA) user manual (original instructions), Version 2.03 (2017).

36. McMurry, P. H., Wang, X., Park, K. & Ehara, K. The relationship between mass and mobility for atmospheric particles: A new technique for measuring particle density. *Aerosol Sci. Technol.* **36**, 227–238, DOI: https://doi.org/10.1080/027868202753504083 (2002).

37. Olfert, J. S., Symonds, J. P. R. & Collings, N. The effective density and fractal dimension of particles emitted from a light-duty diesel vehicle with a diesel oxidation catalyst. *J. Aerosol Sci.* **38**, 69–82, DOI: https://doi.org/10.1016/j.jaerosci.2006.10.002 (2007).

38. Stolzenburg, M. R. & McMurry, P. H. Accuracy of recovered moments for narrow mobility distributions obtained with commonly used inversion algorithms for mobility size spectrometers. *Aerosol Sci. Technol.* **52**, 614–625, DOI: https://doi.org/10.1080/02786826.2018.1455963 (2018).

39. Stolzenburg, M. R. & McMurry, P. H. Equations governing single and tandem DMA configurations and a new lognormal approximation to the transfer function. *Aerosol Sci. Technol.* **42**, 421–432, DOI: https://doi.org/10.1080/02786820802157823 (2008).

40. Johnson, T. J., Olfert, J. S., Yurteri, C. U., Cabot, R. & McAughey, J. Hygroscopic effects on the mobility and mass of cigarette smoke particles. *J. Aerosol Sci.* **86**, 69–78, DOI: https://doi.org/10.1016/j.jaerosci.2015.04.005 (2015).

41. Ingebrethsen, B. J., Cole, S. K. & Alderman, S. L. Electronic cigarette aerosol particle size distribution measurements. *Inhalation Toxicol.* **24**, 976–984, DOI: https://doi.org/10.3109/08958378.2012.744781 (2012).

42. U.S. Department of Health and Human Services. NIDA research monograph 99: Research findings on smoking of abused substances. https://archives.drugabuse.gov/sites/default/files/monograph99.pdf (1990).
